# Transient Supramolecular Polymers by pH‐Gated Conformational Control of a Self‐Assembling Cyclodextrin

**DOI:** 10.1002/anie.202507069

**Published:** 2025-05-27

**Authors:** Wenting Hu, Valérian Libérioux, Julien Rossignol, Gaëlle Pembouong, Etienne Derat, Mickaël Ménand, Laurent Bouteiller, Matthieu Sollogoub

**Affiliations:** ^1^ Sorbonne Université, CNRS Institut Parisien de Chimie Moléculaire, IPCM Paris F‐75005 France; ^2^ Institut Universitaire de France (IUF) France

**Keywords:** Conformational control, Cyclodextrin, Kinetic control, Supramolecular polymer

## Abstract

Linking a cyclodextrin (CD) host to a hydrophobic guest can result in two distinct conformations: an introverted form (*in*), in which the guest is self‐included within the CD cavity, and an extraverted form (*out*), which enables intermolecular interactions and thus the formation of a supramolecular polymer. In this study, we demonstrate that a subtle variation of the linker enables interconversion between these two conformations, the *in* conformer being thermodynamically the most stable in water. At basic pH (>8) the *out* conformer is instantly converted into the *in*. In contrast, at acidic pH (<2), the *out* monomer can be kinetically trapped and can self‐assemble into a supramolecular polymer. DFT calculations reveal that the interconversion mechanism is governed by a key hydrogen bond that locks the conformational states. Furthermore, we show that pH provides fine kinetic control over the interconversion rate and, consequently, the polymerization process. The system can then be reset toward the *out* conformation by using DMSO. This system stands in contrast to known transient supramolecular polymerization processes, which rely on metastable (non‐assembled) monomers. Here, it is the kinetic trapping of the assembling monomer that allows control over the lifetime of the transient supramolecular polymer via a pH‐responsive mechanism.

## Graphical abstract

A metastable cyclodextrin monomer can form kinetically trapped supramolecular polymer at low pH. Raising the pH triggers gradual depolymerization into the self‐included monomer with a rate that can be preset by tuning the pH value. The pH‐dependence is directly associated with the rigidity of the cyclodextrin bridge, which is modulated by protonation.

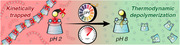

## Introduction

Linking monomers through reversible interactions to form long chains of molecules (i.e., supramolecular polymers)^[^ ^1^ ^]^ is a challenging but rewarding goal because their dynamic nature provides them with responsiveness toward a range of stimuli that has contributed to the development of self‐healing materials,^[^ ^2^ ^]^ bioinspired systems,^[^ ^3^ ^]^ smart gels,^[^ ^4^ ^]^ innovative catalysts,^[^ ^5^ ^]^ and optoelectronic materials,^[^ ^6^ ^]^ among other applications.

After focusing on the design of thermodynamically stable supramolecular polymers, the field has recently shifted to their kinetic control through dissipative or non‐dissipative non‐equilibrium systems. In the former case, assembly is only favored in the presence of an energy source (photon or fuel molecule) which drives the system away from thermodynamic equilibrium and allows the properties of these materials to be controlled in space and time by the kinetics of the coupled reactions.^[^ ^7^ ^]^ In the latter case, the kinetic control of competing metastable or trapped assemblies (pathway complexity) has paved the way for supramolecular polymers of controlled length, narrow dispersity and block copolymer structure (living supramolecular polymerization).^[^ ^8^, ^9^, ^10^ ^]^ For example, in the case of perylene bisimide^[^ ^11^ ^]^ or corannulene^[^ ^12^ ^]^ platforms, it has been shown that kinetic trapping of the (non‐assembled) monomer is an effective strategy for controlling supramolecular polymerization kinetics.

Among the various building blocks known to afford supramolecular polymers in water,^[^ ^13^ ^]^ cyclodextrins (CDs) are of particular interest as they form relatively strong host–guest interactions with hydrophobic groups, in addition to being biobased and non‐toxic. While many CD‐based supramolecular polymers have been described in the literature,^[^ ^14^ ^]^ the kinetic control of their assembly remains to be achieved.

**Figure 1:**
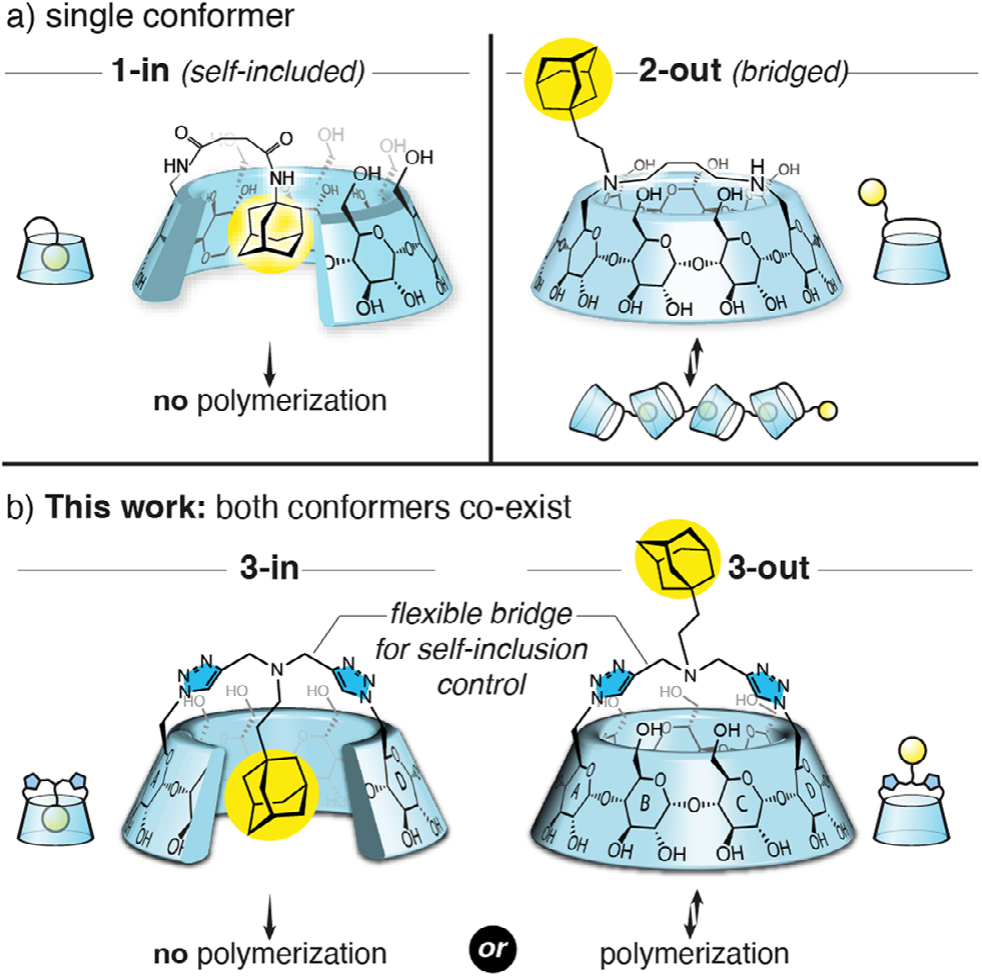
Comparative design of adamantyl functionalized CD monomers. a) Previous designs without bridge leading to self‐inclusion (**1‐in**) or with a rigid bridging avoiding self‐inclusion (**2‐out**). b) Present design involving a longer bridge allowing both self‐inclusion (**3‐in**) or self‐assembly (**3‐out**).

The design of supramolecular polymers with CD is usually achieved by linking the CD host to a hydrophobic guest.^[^ ^14^ ^]^ However, the way the guest is conjugated onto the CD is critical, as self‐inclusion^[^ ^15^, ^16^, ^17^, ^18^, ^19^, ^20^, ^21^ ^]^ is a common competitive pathway.^[^ ^22^, ^23^ ^]^ We also showed that simply linking an adamantyl group to the primary rim of β‐CD affords the self‐included compound **1‐in** (Figure 1a).^[^ ^24^ ^]^ At the time, we believed, based on literature,^[^ ^25^, ^26^ ^]^ that self‐inclusion of this large guest was only possible through the large rim of the CD through a tumbling process. With this caveat in mind, we then designed a bridged CD **2‐out** bearing the guest on one side of the bridge, and showed that self‐inclusion was completely suppressed and isodesmic self‐assembly could occur (Figure 1a).^[^ ^27^ ^]^

In the framework of our work in this area, we describe herein a CD **3** bearing a larger bridge incorporating 3 additional atoms compared to **2‐out**, with a central adamantyl group which fortuitously allows the co‐existence of both conformers **3‐in** and **3‐out**. Their interconversion was further found to be solvent‐dependent and pH‐gated, offering a kinetic control/trapping of the supramolecular polymerization of **3‐out** (Figure 1b) contrasting with previous systems involving the kinetic trapping of the non‐assembled monomer.^[^ ^11^, ^12^ ^]^

## Results and Discussion

### Synthesis

**Scheme 1:**
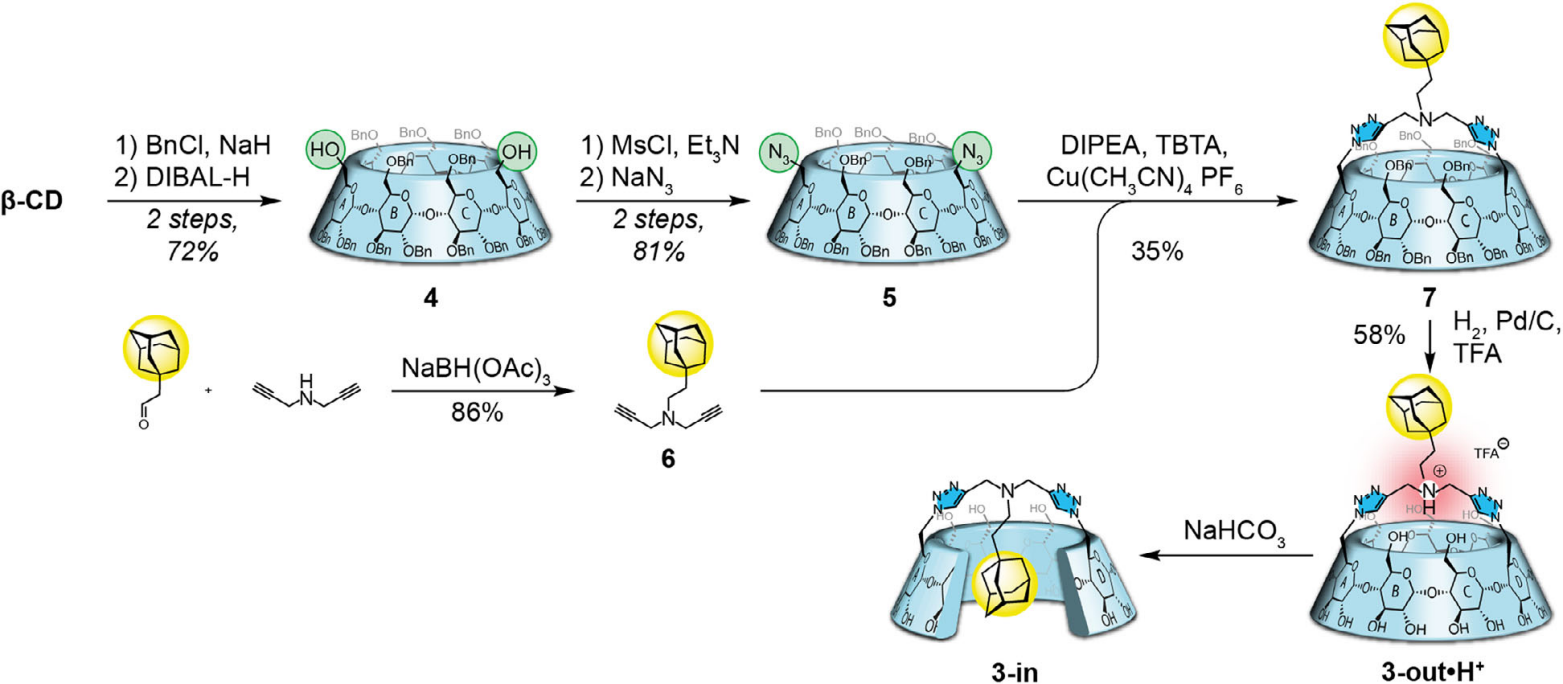
Synthesis of CD **3**.

The synthesis of compound **3** began with the classical perbenzylation/bis‐debenzylation sequence to give diol **4** in 72% yield over two steps.^[^ ^28^, ^29^, ^30^ ^]^ Bis‐azido **5** was then obtained in two steps from **4** through a mesylation of the diol and nucleophilic substitution by an azide of the formed mesylates in 81% yield over two steps.^[^ ^31^ ^]^ Meanwhile, we synthesized the bisalkyne **6** through reductive amination of adamantane acetaldehyde with dipropargylamine in 86% yield. Copper‐catalyzed azide‐alkyne cycloaddition (CuAAC) reaction was carried out in the presence of compound **5** to form the bridged CD **7** in a relatively moderate 35% yield. Final deprotection by catalytic hydrogenation in the presence of palladium on carbon and TFA gave **3‐out•H^+^** (Scheme 1). However, upon neutralization (from pH < 2 to pH > 8), **3‐in** was obtained, with the adamantyl included inside the CD cavity. This came as a surprise since the inclusion of the adamantyl group through the primary rim is supposed to be disfavored,^[^ ^25^, ^26^ ^]^ and even excluded with the bridging of this rim,^[^ ^27^ ^]^ so we sought to understand this observation.

**Figure 2:**
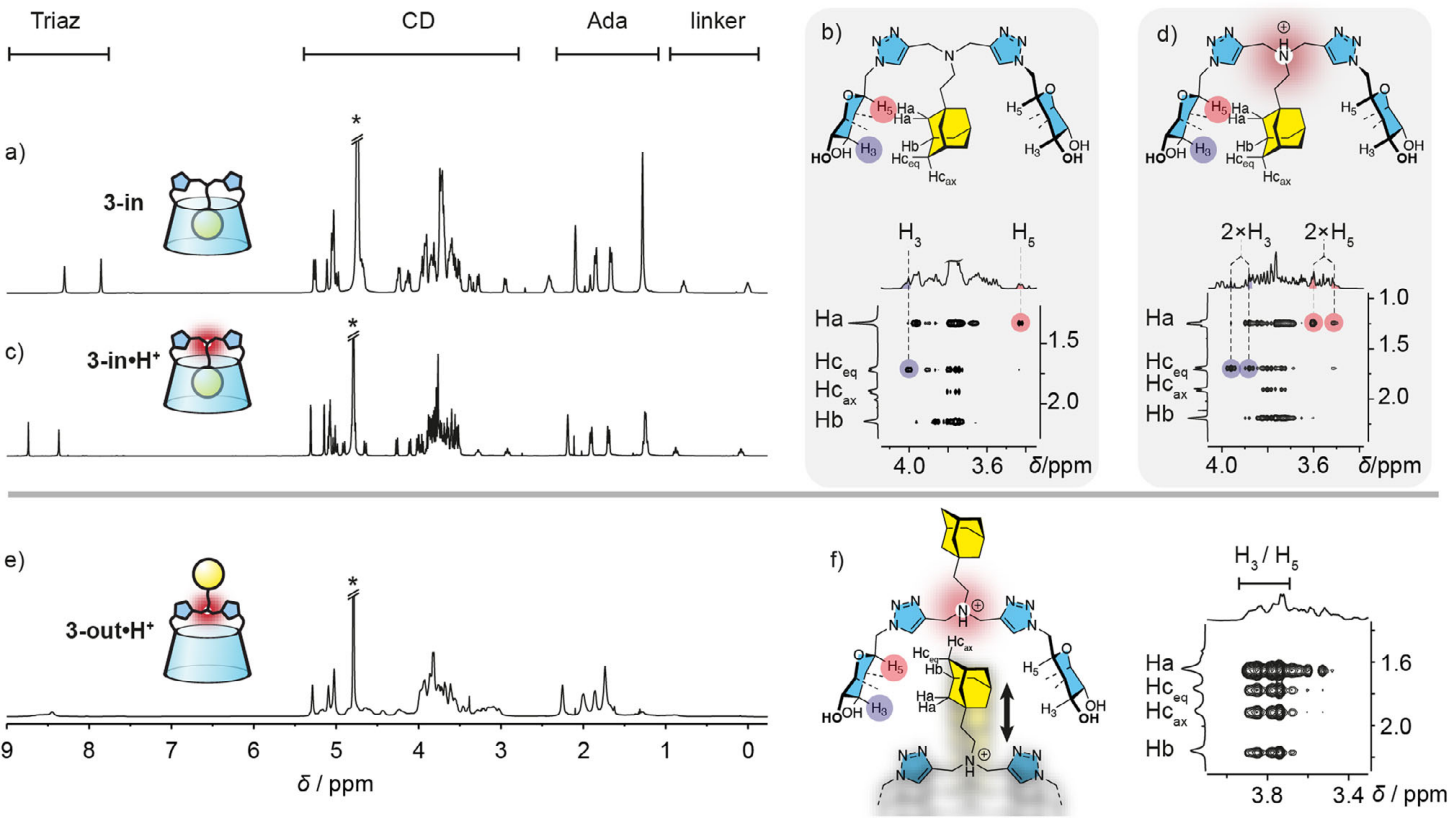
^1^H‐NMR and selected regions of ROESY spectra (D_2_O, 600 MHz, 300 K) of a,b) CD **3‐in** (5 mM, pH 9.1), c–d) CD **3‐in•H^+^** (5 mM, pH 1.8); e,f) CD **3‐out•H^+^** (5 mM, pH 2.1).

^1^H‐NMR spectra of both isomers in D_2_O display a striking resolution difference (Figure 2). Indeed, the self‐included CD **3‐in** shows a sharp signature (Figure 2a), while the CD **3‐out•H^+^** displays a broad one (Figure 2e). The behavior of this latter is due to its ability to dynamically self‐assemble into large species, which slows their tumbling motion, alters their relaxation properties, and results in signal broadening. In contrast, the well resolved ^1^H NMR spectrum of CD **3‐in** indicates that this isomer does not assemble in solution because of the self‐inclusion of the adamantyl group. This introverted conformation prevents self‐assembly. This was further evidenced by NOESY NMR of **3‐in** showing a specific cross‐correlations pattern between the CD and the adamantyl group (Ada), i.e., H‐5^CD^/Ha^Ada^ and H‐3^CD^/Hc^Ada^ (Figure 2b). This pattern was previously observed and attributed to self‐inclusion.^[^ ^24^ ^]^ In contrast, for **3‐out•H^+^**, both H‐3^CD^ and H‐5^CD^ cross‐correlate with all the protons of the adamantyl group (Figure 2f), probably due to the dynamic assembly process that results in varying distances between these protons. These observations clearly demonstrate that the adamantyl group is included in both isomers, through an introverted orientation (self‐included **3‐in**) or an extraverted orientation with intermolecular inclusion (**3‐out•H^+^**).

We therefore have a system able to self‐assemble into supramolecular polymers when pH < 2 (**3‐out•H^+^**) and self‐include into dispersed CD monomers at pH > 8 (**3‐in**). We then studied the parameters influencing the conformational interconversion equilibrium of CD **3**. We first studied the influence of pH in water. For instance, we protonated the self‐included conformer **3‐in** hoping to force the expulsion of the adamantyl group and convert it back to the self‐assembling **3‐out•H^+^**, but only **3‐in•H^+^** was obtained as shown by monitoring the NMR behavior of **3‐in** in D_2_O with increasing amounts of TFA (up to three equivalents, Figures 2c and Supporting Information 3). Thus, decreasing the pH leads to the deshielding of signals corresponding to the triazole protons and the three methylene groups surrounding the central nitrogen, while those connected to the triazole units (CH_2_‐6) are not affected (see Supporting Information 3 for details). This indicates that the protonation preferentially occurs on the central nitrogen.^[^ ^32^ ^]^ Besides, no other significant shifts are observed upon acidification and the T‐ROESY spectrum of the acidified sample still shows the characteristic correlations pattern of the self‐included conformer **3‐in•H^+^** (Figure 2d). We then heated the solution of **3‐in•H^+^** in acidic D_2_O for 1 week at 90°C, but did nor observe any change in the NMR spectrum (Supporting Information 4.1). Conversely, heating **3‐out•H⁺** in acidic D₂O (pH 2, 50°C, 4 days; see Supporting Information 4.2) results in a slow conversion to **3‐in•H⁺**, reaching >90% conversion. Additionally, upon deprotonation of **3‐out•H⁺** (pH > 8), a rapid and complete conversion to **3‐in** is observed. These results demonstrate that, in aqueous solution, the introverted conformers **3‐in** and **3‐in•H⁺** are thermodynamically the most stable. In contrast, **3‐out•H⁺** is a metastable species at room temperature, undergoing slow transformation into its introverted counterpart upon heating.

**Figure 3:**
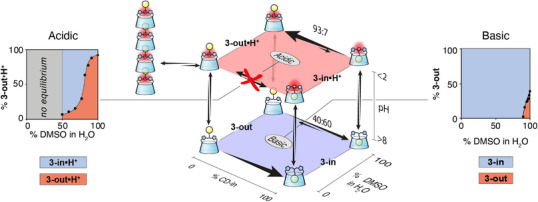
Solvent and pH‐dependent thermodynamic network.

To shift this equilibrium toward the **3‐out** conformer, we next varied the solvent polarity since hydrophobic inclusion complexes, especially those based on CDs, typically dissociate in organic media. We therefore recorded a ^1^H NMR spectrum of the neutral CD **3‐in** in pure DMSO, observing immediate equilibration between the **3‐in** and **3‐out** conformers in a 60:40 ratio, respectively, in slow exchange on the NMR time scale (Figure 3 right and Supporting Information 5.1). Remarkably, when the DMSO solution is made acidic, equilibration still occurs but at a slower rate and the ratio of **3‐in•H^+^:3‐out•H^+^** becomes 7:93 after a week at room temperature, or after 12 h at 50°C (Figure 3 left and Supporting Information 5.1). This experiment allows us to reset the system by evaporating the acidic DMSO to recover **3‐out•H^+^** from **3‐in**.

Since DMSO strongly influences the *in*/*out* equilibrium, we further studied this equilibrium at various DMSO:D_2_O ratios in either acidic or basic conditions. In basic conditions (pH > 8), from pure water to a 9:1 mixture of DMSO:D_2_O, the CD **3‐out** conformer rapidly converts (*t* < 2 min) into pure **3‐in** conformer. Increasing further the DMSO proportion allows to set the equilibrium with up to 40% of the **3‐out** conformer in pure DMSO (Figure 3 right and Supporting Information 5.2). It is interesting to note that the hydrophobic interaction responsible for the adamantyl group self‐inclusion, known to be very sensitive to the polarity of the medium,^[^ ^33^, ^34^ ^]^ remains dominant over the range of polarity investigated. However, the situation is quite different under acidic conditions (pH < 2). No equilibration occurs from pure water to 50% DMSO at room temperature (Figure 3 left and Supporting Information 5.3). For example, if conformer **3‐out•H^+^** is dissolved in a 40% DMSO aqueous solution, no equilibration at room temperature will occur. Increasing the proportion of DMSO above 50% triggers a slow equilibrium between the two isomers, with the equilibrium ratio depending on the proportion of DMSO (*k*_in→out_ = 0.7 (±0.6) x 10^−6^ s^−1^ with 80% DMSO versus 8.3 (±0.3) x 10^−6^ s^−1^ with 100% DMSO, Supporting Information 5.3.4). Ultimately, in pure DMSO, 93% of **3‐out•H^+^** isomer is observed (*K* = 15.7, Figure 3 left and Supporting Information 5.3).

The *in* or *out* conformation of CD **3** is therefore dependent both on the pH and the solvent (Figure 3). In water, the *in* conformation is the thermodynamically preferred conformation both at basic or acidic pH, while the *out* conformation can be kinetically trapped at acidic pH at room temperature and convert to the *in* conformer only at higher temperature. In DMSO, the *in*/*out* equilibrium is faster with a preference for the *out* conformation at acidic pH.

**Figure 4:**
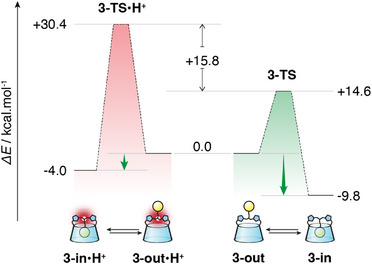
Evolution of the relative energetic profiles (DFT//DFTB) of the conversion of CD **3‐in** into CD **3‐out** with or without protonation (TS: transition state).

To study the rather remarkable effect of protonation, kinetically trapping the CD **3‐out•H^+^** conformer in water, we decided to use DFTB calculations since the system is relatively large. We thus selected the lowest energetic conformer **3‐in** to study the extrusion of the adamantyl group out of the CD cavity. Then, we placed an anchor point away from the CD (a methane at 30 Å) and calculated the displacement of the adamantyl group toward this anchor point. The calculations were performed in implicit water and followed by a single‐point DFT calculation on each state (see Supporting Information 6.1). We then studied the protonated form introducing the proton on the central amine and using the same protocol. The corresponding energetic profiles are presented in Figure 4 and Supporting Information 6.2. Rewardingly, the calculation is in accordance with the experiments as the self‐included conformer is thermodynamically the most favored one for both the neutral and the protonated forms (Δ*G* ^0^ = −9.8 and −4.0 kcal mol^−1^ respectively). However, the inclusion of the adamantyl of **3‐out**•**H^+^** necessitates a much higher activation energy than for the unprotonated **3‐out** (ΔΔG^‡^ = 15.8 kcal mol^−1^), consistent with the slow conversion observed at 50°C and confirming the kinetic trapping of the extraverted conformation **3‐out**•**H^+^** at room temperature.

**Scheme 2:**
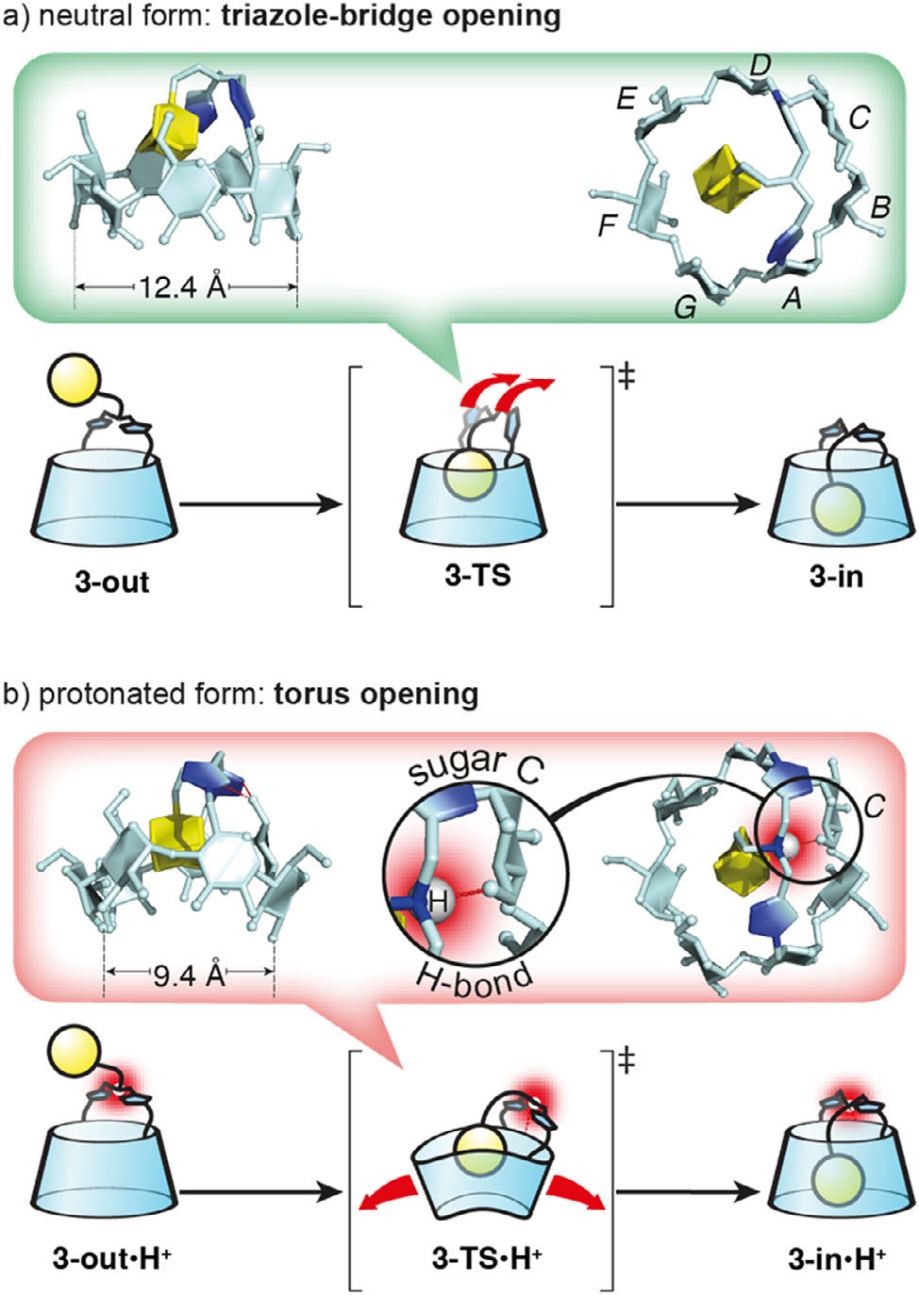
Illustration of the self‐inclusion mechanism of compound **3** in its neutral a) and protonated b) forms. Arrows indicate the distortion of either the bridge a) or the CD torus b). DFT calculated transition state (TS) conformations of **3‐TS** (green inset) and **3‐TS•H^+^** (red inset) with selected distances between O‐2^B^ and O‐2^E^ highlighting the pinched conformation of **3‐TS•H^+^**. Circle: zoom showing the locking hydrogen bond.

We next studied the structural details of the respective mechanisms of self‐inclusion of CDs **3‐out** and **3‐out**•**H^+^**. Calculated 3D models of CDs **3‐out**, **3‐in** and of **3‐TS** corresponding to the transition state in the conformational interconversion (see Supporting Information 6.3), a simplified version is shown on Scheme 2. Interestingly, the mechanisms are clearly different. In the case of the unprotonated CD **3‐out**, the conversion into **3‐TS** only involves a movement of the bridge and almost no change in CD torus conformation (Scheme 2a and Supporting Information 6.3 top). More precisely, the triazole units rotate from a parallel to a perpendicular orientation compared to the plane of the CD, moving the amine toward sugars B and C. This geometry widens the loop delineated by the bridge and the E, F, G sugar units, allowing the adamantyl group to enter the CD cavity. After the self‐inclusion of the adamantyl group, the CD and the bridge recover their initial conformations. In the case of the protonated CD **3‐out**•**H^+^** the situation is different. In **3‐TS**•**H^+^** the ammonium proton forms a hydrogen bond with O‐6 of sugar C (Scheme 2b, red inset). This H‐bond provides a conformational restriction locking the bridge and preventing its displacement to allow the adamantyl group to pass through. Instead, it is the CD torus that undergoes the distortion, bending strongly to let the adamantyl group through (Scheme 2b and Supporting Information 6.3 bottom). We therefore propose that the difference in activation barriers between the two processes arises from changes in the bridge's flexibility upon protonation: while the neutral bridge remains mobile, protonation induces hydrogen bonding that rigidifies it, thereby forcing the CD torus to undergo energetically costly distortion during the transformation. For instance, conformational differences in the CD torus between the transition states are reflected in the distance between O‐2s of units B and F, which measures 12.4 Å in the unprotonated TS and 9.4 Å in the protonated TS (Scheme 2, insets). This original mechanism sheds light on an unexpected behavior of the adamantyl group capable of self‐inclusion through the primary rim of a bridged β‐CD with relative ease in water. This mechanism is possibly facilitated by the central position of the adamantyl group and the flexibility of the bridge.

**Figure 5:**
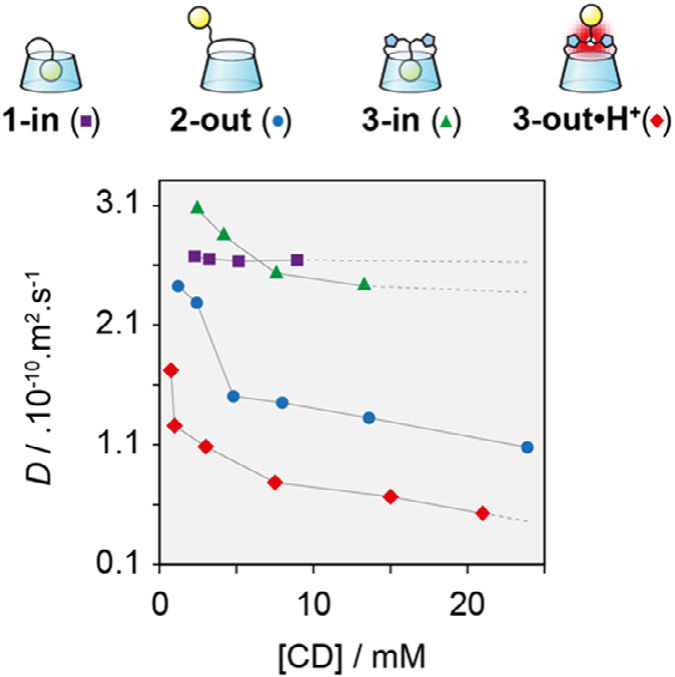
Concentration dependence of the diffusion coefficient (DOSY, 600 MHz, D_2_O, 300 K) for **1‐in**, **2‐out**, **3‐in**, and **3‐out•H^+^**.

We then studied in more details the self‐assembling properties of **3‐in** and **3‐out•H^+^** using DOSY‐NMR, plotting the diffusion coefficient (*D*) against concentration for each monomer (Figure 5). As expected, the diffusion coefficient of **3‐in** is hardly affected by the variation of concentration keeping roughly the same order of magnitude as previously observed for other self‐included monomers such as CD **1‐in**,^[^ ^24^ ^]^ and native β‐CD over the same range of concentration (i.e.*, D* = 2.6 × 10^−10^ m^2^ s^−1^).^[^ ^35^ ^]^ In contrast, an increase in concentration of **3‐out•H^+^** at pH <2 led to a nonlinear decrease of *D* indicating the growth of the supramolecular polymer with concentration. The degree of polymerization (DP) of compound **3‐out•H^+^** was determined according to the Tirado‐Garcia de la Torre method,^[^ ^36^ ^]^ reaching up to ca. 22 units at 21 mM (see Supporting Information 7). In addition, ITC measurements allowed to probe the self‐association mechanism of **3‐out•H^+^**.^[^ ^37^ ^]^ The data is in agreement with an isodesmic mechanism and afforded an association constant of *K* = 9.0 x 10^3^ M^−1^ (see Supporting Information 8) which is consistent with the DP obtained by DOSY. Interestingly, **3‐out•H^+^** forms assemblies of a similar size as our previously published bridged CD **2‐out** (Figure 5).^[^ ^27^ ^]^ The supramolecular polymerization was also confirmed by an increase in viscosity with concentration (see Supporting Information 9) and the presence of a q^−1^ slope in small angle neutron scattering, characteristic of the formation of rod‐like objects (see Supporting Information 10).

**Figure 6:**
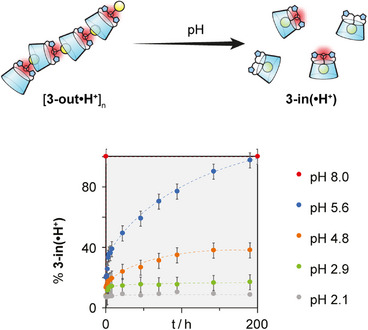
Time‐dependent conversion of self‐assembling **3‐out•H^+^** into self‐included monomer **3‐in(•H^+^)** at different concentrations and pH. Proportions determined by ^1^H NMR (600 MHz, D_2_O, 300 K).

So far, we studied the self‐assembly from the thermodynamic point of view, we next turned our attention to its kinetic component. As the protonation state of the central amine plays a central role in the process, we studied the influence of the pH on the assembly process. Prior to this study, we determined that the p*K*_a_ of the central amine was around 5 (see Supporting Information 11), which happens to be rather low for an aliphatic amine. While the assembly of CD **3‐out•H^+^** is stable in D_2_O at pH <2 with one set of broad signals in ^1^H NMR (see Supporting Information 12.3.5), raising the pH to 5.6 led to the gradual appearance, in slow exchange on the NMR time scale, of a second set of sharp signals corresponding to **3‐in•H^+^** (see Supporting Information 12.3.2). Over time, the extraverted isomer **3‐out•H^+^** is fully converted into the self‐included **3‐in•H^+^**. The kinetics of depolymerization/self‐inclusion were monitored by plotting the ratio of **3‐in(•H^+^)** to **3‐out(•H^+^)** conformers, determined from the integration of adamantyl proton signals corresponding to each conformer. Measurements were conducted at varying pH values (2.1, 2.9, 4.8, 5.6, and 8.0) while maintaining a constant concentration of 4.3 mM (Figure 6, Supporting Information 12.3).

The interconversion rates clearly stress the pH effect on the kinetics. We observe 100% conversion into self‐included conformer in less than 2 min at pH 8.0, while no conversion is observed after 190 h at pH 2.1, and intermediate values at pH around the p*K*_a_ of the central amine of CD **3**.

**Figure 7:**
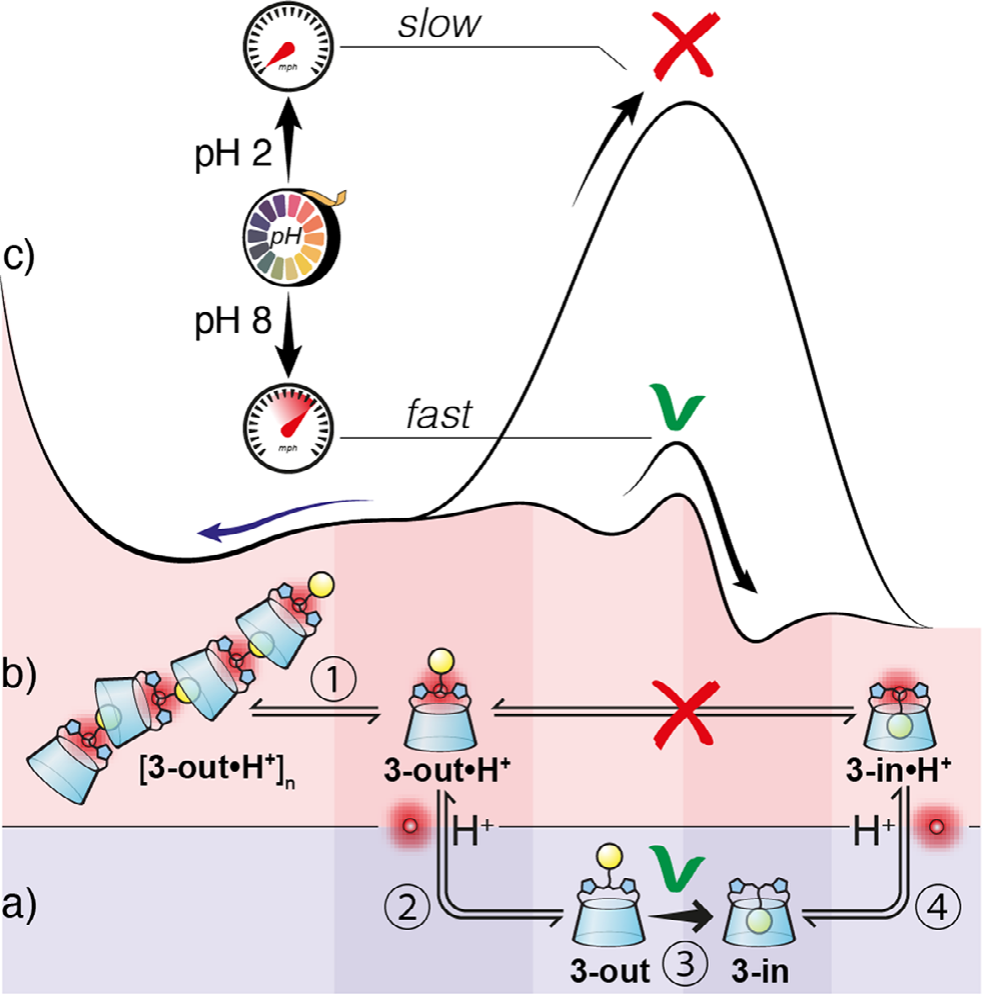
Energy landscape summarizing the pH‐dependent behavior of CD **3** in a) basic conditions [thermodynamic self‐inclusion] and b) acidic conditions [kinetically trapped supramolecular polymer] involved in the depolymerization pathway c).

**3‐out•H^+^** being kinetically trapped at room temperature, its depolymerization process has to occur through a mechanism involving the following steps (Figure 7a,b): 1) dissociation from the supramolecular polymer (ITC: Δ*G* = −7.6 kcal, Supporting Information 8), 2) deprotonation into **3‐out**, 3) self‐inclusion to form **3‐in** (DFT: Δ*G* = −9.8 kcal, SI.6.2), 4) protonation (depending on the pH) into **3‐in•H^+^**, in which the rate‐limiting step is the self‐inclusion of the adamantyl group. In acidic conditions (pH < 2), the concentration of **3‐out** is so small that the self‐inclusion is not detectable. At pH above 3, self‐inclusion becomes kinetically possible and competes with the polymerization, as the concentration of **3‐out** increases. Overall, the supramolecular polymer converts into the self‐included monomer at variable rates depending on pH and temperature. This means that the supramolecular polymer is less thermodynamically stable than the self‐included monomer (Figure 7).

## Conclusion

In conclusion, we report here a new host‐guest system, where the particular linker between the β‐CD host and the adamantyl guest allows to control the relative stabilities of a supramolecular polymer versus a self‐included monomer and the kinetics of depolymerization of the former into the latter. This original mechanism sheds light on an unexpected behavior of the adamantyl group capable of self‐inclusion through the primary rim of the CD. This self‐inclusion can be reversed in acidic conditions in DMSO. We further deciphered the key role of protonation which rigidifies the bridge and disfavors self‐inclusion. The pH‐gated pathway between the two isomers allows to kinetically trap the protonated **3‐out•H^+^** conformer which further assembles into a supramolecular polymer. Remarkably, this assembly turns out to be transient with a rate of disassembly being tuned over a large range of time (minutes to days) through pH control, which means that the “life‐span” of the supramolecular polymer can be pre‐programmed by the pH.

This result is particularly intriguing because transient supramolecular polymers are usually obtained in the case of dissipative supramolecular polymers: in the absence of fuel the assembly naturally falls apart.^[^ ^7^, ^8^ ^]^ The system reported here is clearly not dissipative; it is a simple isodesmic supramolecular polymer. However, the transient nature can be recovered by raising the pH which ultimately triggers the competition between polymerization and monomer deactivation, because the deactivated monomer is more stable than the supramolecular polymer but forms at a slower rate.

Overall, we show that the precise functionalization of the CD molecular brick allows to control its assembly/disassembly mechanism and kinetics. This pH‐dependent disassembly is of interest for delivery systems. Indeed, we already showed that similar self‐assembling CD was able to encapsulate cargoes such as siRNA and deliver it within the cell.^[^ ^27^ ^]^ With its pH dependency, the present system can be envisaged to target the gastrointestinal tract which undergoes a progressive increase in pH from the stomach to the colon, suitable for a controlled release of a cargo complexed with the CD assembly. ^[^ ^38^ ^]^

## Conflict of Interests

The authors declare no conflict of interest.

## Supporting information



Supporting information

## Data Availability

The data that support the findings of this study are available from the corresponding author upon reasonable request.

## References

[1] T. Aida , E. W. Meijer , S. I. Stupp , Science 2012, 335, 813–817. 22344437 10.1126/science.1205962 PMC3291483

[2] F. Herbst , D. Döhler , P. Michael , W. H. Binder , Macromol. Rapid Commun. 2013, 34, 203–220. 23315930 10.1002/marc.201200675

[3] O. J. G. M. Goor , S. I. S. Hendrikse , P. Y. W. Dankers , E. W. Meijer , Chem. Soc. Rev. 2017, 46, 6621–6637. 28991958 10.1039/c7cs00564d

[4] A. Dawn , T. Shiraki , S.‐I. Haraguchi , S.‐I. Tamaru , S. Shinkai , Chem. Asian J. 2011, 6, 266–282. 20715040 10.1002/asia.201000217

[5] Y. Li , L. Bouteiller , M. Raynal , ChemCatChem. 2019, 11, 5212–5226.

[6] S. S. Babu , V. K. Praveen , A. Ajayaghosh , Chem. Rev. 2014, 114, 1973–2129. 24400783 10.1021/cr400195e

[7] S. A. P. van Rossum , M. Tena‐Solsona , J. H. van Esch , R. Eelkema , J. Boekhoven , Chem. Soc. Rev. 2017, 46, 5519–5535. 28703817 10.1039/c7cs00246g

[8] A. Sorrenti , J. Leira‐Iglesias , A. J. Markvoort , T. F. A. de Greef , T. M. Hermans , Chem. Soc. Rev. 2017, 46, 5476–5490. 28349143 10.1039/c7cs00121e PMC5708531

[9] J. Matern , Y. Dorca , L. Sánchez , G. Fernández , Angew. Chem. Int. Ed. 2019, 58, 16730–16740. 10.1002/anie.201905724 PMC6900041 31271244

[10] M. Wehner , F. Würthner , Nat. Rev. Chem. 2020, 4, 38–53.

[11] S. Ogi , V. Stepanenko , K. Sugiyasu , M. Takeuchi , F. Würthner , J. Am. Chem. Soc. 2015, 137, 3300–3307. 25689054 10.1021/ja511952c

[12] J. Kang , D. Miyajima , T. Mori , Y. Inoue , Y. Itoh , T. Aida , Science. 2015, 347, 646–651. 25657246 10.1126/science.aaa4249

[13] W. Han , W. Xiang , Q. Li , H. Zhang , Y. Yang , J. Shi , Y. Ji , S. Wang , X. Ji , N. M. Khashab , J. L. Sessler , Chem. Soc. Rev. 2021, 50, 10025–10043. 34346444 10.1039/d1cs00187f

[14] A. Harada , Y. Takashima , H. Yamaguchi , Chem. Soc. Rev. 2009, 38, 875–882. 19421567 10.1039/b705458k

[15] L. Jullien , J. Canceill , L. Lacombe , J.‐M. Lehn , J. Chem. Soc. Perkin Trans. 1994, 2, 989–1002.

[16] R. Corradini , A. Dossena , R. Marchelli , A. Panagia , G. Sartor , M. Saviano , A. Lombardi , V. Pavone , Chem. Eur. J. 1996, 2, 373–381.

[17] J. W. Park , S. Y. Lee , H. J. Song , K. K. Park , J. Org. Chem. 2005, 70, 9505–9513. 16268626 10.1021/jo0515834

[18] Y. Han , K. Cheng , K. A. Simon , Y. Lan , P. Sejwal , Y.‐Y. Lu , J. Am. Chem. Soc. 2006, 128, 13913–13920. 17044719 10.1021/ja064591q

[19] T. Nakamura , S. Yonemura , T. Nabeshima , Chem. Commun. 2019, 55, 3872–3875. 10.1039/c9cc00517j 30801104

[20] A. Ueno , T. Kuwabara , A. Nakamura , F. Toda , Nature 1992, 356, 136–137.

[21] G. Fukuhara , T. Fujimoto , T. Kaneda , Chem. Lett. 2003, 32, 536–537.

[22] a) Y. Inoue , P. Kuad , Y. Okumura , Y. Takashima , H. Yamaguchi , A. Harada , J. Am. Chem. Soc. 2007, 129, 6396–6397. 17461590 10.1021/ja071717q

[23] Y. Liu , Z.‐X. Yang , Y. Chen , J. Org. Chem. 2008, 73, 5298–5304. 18549282 10.1021/jo800488f

[24] D. N. Tran , D. Colesnic , S. Adam de Beaumais , G. Pembouong , F. Portier , A. A. Queijo , J. Vazquez Tato , Y. Zhang , M. Ménand , L. Bouteiller , M. Sollogoub , Org. Chem. Front. 2014, 1, 703–706.

[25] J. Carrazana , A. Jover , F. Meijide , V. H. Soto , J. V. Tato , J. Phys. Chem. B 2005, 109, 9719–9726. 16852171 10.1021/jp0505781

[26] Y. Liu , C. Chipot , X. Shao , W. Cai , J. Phys. Chem. C 2014, 118, 19380–19386. 10.1021/jp508841p 25221951

[27] P. Evenou , J. Rossignol , G. Pembouong , A. Gothland , D. Colesnic , R. Barbeyron , S. Rudiuk , A.‐G. Marcelin , M. Ménand , D. Baigl , V. Calvez , L. Bouteiller , M. Sollogoub , Angew. Chem. Int. Ed. 2018, 57, 7753–7758. 10.1002/anie.201802550 29693753

[28] T. Lecourt , A. Herault , A. J. Pearce , M. Sollogoub , P. Sinaÿ , Chem. Eur. J. 2004, 10, 2960–2971. 15214078 10.1002/chem.200305683

[29] S. Guieu , M. Sollogoub , J. Org. Chem. 2008, 73, 2819–2828. 18335960 10.1021/jo7027085

[30] E. Zaborova , Y. Blériot , M. Sollogoub , Tetrahedron Lett. 2010, 51, 1254–1256.

[31] L. Duarte , S. Nag , M. Castro , E. Zaborova , M. Ménand , M. Sollogoub , V. Bennevault , J.‐F. Feller , P. Guégan , Macromol. Chem. Phys. 2016, 217, 1620–1628.

[32] P. Weßling , M. Trumm , E. Macerata , A. Ossola , E. Mossini , M. C. Gullo , A. Arduini , A. Casnati , M. Mariani , C. Adam , A. Geist , P. J. Panak , Inorg. Chem. 2019, 58, 14642–14651. 31609595 10.1021/acs.inorgchem.9b02325 PMC6863594

[33] C. Senac , S. Desgranges , C. Contino‐Pépin , W. Urbach , P. F. J. Fuchs , N. Taulier , ACS Omega 2018, 3, 1014–1021. 31457945 10.1021/acsomega.7b01212 PMC6641370

[34] B. Siegel , R. Breslow , J. Am. Chem. Soc. 1975, 97, 6869–6870.

[35] A. J. M. Valente , R. A. Carvalho , O. Söderman , Langmuir 2015, 31, 6314–6320. 26017565 10.1021/acs.langmuir.5b01493

[36] J. G. De La Torre , M. C. L. Martinez , M. M. Tirado , Biopolymers 1984, 23, 611–615.

[37] A. Arnaud , L. Bouteiller , Langmuir 2004, 20, 6858–6863. 15274596 10.1021/la049365d

[38] J. N. Chu , G. Traverso , Nat. Rev. Gastroenterol. Hepatol. 2022, 19, 219–238. 34785786 10.1038/s41575-021-00539-w PMC12053541

