# Supplementary material for: Transient Supramolecular Polymers by pH‐Gated Conformational Control of a Self‐Assembling Cyclodextrin
Source: Angew Chem Int Ed Engl. 2025 May 27;64(29):e202507069. doi: 10.1002/anie.202507069 (PMC12258658; doi:10.1002/anie.202507069)
Supplement: Supplementary file 1 — Supporting information [file ANIE-64-e202507069-s001.pdf]

Transient Supramolecular Polymers by pH-Gated Conformational Control of a Self-Assembling Cyclodextrin

Wenting Hu,[a] Valérian Libérioux,[a] Julien Rossignol,[a] Gaëlle Pembouong,[a] Etienne Derat,[a] Mickaël Ménand,*[a] Laurent Bouteiller,*[a] and Matthieu Sollogoub*[a,b]

[a] Sorbonne Université, CNRS, Institut Parisien de Chimie Moléculaire, IPCM, F-75005 Paris, France
[b] Institut Universitaire de France (IUF)

Table of contents

SI.1 General Procedures 3

SI.2 Synthesis and descriptions 4

SI.2.1 Nomenclature for protons 4

SI.2.2 6^A, 6^D-Dideoxy-6^A, 6^D-azido-2^{A-G}, 3^{A-G}, 6^B, 6^C, 6^E, 6^F, 6^G-enneadeca-*O*-benzyl-β-cyclodextrin (**5**) 5

SI.2.3 2-(adamantan-1-yl)acetaldehyde 9

SI.2.4 2-(adamantan-1-yl)-N,N-dipropargylethanamine (**6**) 9

SI.2.5 6^A, 6^D-Dideoxy-6^A, 6^D-ditriazole-bridged-*N*-ethyladamantyl-2^{A-G}, 3^{A-G}, 6^B, 6^C, 6^E, 6^F, 6^G-enneadeca-O-benzyl-β-cyclodextrin (**7**) 11

SI.2.6 6^A, 6^D-Dideoxy-6^A, 6^D-di-triazole-bridged-*N*-ethyladamantyl-β-cyclodextrin trifluoroacetate (**3-out•H$^+$**) 16

SI.2.7 6^A, 6^D-Dideoxy-6^A, 6^D-di-triazole-bridged-*N*-ethyladamantyl-β-cyclodextrin (**3-in**) 18

SI.2.8 6^A, 6^D-Dideoxy-6^A, 6^D-di-triazole-bridged-*N*-ethyladamantyl-β-cyclodextrin trifluoroacetate (**3-in•H$^+$**) 20

SI.3 ^{1}H NMR titration of **3-in** with TFA in D_2O 22

SI.4 ^{1}H NMR spectra of **3-in•H$^+$** and **3-in•H$^+$** in D_2O at variable temperatures 22

SI.4.1 ^{1}H NMR spectra of **3-in•H$^+$** in D_2O at variable temperatures 22

SI.4.2 ^{1}H NMR spectra of **3-out•H$^+$** in D_2O at variable temperatures 23

SI.5 Behavior of **3** in D_2O/DMSO-d_6 mixtures at pH < 2 and pH > 8 24

SI.5.1 **3-in**/**3-out** equilibration in 100% DMSO-d_6 24

SI.5.2 **3-in**/**3-out** equilibration in D_2O/DMSO-d_6 mixtures at pH > 8 25

SI.5.3 **3-in•H$^+$**/**3-out•H$^+$** equilibration in D_2O/DMSO-d_6 mixtures at pH < 2 27

SI.6 Theoretical study 37

SI.6.1 Procedure 37

SI.6.2 Energies 37

SI.6.3 DFT calculated conformations of the self-inclusion mechanism 38

SI.7 NMR DOSY - degree of polymerization (DP) 39

SI.8 ITC experiments – host/guest association 40

SI.9 Viscometry study of the assembly 41

SI.10 SANS study of the assembly 42

SI.11 pKa determination by ^{1}H NMR 43

SI.11.1 pKa of **3-in** in D_2O 43

SI.11.2 pKa of **3-out** in DMSO-d_6 44

SI.12 pH- and time-dependent of **3-out•H$^+$** into self-included monomer **3-in•H$^+$** 45

SI.12.1 Preparation of the samples 45

SI.12.2 Quantification method 45

SI.12.3 Time-dependent ^{1}H NMR spectra at variable pH 46

SI.13 XYZ Coordinates (DFTB) 51

SI.13.1 **3-in** 51

SI.13.2 **3-TS** 55

SI.13.3 **3-out** 59

SI.13.4 **3-in•H$^+$** 63

SI.13.5 **3-TS•H$^+$** 67

SI.13.6 **3-out•H$^+$** 71

SI.1 General Procedures

Reactants were purchased from commercial sources and used without further purification. The solvents were freshly distilled under N_2 by standard methods: dichloromethane from P_2O_5; THF and toluene from sodium/benzophenone; DMF and triethylamine were dried over molecular sieves. Thin Layer Chromatography (TLC) was performed on Merck silica gel 60 F 254 and revealed with a UV lamp (λ = 254 nm) and $KMnO_4$, cerium molybdate or sulphuric acid staining. Flash columns chromatography used silica Geduran® Si 60 Å (40 – 63 µm) or silica Geduran® Si 60 Å (63 – 200 µm).

The CombiFlash Rf system was employed by injecting 5 mL of the filtered aqueous extract onto a RediSep Rf Gold C-18 reversed-phase column (20-40 µm). Elution was performed by using pumps to deliver a constant flow rate of 20 mL/min. The solvent system consisted of acetonitrile (CH_3CN) and water always started with 0% of CH_3CN for 3 min to desalt the media then a specified gradient was applied for each product. The compounds including a phenyl group were detected by UV at 210 nm.

High-Resolution Mass Spectrometry (HRMS) was recorded on a Bruker micrOTOF spectrometer, using Agilent ESI-L Low Concentration Tuning-Mix as reference.

InfraRed (IR) spectra were recorded on a Bruker Tensor 27 (ATR diamond) spectrophotometer.

NMR spectra were recorded on a Bruker Avance II 600 MHz or Bruker AM-400 MHz using residual solvent signal as internal reference. Assignments of the signals were done using Heteronuclear Single Quantum Coherence spectroscopy (HSQC), Heteronuclear Multiple Bound Correlation (HMBC), COrrelation SpectroscopY (COSY), TOtal Correlation SpectroscopY (TOCSY), Nuclear Overhauser Effect SpectroscopY (NOESY), Transverse Rotating-frame Overhauser Enhancement SpectroscopY (T-ROESY). All sugar units are renamed from unit A to unit G in the counterclockwise direction (first rim). Chemical shifts (δ) are reported in ppm and coupling constants (J) are given in Hertz (Hz). Abbreviations used for peak multiplicity are: s (singlet); bs (broad singlet); d (doublet); t (triplet); q (quartet); quint (quintet); sept (septet); m (multiplet).

The DOSY NMR diffusion experiment was performed using the using bipolar longitudinal eddy current delay with gradients (LEDBPGP) sequence; spectra were acquired with gradient pulses (δ) of 2 ms ranging in strength from 0.28 to 5.26 g/mm for the BBFO 5mm NMR probe. A diffusion delay from 50 to 200 ms was set and diffusion coefficients (D) were calculated from mono-exponential decays using Bruker Topspin 3.0 software.

SI.2 Synthesis and descriptions

SI.2.1 Nomenclature for protons

Cyclodextrin carbohydrate unit:

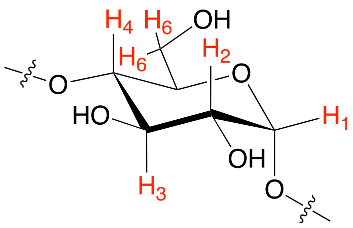

Adamantyl moiety:

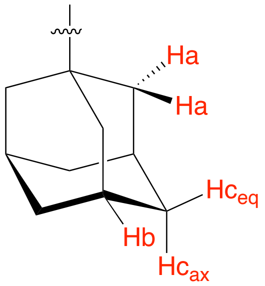

Hc_{ax} and Hc_{eq} are also labelled
Hc and Hc' when they are not assigned

SI.2.2 6^A, 6^D-Dideoxy-6^A, 6^D-azido-$2^{A\text{-}G}$, $3^{A\text{-}G}$, 6^B, 6^C, 6^E, 6^F, 6^G-enneadeca-*O*-benzyl-β-cyclodextrin (**5**)

SI.2.2.1 Synthesis and description of compound **5**

To a cooled solution (0°C) of diol β-cyclodextrin **4**[1] (4.0 g, 1.4 mmol, 1 eq) in CH_2Cl_2 (18 mL) triethylamine (0.78 mL, 5.6 mmol, 4 eq) and mesyl chloride (0.44 mL, 5.6 mmol, 4 eq) were sequentially added. The reaction mixture was stirred at room temperature under a nitrogen atmosphere, and reaction progress was monitored by TLC (cyclohexane/EtOAc 65:35). Upon completion, after 1h, the reaction was quenched with H_2O and diluted with CH_2Cl_2 (10 mL). The aqueous phase was extracted with CH_2Cl_2 (3×20 mL), and the combined organic layers were washed with water, brine, dried over $MgSO_4$, filtered and concentrated in vacuo. The residue was purified by silica gel flash chromatography (cyclohexane/EtOAc 9:1 then 3:1) to afford the desired dimesylate CD (3.7 g, 93% yield) as a white foam. The structure of the product was confirmed by comparing its NMR spectra with those reported in the literature.[1]

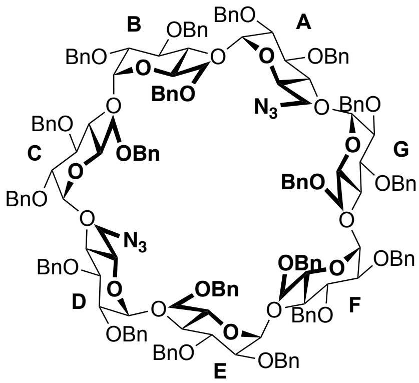

Chemical Formula: $C_{175}H_{182}N_6O_{33}$
Exact Mass: 2895,27

Dimesylate CD (3.7 g, 1.2 mmol, 1 eq) was solubilized in dry DMF (21 mL) under an argon atmosphere, and sodium azide (0.64 g, 9.9 mmol, 8 eq) was added. The reaction mixture was stirred at 80°C. The reaction progress was monitored by TLC (cyclohexane/ EtOAc 85:15). After 2h, water (50 mL) and EtOAc (50 mL) were added, the aqueous phase was extracted with EtOAc (3×70 mL), and the combined organic layers were washed with 1:1 water/brine solution, dried over $MgSO_4$, filtered and concentrated in vacuo. The obtained residue was purified by silica gel flash chromatography (cyclohexane/EtOAc 85:15) to give CD **5** (3.1 g, 87 % yield) as a white foam.

^{1}H NMR (400 MHz, $CDCl_3$, 300K): δ= 7.42-7.00 (m, 95H, H_{Ph}), 5.20 (d, $^3J_{H1\text{-}H2}$= 5 Hz, 1H, H1), 5.29-5.07 (m, 9H, 5×H1, 4×CH*H*-Ph), 5.05-4.98 (m, 2H, 1×H1, 1×CH*H*-Ph), 4.90-4.71 (m, 9H, 9×CH*H*-Ph), 4.67-4.57 (m, 2H, 2×CH*H*-Ph), 4.56-4.35 (m, 22H, 22×CH*H*-Ph), 4.20-3.84 (m, 25H, 7×H3, 5×H4, 7×H5, 6×H6), 3.76-3.41 (m, 17H, 7×H2, 2×H4, 8×H6).

^{13}C NMR (100 MHz, $CDCl_3$, 300K): δ= 139.34-138.01 (19×*C*quat-Ar), 128.51-126.77 (95C, *C*H-Ar), 98.75, 98.63, 98.58, 98.53, 98.27, 98.09, 97.92 (7×C1), 81.73-76.67 (7×C2, 7×C3, 7×C4), 75.89, 75.73, 75.65, 75.35, 75.24, 74.85 (2C) (7×CH$_2$-Ph), 73.71-72.36 (11×CH$_2$-Ph), 71.94-71.33 (5×C5), 71.06-70.61 (2×C5), 69.48 (2C), 69.28 (2C), 68.98 (2C), 52.25 (7×C6).

HMRS (ESI): calculated for $[C_{175}H_{182}N_6O_{33}+Na]^+$ 2918.2646; found 2918.2599.

[1] M. Guitet, P. Zhang, F. Marcelo, C. Tugny, J. Jiménez-Barbero, O. Buriez, C. Amatore, V. Mouriès-Mansuy, J.-P. Goddard, L. Fensterbank, Y. Zhang, S. Roland, M. Ménand, and M. Sollogoub, *Angew. Chem. Int. Ed.* **2013**, *52*, 7213-7218.

SI.2.2.2 ^{1}H and ^{13}C NMR spectra of compound **5**

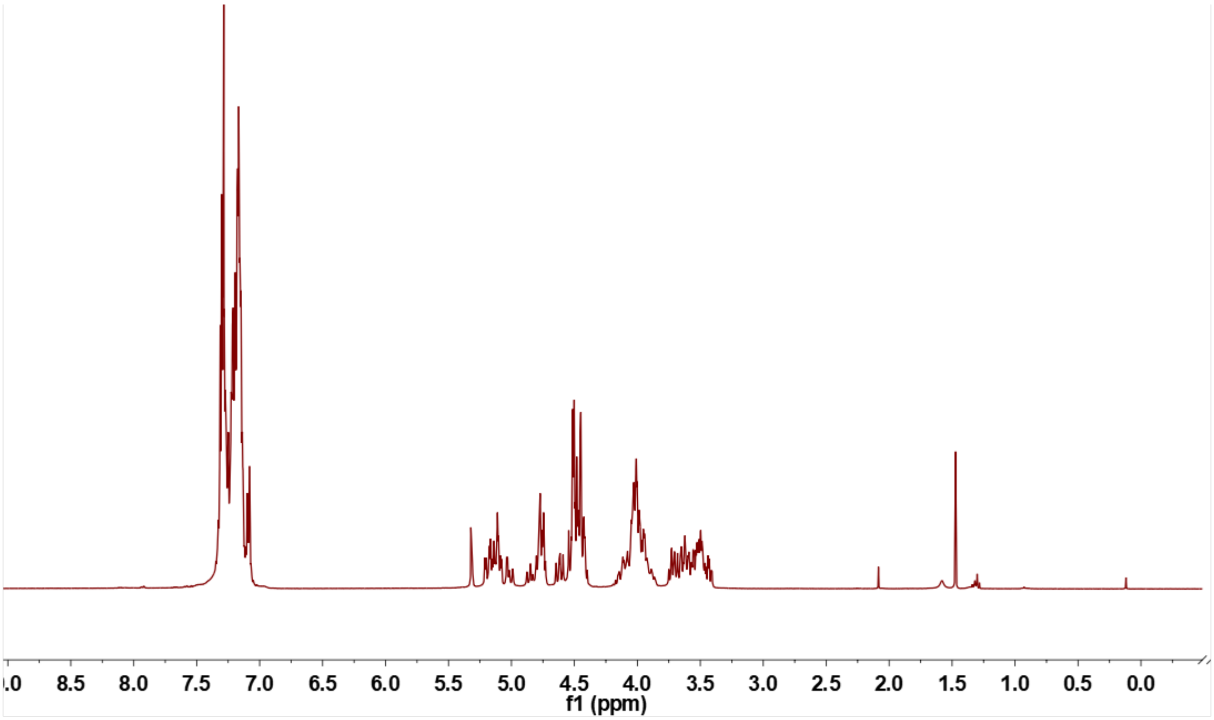

^{1}H NMR (400 MHz, $CDCl_3$, 300K) of CD **5**

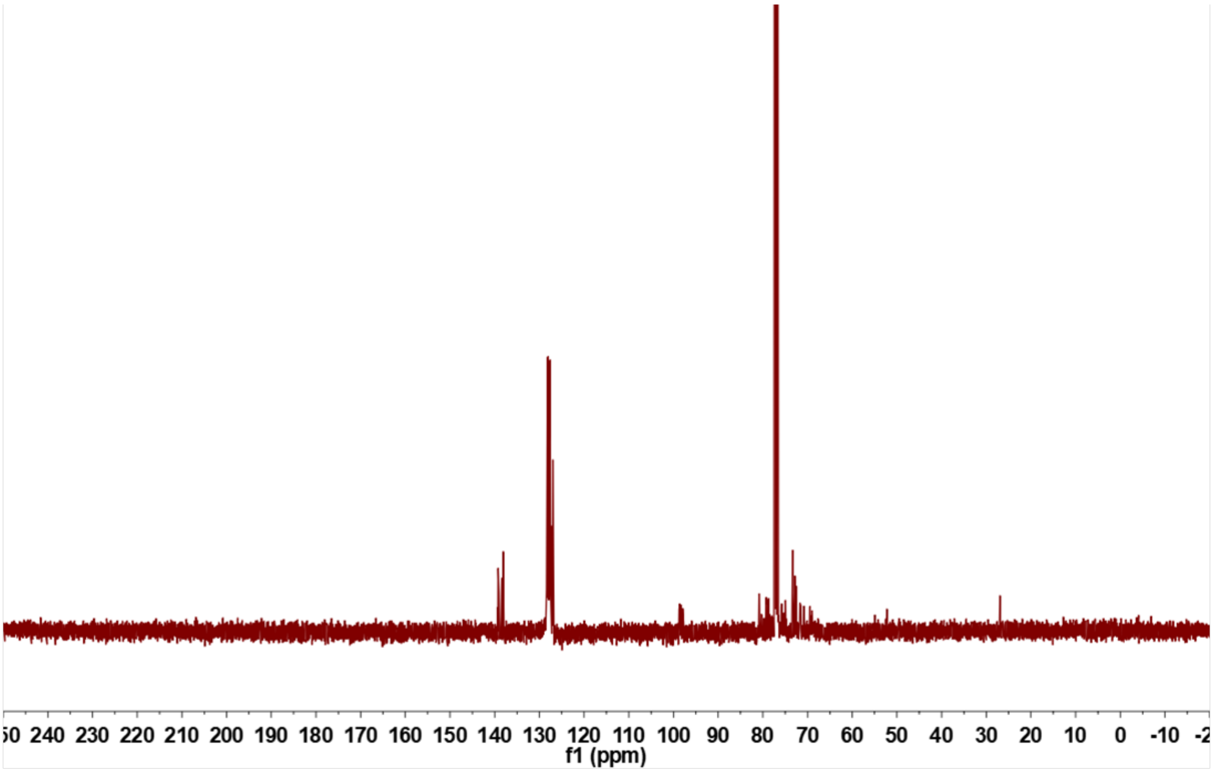

NMR ^{13}C NMR (100 MHz, $CDCl_3$, 300K) of CD **5**

SI.2.2.3 Attributions of compound **5**

	Attribution	Signal / ppm	Correlation /ppm	Attribution
Cycle A	H-1 A	5.089	3.818	$^{G}H_4$
	C-1 A	98.632		
	H-2 A	3.431		
	C-2 A	78.378		
	H-3 A	3.910		
	C-3 A	80.878		
	H-4 A	3.844		
	C-4 A	78.416		
	H-5 A	3.986		
	C-5 A	70.824		
	H-6 A	3.596, 3.969		
	C-6 A	69.276		
Cycle B	H-1 B	5.054	3.844	$^{A}H_4$
	C-1 B	98.576		
	H-2 B	3.401		
	C-2 B	78.773		
	H-3 B	3.914		
	C-3 B	79.679		
	H-4 B	3.814		
	C-4 B	78.063		
	H-5 B	3.992		
	C-5 B	70.753		
	H-6 B	3.485, 3.393		
	C-6 B	52.245		
Cycle C	H-1C	4.974	3.814	$^{B}H_4$
	C-1 C	98.267		
	H-2 C	3.374		
	C-2 C	78.812		
	H-3 C	3.885		
	C-3 C	80.325		
	H-4 C	3.813		
	C-4 C	78.063		
	H-5 C	3.988		
	C-5 C	70.753		
	H-6 C	3.889, 3.927		
	C-6 C	69.276		
Cycle D	H-1 D	4.997	3.813	$^{C}H_4$
	C-1 D	98.529		
	H-2 D	3.351		
	C-2 D	79.305		
	H-3 D	3.899		
	C-3 D	80.780		
	H-4 D	3.613		
	C-4 D	77.727		
	H-5 D	3.957		
	C-5 D	71.489		
	H-6 D	3.489, 3.517		
	C-6 D	68.981		
Cycle E	H-1 E	4.916	3.613	$^{D}H_4$
	C-1 E	97.924		
	H-2 E	3.318		
	C-2 E	79.381		
	H-3 E	3.897		
	C-3 E	80.780		
	H-4 E	3.533		
	C-4 E	78.871		
	H-5 E	3.893		
	C-5 E	71.709		
	H-6 E	3.468, 3.900		
	C-6 E	69.475		

Cycle F	H-1 F	5.205	3.533	$^{E}H_4$
	C-1 F	98.746		
	H-2 F	3.427		
	C-2 F	78.063		
	H-3 F	3.917		
	C-3 F	81.074		
	H-4 F	3.757		
	C-4 F	77.727		
	H-5 F	3.863		
	C-5 F	71.623		
	H-6 F	3.609, 4.002		
	C-6 F	69.475		
Cycle G	H-1 G	4.959	3.757	$^{F}H_4$
	C-1 G	98.085		
	H-2 G	3.371		
	C-2 G	78.888		
	H-3 G	3.877		
	C-3 G	79.853		
	H-4 G	3.818		
	C-4 G	77.982		
	H-5 G	3.974		
	C-5 G	70.824		
	H-6 G	3.511, 3.889		
	C-6 G	68.981		

SI.2.3 2-(adamantan-1-yl)acetaldehyde

SI.2.3.1 Synthesis of 2-(adamantan-1-yl)acetaldehyde

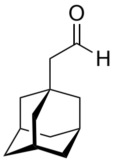

Chemical Formula: $C_{12}H_{18}O$
Exact Mass: 178,14

To a flask containing 2-adamantyl-ethanol (1.01 g, 5.60 mmol, 1 eq) in dichloromethane (35 mL) was added TEMPO (88 mg, 0.56 mmol, 0.1 eq) followed by iodobenzene diacetate (2.0 g, 6.2 mmol, 1.1 eq). The reaction mixture was stirred for 3h and then diluted with dichloromethane (20 mL). A saturated aqueous solution of $Na_2S_2O_3$ (20 mL) was then added. The mixture was stirred for 30 min, the aqueous phase was then extracted with dichloromethane (3×50 mL), and the combined organic layers were washed with a saturated aqueous solution of $NaHCO_3$, brine, dried over $MgSO_4$, filtered and concentrated in vacuo. The residue was purified by silica gel flash chromatography (Et_2O/Pentane 1:30) to provide adamantylacetaldehyde **6** (0.77 g, 77% yield) as a colorless oil that was stored at -20°C under argon to prevent any further oxidation.

The structure of the product was confirmed by comparing its NMR spectra with those reported in the literature.[2]

SI.2.4 2-(adamantan-1-yl)-N,N-dipropargylethanamine (**6**)

SI.2.4.1 Synthesis and description of compound **6**

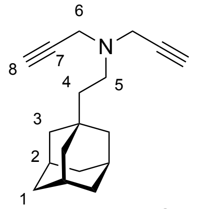

Chemical Formula: $C_{18}H_{25}N$
Exact Mass: 255,20

To a solution of adamantylacetaldehyde (770 mg, 4.32 mmol, 1 eq) in dry THF (24 mL) was added dipropargylamine (0.45 mL, 4.3 mmol, 1 eq) followed by $NaBH(OAc)_3$ (1.37 g, 6.48 mmol, 1.5 eq) at room temperature under nitrogen, and the mixture was stirred for 2h. Ethyl acetate (30 mL) and a saturated aqueous solution of $NaHCO_3$ (30 mL) were then added. The mixture was stirred for 30 min, after which the aqueous phase was extracted with ethyl acetate (3×50 mL), and the combined organic layers were washed with a saturated aqueous solution of $NaHCO_3$, brine, dried over $MgSO_4$, filtered and concentrated in vacuo. The residue was purified by silica gel flash chromatography (cyclohexane/EtOAc 5:1) to afford the desired dialkyne product **6** (946 mg, 86% yield) as pale yellow oil.

^{1}H NMR (400 MHz, $CDCl_3$, 300K) : δ= 3.44 (s, 4H, 4H-6), 2.55 (m, 2H, 2H-5), 2.21 (t, *J* = 2 Hz, 2H, 2H-8), 1.94 (s, 3H, 3H-2), 1.73-1.60 (m, 6H, 6H-1), 1.51 (d, *J* = 2 Hz, 6H, 6H-3), 1.30-1.23 (m, 2H, 2H-4).

^{13}C NMR (100 MHz, $CDCl_3$, 300K) : δ= 78.85 (C-7), 72.84 (C-8), 47.38 (C-5), 42.27 (C-3), 42.08 (C-6), 41.54 (C-4), 37.12 (C-1) 31.84 (Cquat-Ad), 28.65 (C-2).

HMRS (ESI): calculated for $[C_{18}H_{25}N +H]^+$ 256.2060; found 256.2077.

[2] T.D. Beeson, D.W.C. MacMillan, *J. Am. Chem. Soc.* **2005**, *127*, 8826-8828.

SI.2.4.2 ^{1}H and ^{13}C NMR spectra of compound **6**

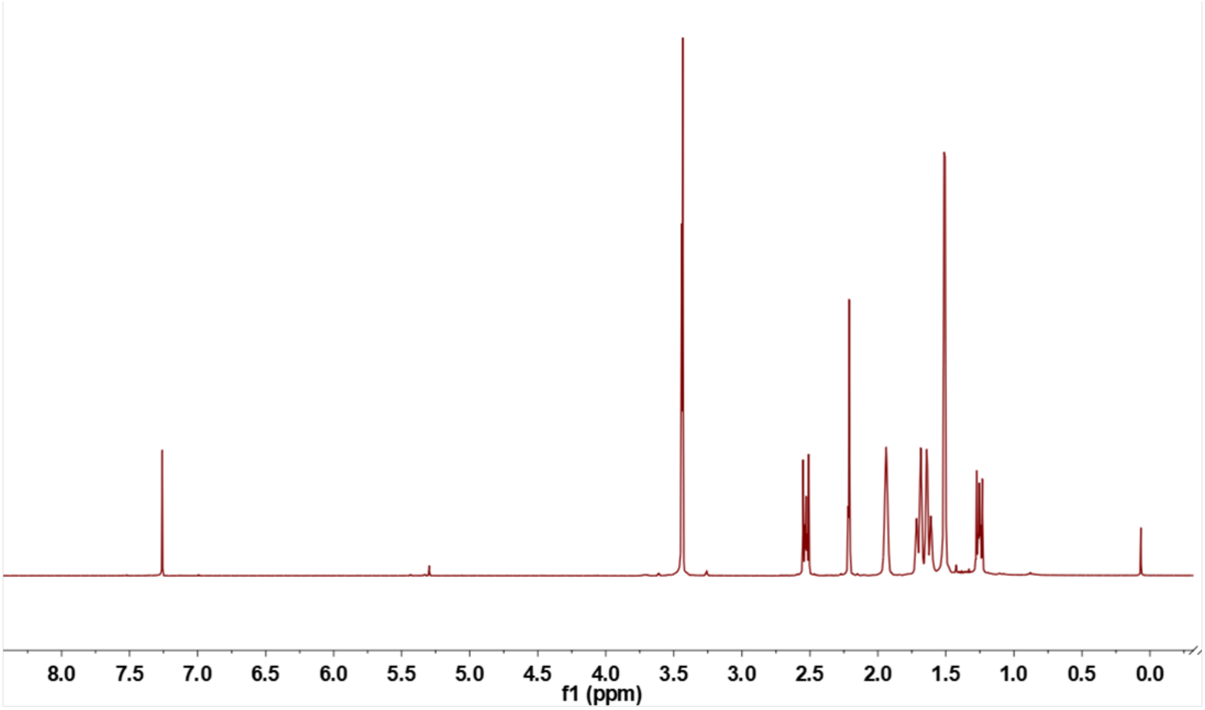

^{1}H NMR (400 MHz, $CDCl_3$, 300K) of compound **6**

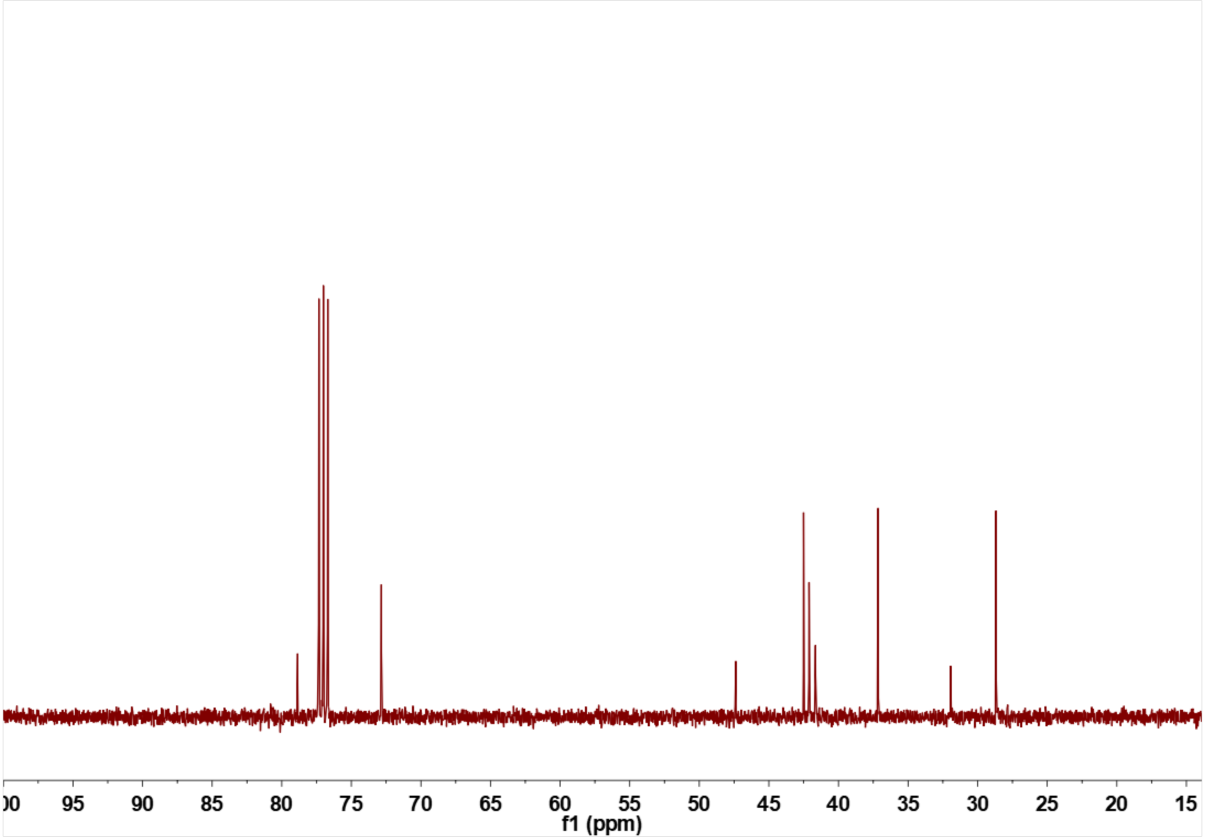

^{13}C NMR (100 MHz, $CDCl_3$, 300K) of compound **6**

SI.2.5 6^A, 6^D-Dideoxy-6^A, 6^D-ditriazole-bridged-*N*-ethyladamantyl-$2^{A\text{-}G}$, $3^{A\text{-}G}$, 6^B, 6^C, 6^E, 6^F, 6^G-enneadeca-O-benzyl-β-cyclodextrin (**7**)

SI.2.5.1 Synthesis and description of compound **7**

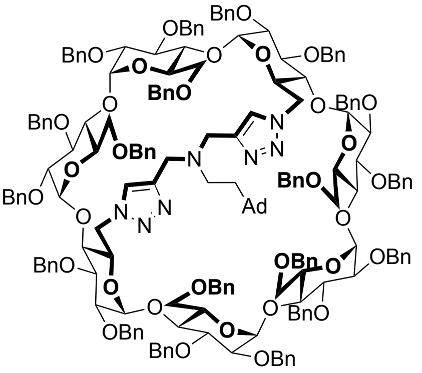

Chemical Formula: $C_{193}H_{207}N_7O_{33}$
Exact Mass: 3150,47

To a solution of CD **5** (1.0 g, 0.34 mmol, 1 eq) in 12 mL DMF, dialkyne **6** (88 mg, 0.34 mmol, 1 eq) and DIPEA (24 µL, 0.14 mmol, 0.4 eq) were added. A mixture of TBTA (73 mg, 0.14 mmol, 0.4 eq) and $Cu(CH_3CN)_4PF_6$ (51 mg, 0.14 mmol, 0.4 eq) in 1 mL DMF were next added to the solution. The reaction mixture was stirred at 150°C under an argon atmosphere for 1h. The mixture was then diluted with Et_2O (100 mL). The organic solution was washed with a 1:1 water/brine solution (2×100 mL), an HCl solution (1 M in water, 100 mL), a saturated aqueous solution of $NaHCO_3$ (100 mL), then dried over $MgSO_4$, filtered and concentrated in vacuo. The residue was purified by silica gel flash chromatography (cyclohexane/EtOAc 7:3) to afford bridged CD **7** (381 mg, 35% yield) as a white foam.

^{1}H NMR (400 MHz, $CDCl_3$, 300K): δ= 7.70 (s, 1H, H_{triazA}), 7.62 (s, 1H, H_{triazD}), 7.29-7.08 (m, 95H, H_{Ph}), 5.37-5.19 (m, 6H, 2×H1, 1×H6, 3×CH*H*Ph), 5.12-5.08 (m, 3H, 2×H1, 1×CH*H*Ph), 5.01-4.98 (m, 2×CH*H*Ph), 4.90-4.70 (m, 3×H1, 11×CH*H*Ph), 4.64-4.55 (m, 4H, 1×H5, 3×CH*H*Ph), 4.52-4.30 (m, 17H, 1×H5, 16×CH*H*Ph), 4.28-4.14 (m, 8H, 2×H3, 2×H5, 3×H6, 1×CH*H*Ph), 4.09-3.94 (m, 7H, 5×H3, 1×H4, 1×CH*H*Ph), 3.87-3.65 (m, 12H, 4×H4, 3×H5, 5×H6), 3.56-3.34 (m, 11H, 7×H2, 2×H4, 2×CHH_{triaz}), 3.31-3.28 (m, 2H, 1×H6, 1×CHH_{triaz}), 3.13-3.06 (m, 3H, 2×H6, 1×CHH_{triaz}), 2.81 (m, 2H, 2×H6), 2.51 (td, ^2J = 12 Hz, ^3J= 5 Hz, 1H, 1×N-CH*H*-CH_2-Ad), 2.17 (td, ^2J = 12 Hz, ^3J= 5 Hz, 1H, 1×N-CH*H*-CH_2-Ad), 1.97 (s, 3H, Hb), 1.76-1.62 (m, 9H, 6×Hc, 2×Ha), 1.47 (m, 1H, 1×N-CH_2-CH*H*-Ad), 1.34-1.24 (m, 5H, 4×Ha, 1×N-CH_2-CH*H*-Ad).

^{13}C NMR (100 MHz, $CDCl_3$, 300K): δ= 128.32-126.61 (95C, *C*H-Ar), 126.12 (*C*triazA), 125.90 (*C*triazD), 101.70, 101.13, 100.77, 99.87, 99.61, 99.35, 99.10 (7×C1), 82.49 (C2), 82.08 (C4), 81.53 (C4), 81.26 (C4), 80.98 (C3), 80.45 (C3), 80.28 (C3), 80.20 (C3,C4), 79.98 (C3), 79.85 (2×C3), 79.53 (C2), 79.44 (C2), 79.37 (C2), 79.13 (C2,C4), 77.85 (C2), 77.47 (C2), 77.22 (C2), 76.25 (2C), 75.90, 75.83, 75.43, 75.22, 74.56, 74.13, 73.40, 73.35, 73.19, 73.08, 72.84, 72.80, 72.71 (2C), 72.41, 72.21, 72.08 (19×CH_2-Ph), 71.77 (C5), 71.65 (C5), 71.40 (2×C5), 71.38 (C4), 71.26 (2×C5), 70.46 (C5), 69.88, 69.24, 68.85, 67.67, 67.42, 53.04, 52.61 (7×C6), 48.46 (N-*C*H_2-CH_2-Ad), 45.67 (*C*H_2triazA), 44.82 (*C*H_2triazD), 42.63 (Ca), 42.27 (N-CH_2-*C*H_2-Ad), 37.19 (Cc), 31.83 (*C*quat-Ad), 28.72 (Cb).

HMRS (ESI): calculated for $[C_{193}H_{207}N_7O_{33} + H]^+$ 3151.4808; found 3151.4978.

SI.2.5.2 ^{1}H and ^{13}C NMR spectra of compound **7**

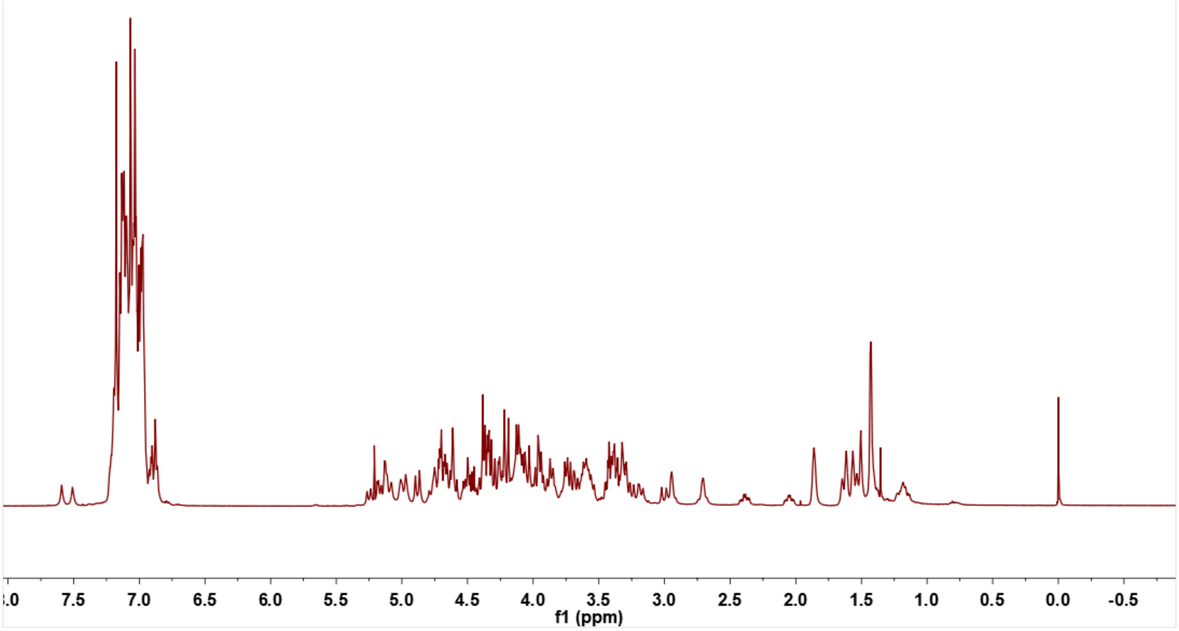

^{1}H NMR (400 MHz, $CDCl_3$, 300K) of CD **7**

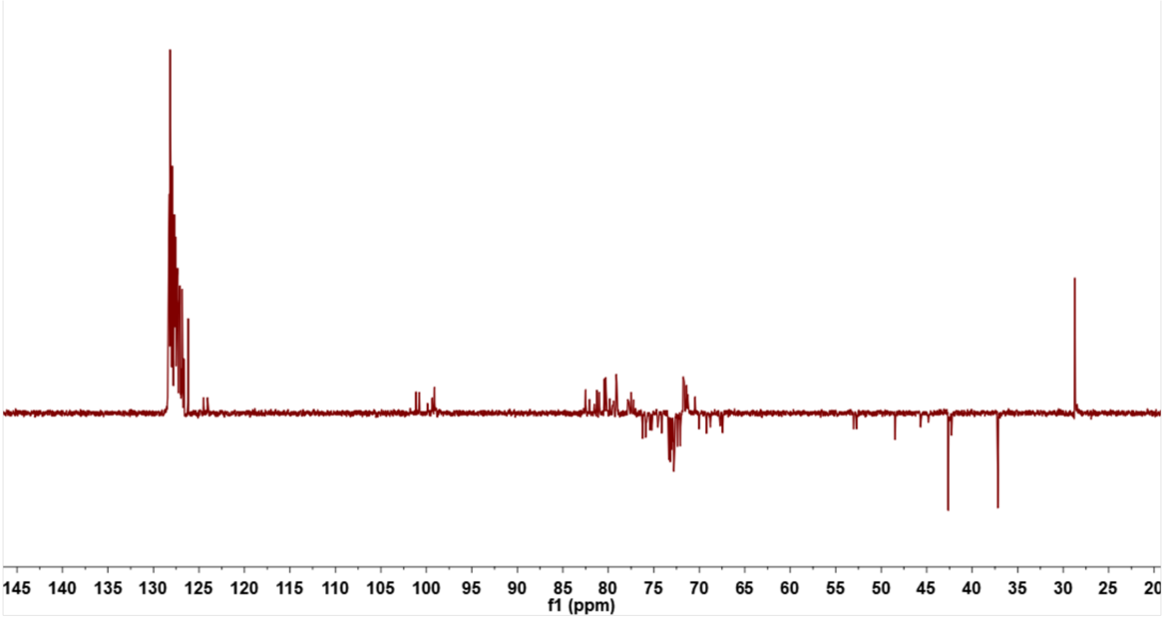

^{13}C NMR-DEPT 135 (100 MHz, $CDCl_3$, 300K) of CD **7**

SI.2.5.3 Attributions of protons and carbons of compound **7**

	Attribution	Signal / ppm	Correlation / ppm	Attribution
Cycle A	H-1A	5.133	3.719	$^{G}H_4$
	C-1 A	98.527		
	H-2 A	3,379		
	C-2 A	77.694		
	H-3 A	3,963		
	C-3 A	79.583		
	H-4 A	3.887		
	C-4 A	80.245		
	H-5 A	3.739		
	C-5 A	71.670		
	H-6 A	3.679, 3.173		
	C-6 A	68.846		
Cycle B	H-1 B	5.011	3.887	$^{A}H_4$
	C-1 B	98.939		
	H-2 B	3.317		
	C-2 B	79.065		
	H-3 B	3.853		
	C-3 B	80.468		
	H-4 B	3.686		
	C-4 B	81.241		
	H-5 B	3.601		
	C-5 B	71.753		
	H-6 B	2.949		
	C-6 B	67.674		
Cycle D	H-1 D	4.965	3.403	$^{C}H_4$
	C-1 D	99.831		
	H-2 D	3.445		
	C-2 D	77.846		
	H-3 D	4.056		
	C-3 D	80.245		
	H-4 D	3.757		
	C-4 D	77.734		
	H-5 D	4.134		
	C-5 D	71.249		
	H-6 D	3.746, 3.621		
	C-6 D	69.879		
Cycle C	H-1C	4.749	3.686	$^{B}H_4$
	C-1 C	99.351		
	H-2 C	3.280		
	C-2 C	79.838		
	H-3 C	3.966		
	C-3 C	79.432		
	H-4 C	3.403		
	C-4 C	81.496		
	H-5 C	4.403		
	C-5 C	71.249		
	H-6 C	4.135, 4.156		
	C-6 C	53.037		
Cycle G	H-1 G	4.716	3.621	$^{F}H_4$
	C-1 G	99.085		
	H-2 G	3.312		
	C-2 G	79.065		
	H-3 G	3.874		
	C-3 G	80.205		
	H-4 G	3.719		
	C-4 G	80.906		
	H-5 G	3.554		
	C-5 G	71.670		
	H-6 G	2.707		
	C-6 G	67.423		

Cycle E	H-1 E	4.702	3.757	$^{D}H_4$
	C-1 E	101.114		
	H-2 E	3.332		
	C-2 E	79.137		
	H-3 E	3.965		
	C-3 E	79.583		
	H-4 E	3.392		
	C-4 E	82.532		
	H-5 E	4.527		
	C-5 E	70.447		
	H-6 E	4.115, 5.105		
	C-6 E	52.614		
Cycle F	H-1 F	5.185	3.392	$^{E}H_4$
	C-1 F	100.749		
	H-2 F	3.419		
	C-2 F	77.184		
	H-3 F	4.105		
	C-3 F	80.468		
	H-4 F	3.621		
	C-4 F	82.126		
	H-5 F	4.050		
	C-5 F	71.410		
	H-6 F	3.763, 3.646		
	C-6 F	69.237		

SI.2.5.4 NOESY spectrum of compound **7**

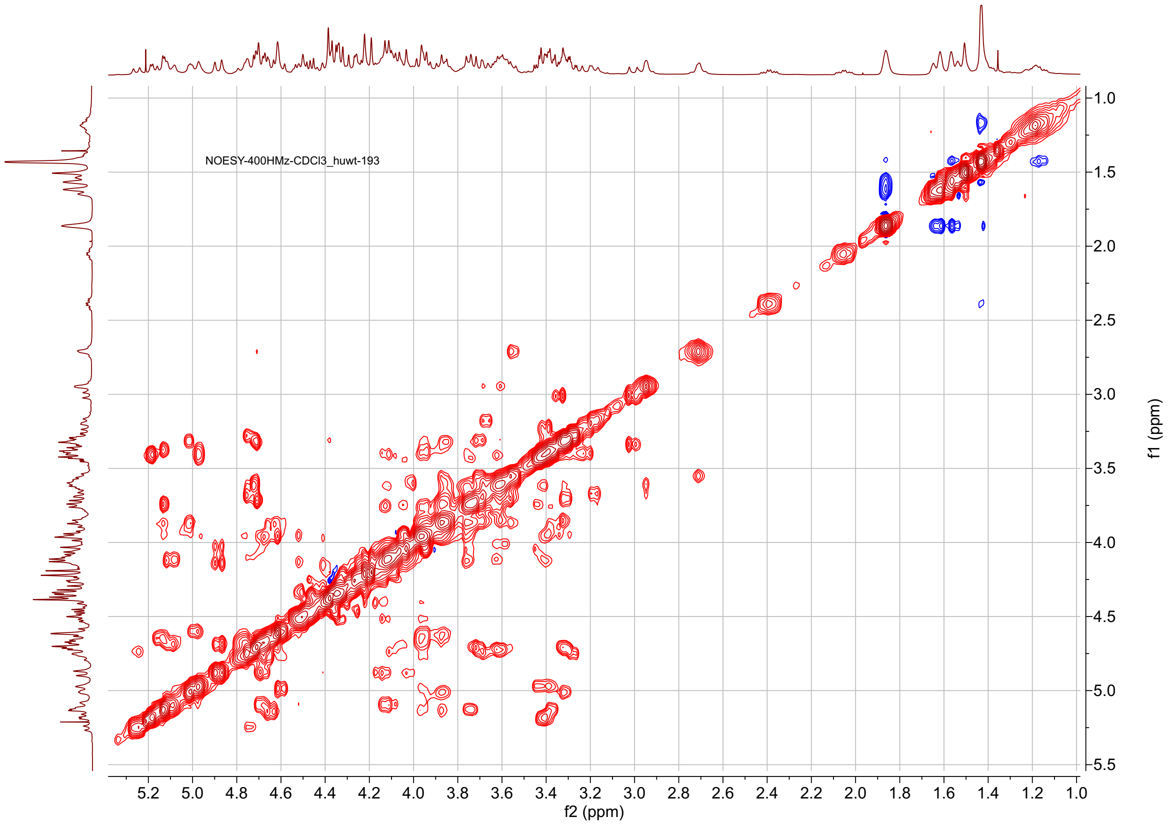

Figure S 1. NOESY spectrum of CD **7** showing no cross-correlations between adamantyl protons and CD cavity protons.

SI.2.6 6^A, 6^D-Dideoxy-6^A, 6^D-di-triazole-bridged-*N*-ethyladamantyl-β-cyclodextrin trifluoroacetate (**3-out•H⁺**)

SI.2.6.1 Synthesis and description of compound **3-out•H⁺**

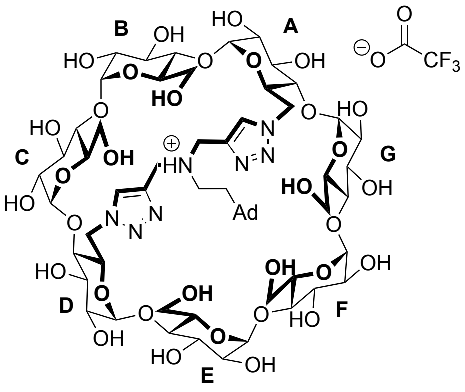

Chemical Formula: $C_{60}H_{94}N_7O_{33}^+$
Exact Mass: 1440,59

Perbenzylated CD **7** (200 mg, 0.06 mmol, 1 eq) was dissolved in THF/H_2O (3:1, 40 mL). 2,2,2-trifluoroacetic acid (39 µL, 0.51 mmol, 8 eq) and Pd/C (290 mg, 2.73 mmol, 43 eq) were then added to this solution. The mixture was purged 3 times with argon and 3 times with hydrogen. The reaction mixture was monitored by MS, upon completion, the reaction mixture was purged with nitrogen, filtered through a µ-filter (0.2 µm-polyester) (with no dilution). The organic solvent was evaporated under vacuum and the residue was lyophilized. The crude product was purified on a RediSep Rf Gold C-18 reversed-phase column chromatography using a gradient of CH_3CN/water (%CH_3CN: 0→5% in 10 min) to afford the deprotected CD **3-out•H⁺** (54 mg, 58% yield) as a white amorphous powder.

^{1}H NMR (600 MHz, D_2O, 300K): δ= 8.67-8.21 (br. m, 2H, 2×H_{triaz}), 5.26-5.15 (m, 2H, 2×H1), 5.15-4.85 (m, 7H, 5×H1, 2×H6), 4.84-4.60 (m, 2H, 2×H6), 4.00-3.25 (m, 38H, 7×H2, 7×H3, 7×H4, 7×H5, 10×H6), 3.23-2.85 (m, 6H, 4×CHH_{triaz}, 2×N-CH*H*-CH_2-Ad), 2.16 (s, 3H, Hb), 1.90 (br. m, 3H, Hc), 1.77 (br. m, 3H, Hc), 1.71-1.47 (br. m, 8H, Ha, 2×N-CH_2-CH*H*-Ad).

^{13}C NMR (151 MHz, D_2O, 300K): δ= 128.41 (*C*triaz), 127.76 (*C*triaz), 102.51 (C1), 102.40 (C1), 102.22 (C1), 102.00 (C1), 101.71 (C1), 100.89 (2×C1), 83.53, 83.19, 82.29, 81.98, 81.49, 81.25, 80.18 (7×C4), 74.36-71.49 (7×C2, 7×C3, 7×C5), 61.70, 61.54, 60.75, 60.52, 60.16 (5×C6), 59.09, 59.04 (2C, 2×*C*H_2triaz), 51.83 (2×C6), 49.22 (N-*C*H_2-CH_2-Ad), 41.76 (Ca), 36.98 (Cc), 28.43 (Cb), 27.87 (N-CH_2-*C*H_2-Ad).

HMRS (ESI): calculated for $[C_{60}H_{93}N_7O_{33}+H]^+$ 1440.5887 ; found 1440.5890.

SI.2.6.2 ^{1}H and ^{13}C NMR spectra of compound **3-out•H⁺**

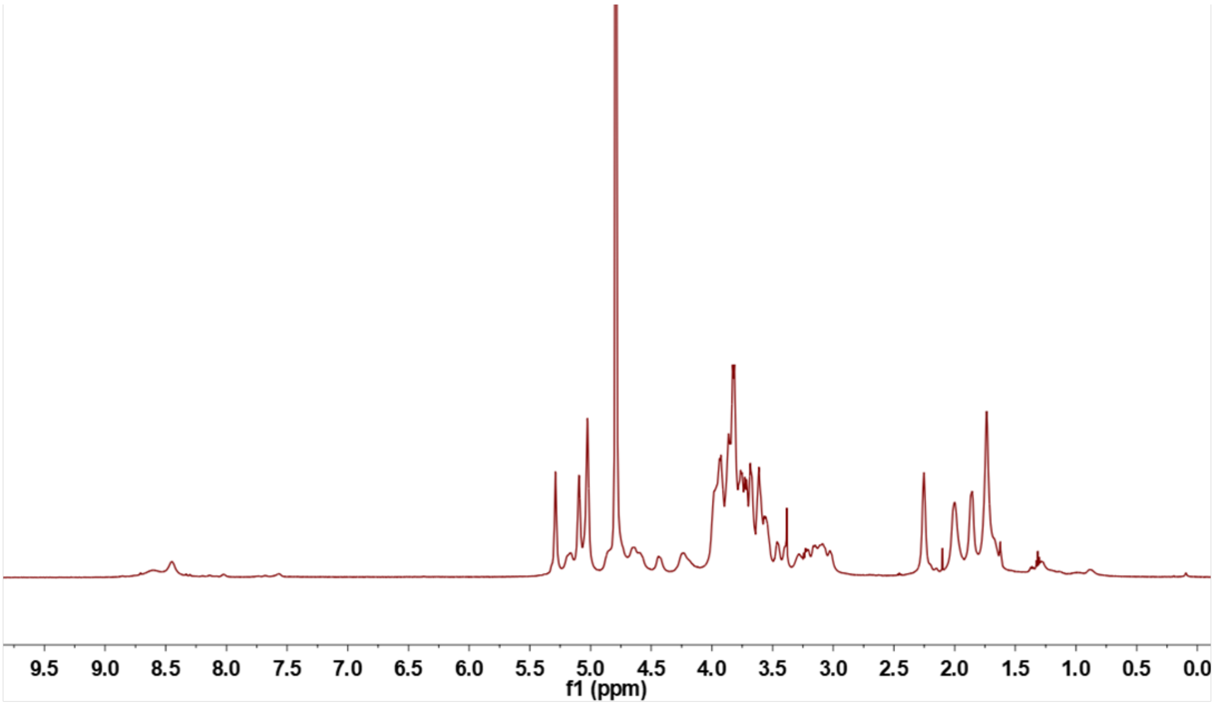

^{1}H NMR (600 MHz, D_2O, 300K) of CD **3-out·H⁺**

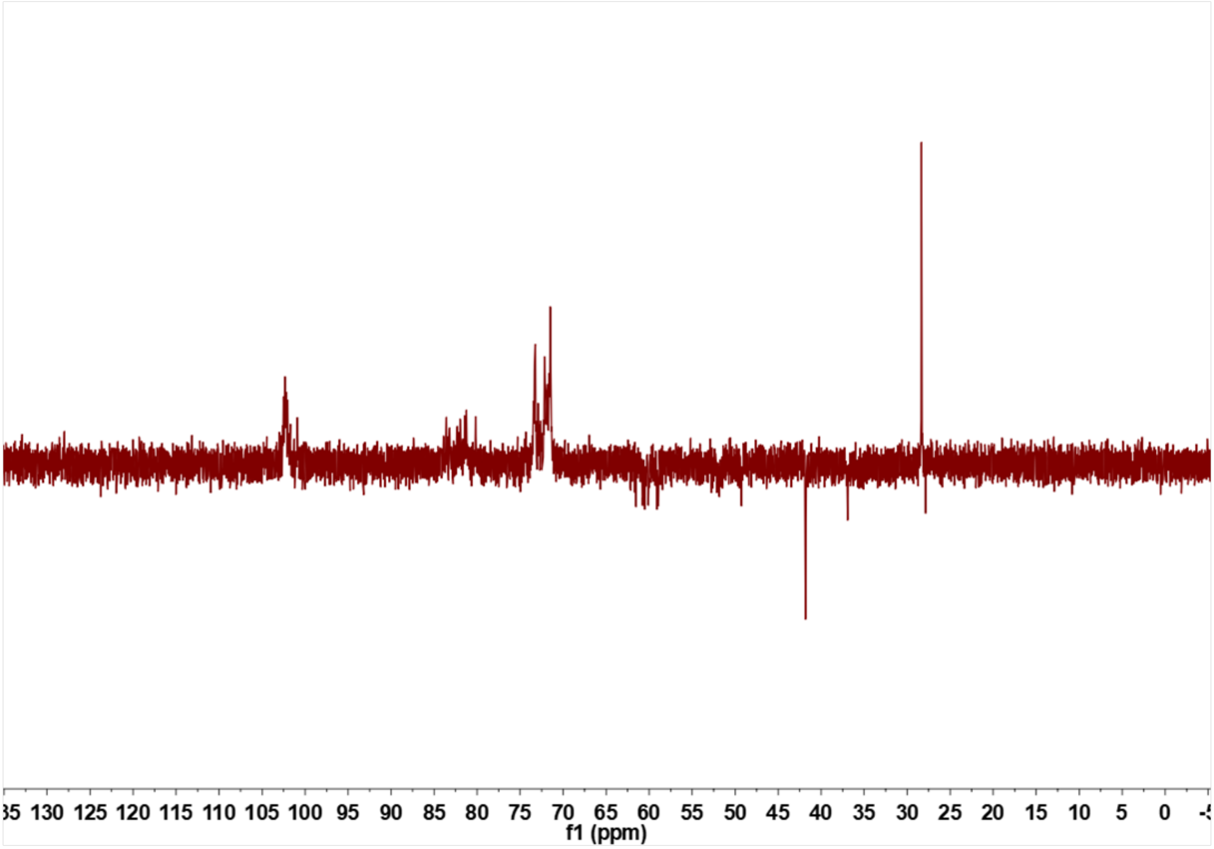

^{13}C NMR-DEPT 135 (151 MHz, D_2O, 300K) of CD **3-out·H⁺**

SI.2.7 6^{A}, 6^{D}-Dideoxy-6^{A}, 6^{D}-di-triazole-bridged-*N*-ethyladamantyl-β-cyclodextrin (**3-in**)

SI.2.7.1 Synthesis and description of compound **3-in**

CD **3-out•H^{+}** was neutralized with a saturated aqueous solution of $NaHCO_3$. The salts were removed by dialysis (100/500 molecular weight cutoff) to give **3-in** as a white amorphous powder after freeze drying.

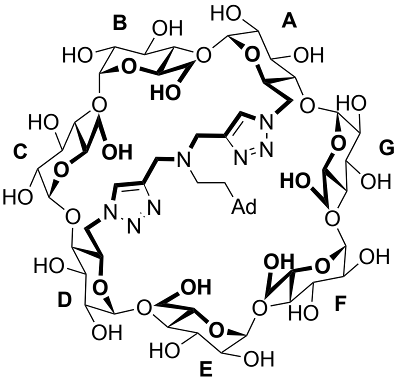

Chemical Formula: $C_{60}H_{93}N_7O_{33}$
Exact Mass: 1439,58

^{1}H NMR (600 MHz, D_2O, 300K): δ= 8.29 (s, 1H, H_{triazA}), 7.85 (s, 1H, H_{triazD}), 5.31 (d, 2H, 2×H1), 5.24-4.98 (m, 7H, 5×H1, 2×H6), 4.76 (m, 2H, 2×H6), 4.37-4.14 (m, 4H, 4×H6), 4.06-3.51 (m, 34H, 7×H2, 7×H3, 7×H4, 6×H5, 4×H6, 4×CHH_{triaz}), 3.38 (d_b, J = 10 Hz, H5), 3.28 (d_b, J = 12 Hz, 1H, H6), 3.00 (d, $^{3}J_{H6-H5}$ = 8 Hz, 1H, 1×H6), 2.46 (br. m, 2×N-CH*H*-CH_2-Ad), 2.15 (s, 3H, Hb), 1.89 (br. m, 3H, Hc), 1.71 (br. m, 3H, Hc), 1.33 (br. m, 6H, Ha), 0.83 (br. m, 1H, 1×N-CH_2-CH*H*-Ad), 0.05 (br. m, 1H, 1×N-CH_2-CH*H*-Ad).

^{13}C NMR (151 MHz, D_2O, 300K): δ=128.98 (*C*triazA), 123.21 (*C*triazD), 102.79 (2×C1), 102.58 (C1), 102.55 (C1), 102.44 (2×C1), 102.08 (C1), 83.62 (C4), 83.48 (C4), 82.73 (C4), 82.16 (C4), 81.69 (C4), 81.55 (C4), 81.44 (C4), 74.98-71.49 (7×C2, 7×C3, 7×C5), 61.21 (C6), 60.83 (C6), 60.08 (2C, 2×*C*H_2triaz), 58.85 (C6), 53.87 (C6), 52.14 (C6), 51.48 (C6), 51.42 (C6), 47.77 (N-*C*H_2-CH_2-Ad), 45.15 (N-CH_2-*C*H_2-Ad), 43.17 (Ca), 36.07 (Cc), 27.61 (Cb).

HMRS (ESI): calculated for $[C_{60}H_{93}N_7O_{33}+H]^{+}$ 1440.5887 ; found 1440.5890.

SI.2.7.2 ^{1}H and ^{13}C NMR spectra of compound **3-in**

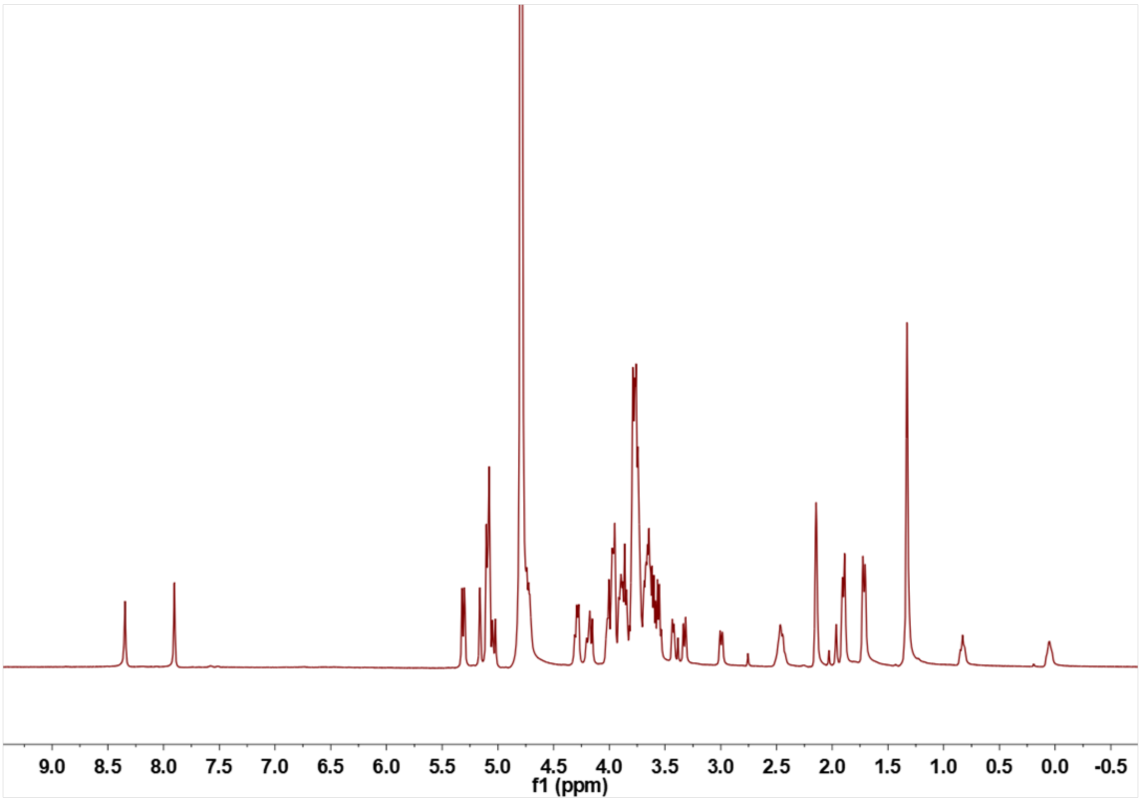

^{1}H NMR (600 MHz, D_2O, 300K) of CD **3-in**

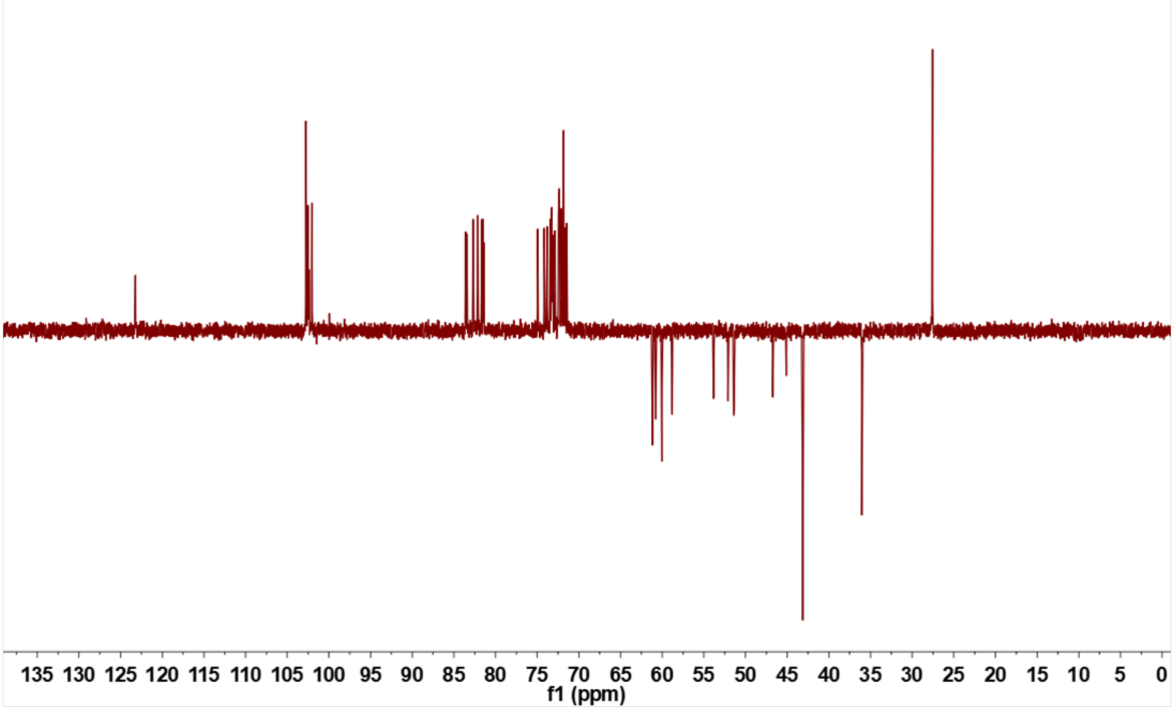

^{13}C NMR-DEPT 135 (151 MHz, D_2O, 300K) of CD **3-in**

SI.2.8 6^A, 6^D-Dideoxy-6^A, 6^D-di-triazole-bridged-*N*-ethyladamantyl-β-cyclodextrin trifluoroacetate (**3-in•H$^+$**)

SI.2.8.1 Synthesis and description of compound **3-in•H$^+$**

Protonated self-included derivative **3-in•H$^+$** was obtained by addition of 3 equivalents of 2,2,2-trifluoroacetic acid (TFA) to **3-in**.

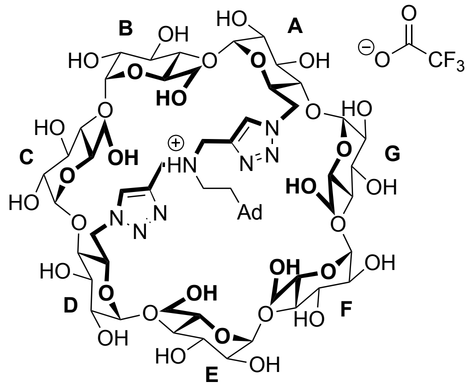

Chemical Formula: $C_{60}H_{94}N_7O_{33}^+$
Exact Mass: 1440,59

^{1}H NMR (600 MHz, D_2O, 300K): δ= 8.74 (s, 1H, H_{triazA}), 8.37 (s, 1H, H_{triazD}), 5.33 (d, $^3J_{H1-H2}$ = 6 Hz, 2H, 2×H1), 5.16 (m, 2H, 2×H1), 5.13-5.07 (m, 4H, 3×H1, H6), 5.06-4.99 (m, 2H, 2×H6), 4.92 (d, $^2J_{H6-H6'}$= 15 Hz, 1H, H6), 4.76 (m, 2H, 2×H6), 4.66 (d, $^2J_{H6-H6'}$= 12 Hz, 1H, 1×H6), 4.28 (d, $^2J_{H6-H6'}$= 12 Hz, 1H, 1×H6), 4.12 (d, $^2J_{H6-H6'}$= 10 Hz, 1H, 1×H6), 4.06-4.00 (m, 2H, 2×H6), 4.00-3.95 (m, 1H, 1×H3), 3.94-3.64 (m, 26H, 6×H2, 6×H3, 7×H5, 3×H6, 4×CHH_{triaz}), 3.64-3.50 (m, 8H, 7×H4, 1×H2), 3.29 (br. m, 1×N-CH*H*-CH_2-Ad), 2.94 (br. m, 1×N-CH*H*-CH_2-Ad), 2.21 (s, 3H, Hb), 1.91 (br. m, 3H, Hc), 1.72 (br. m, 3H, Hc), 1.26 (br. m, 6H, Ha), 0.89 (br. m, 1H, 1×N-CH_2-CH*H*-Ad), 0.10 (br. m, 1H, 1×N-CH_2-CH*H*-Ad).

^{13}C NMR (151 MHz, D_2O, 300K): δ= 128.56 (*C*triazA), 124.34 (*C*triazD), 102.74 (2×C1), 102.50 (C1), 102.16 (2×C1), 102.10 (C1), 83.71 (C3), 83.47 (C3), 82.74 (2×C4), 82.45 (2×C4), 81.98 (C4), 81.90 (C2), 81.65 (C4), 74.82 (C5), 73.75-71.42 (7×C2, 6×C3, 6×C5), 61.45 (C6), 61.24 (C6), 60.55 (C6), 60.46 (C6), 59.77 (2C, 2×*C*H_2triaz), 52.41 (C6), 51.92 (C6), 51.57 (C6), 47.74 (N-*C*H_2-CH_2-Ad), 42.76 (Ca), 42.10 (N-CH_2-*C*H_2-Ad), 35.93 (Cc), 27.49 (Cb).

HMRS (ESI): calculated for $[C_{60}H_{93}N_7O_{33}+H]^+$ 1440.5887 ; found 1440.5891.

SI.2.8.2 ^{1}H and ^{13}C NMR spectra of compound **3-in•H$^+$**

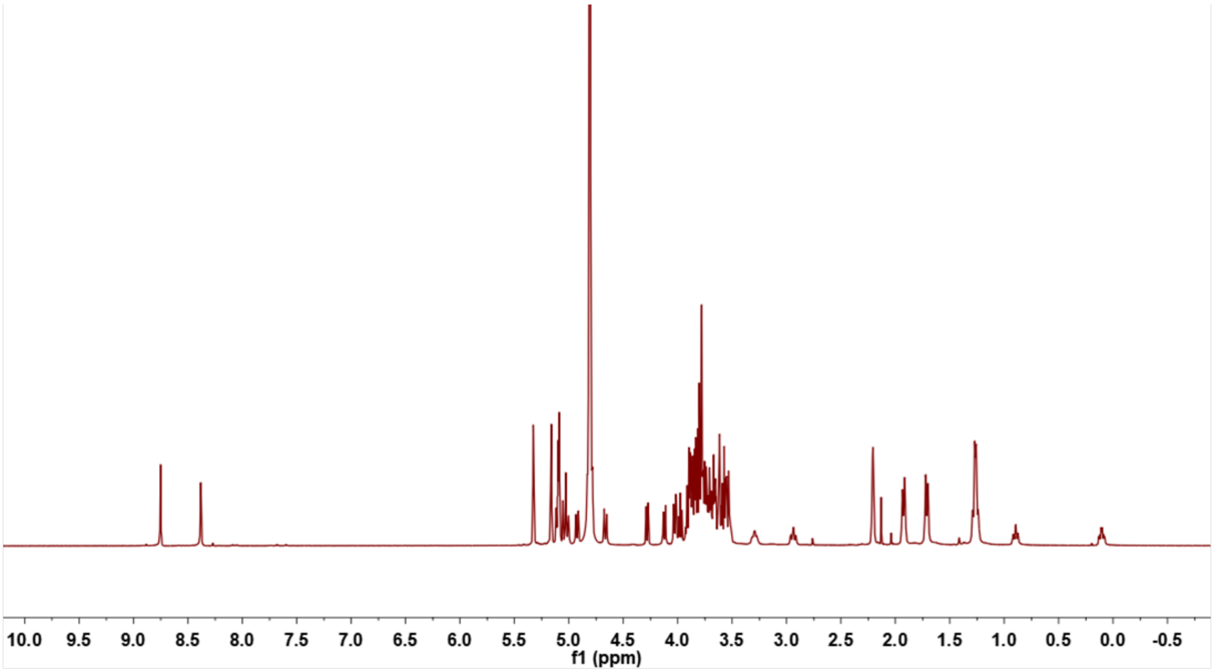

^{1}H NMR (600 MHz, D_2O, 300K) of CD **3-in·H$^+$**

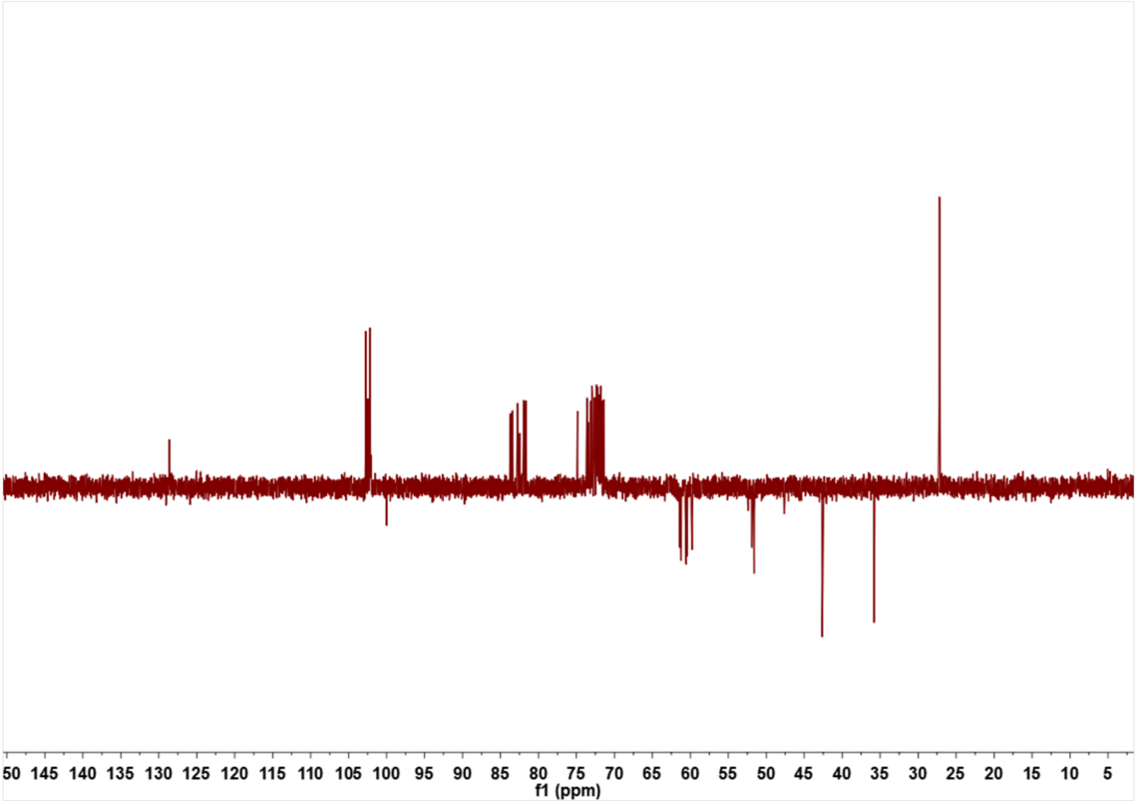

^{13}C NMR-DEPT 135 (151 MHz, D_2O, 300K) of CD **3-in·H$^+$**

SI.3 ¹H NMR titration of **3-in** with TFA in D_2O

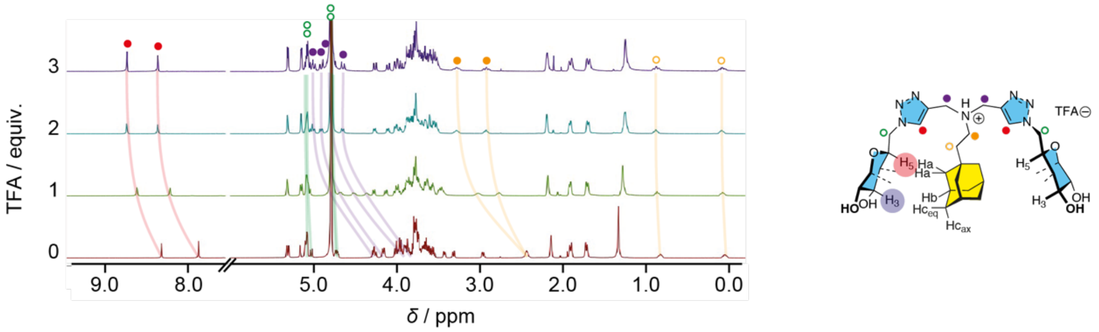

Figure S 2. ¹H NMR (D_2O, 600 MHz, 300 K) spectra of CD **3-in** (8 mM) upon addition of TFA giving **3-in·H⁺**

SI.4 ¹H NMR spectra of **3-in•H⁺** and **3-in•H⁺** in D_2O at variable temperatures

SI.4.1 ¹H NMR spectra of **3-in•H⁺** in D_2O at variable temperatures

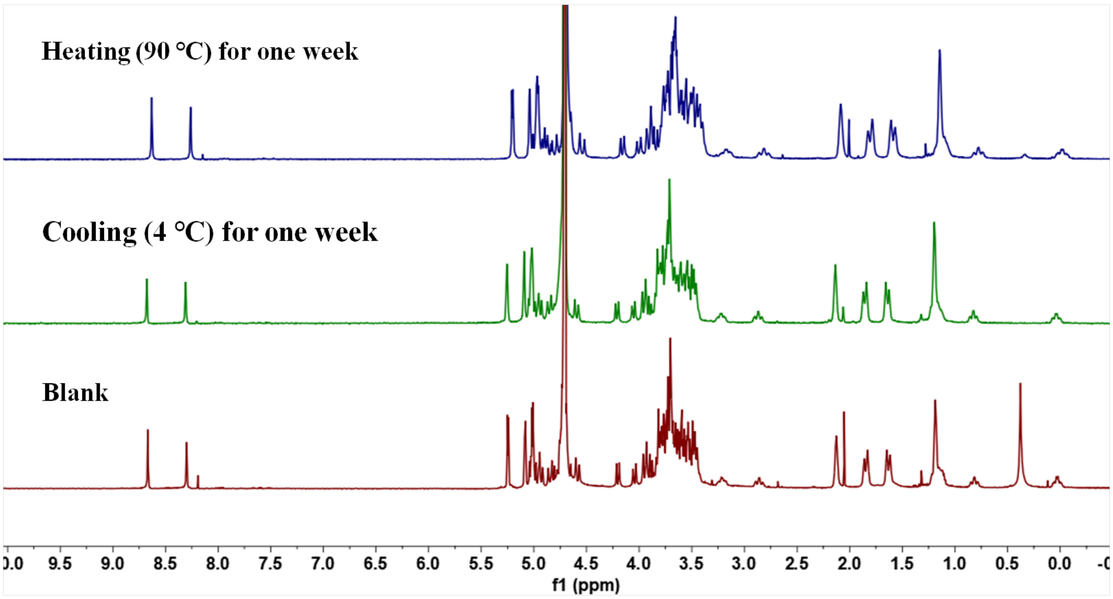

Figure S 3. ¹H-NMR (D_2O, 600 MHz) of a solution of compound **3-in·H⁺** at 8 mM at 300 K (bottom), the same solution cooled at 277 K for one week (middle), and heated to 363 K for one week (top).

SI.4.2 ^{1}H NMR spectra of **3-out•H⁺** in D_2O at variable temperatures

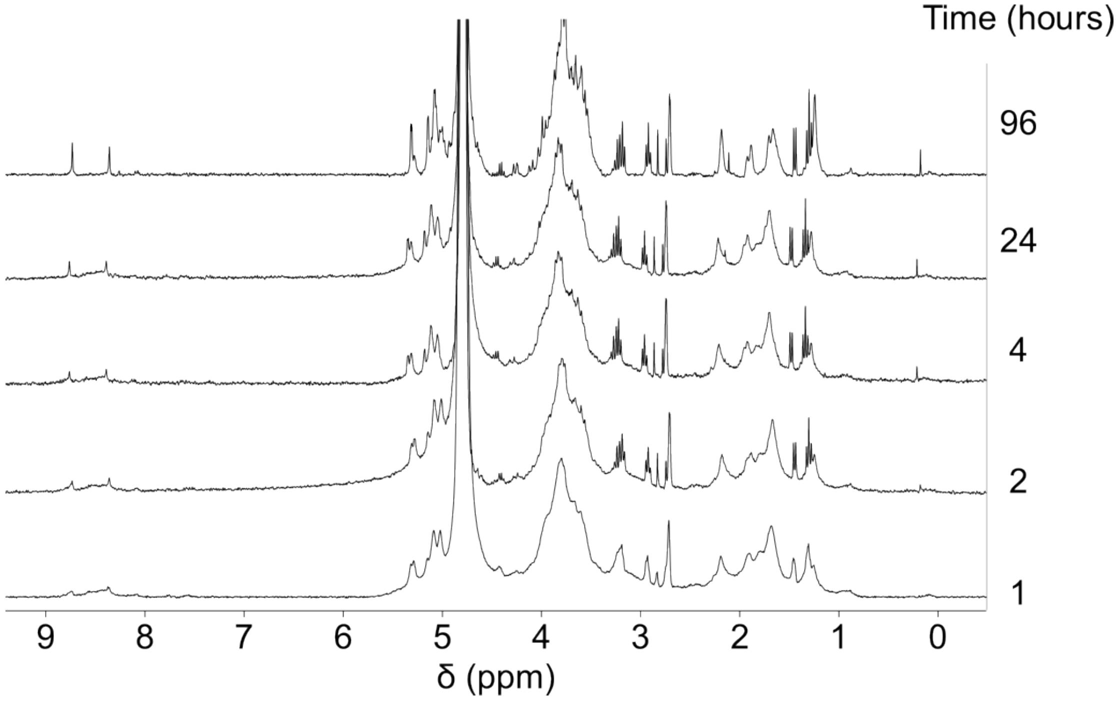

Figure S 4. Stack of ^{1}H-NMR spectra (D_2O, 300 MHz, 300 K) of compound **3-out•H⁺** at 3 mM in an acidic solution (pH = 2) heated at 323 K from 1 hour (bottom spectrum) to 96 hours (top spectrum) showing a progressive interconversion of **3-out•H⁺** to **3-in•H⁺**.

SI.5 Behavior of **3** in D_2O/DMSO-d_6 mixtures at pH < 2 and pH > 8

SI.5.1 **3-in**/**3-out** equilibration in 100% DMSO-d_6

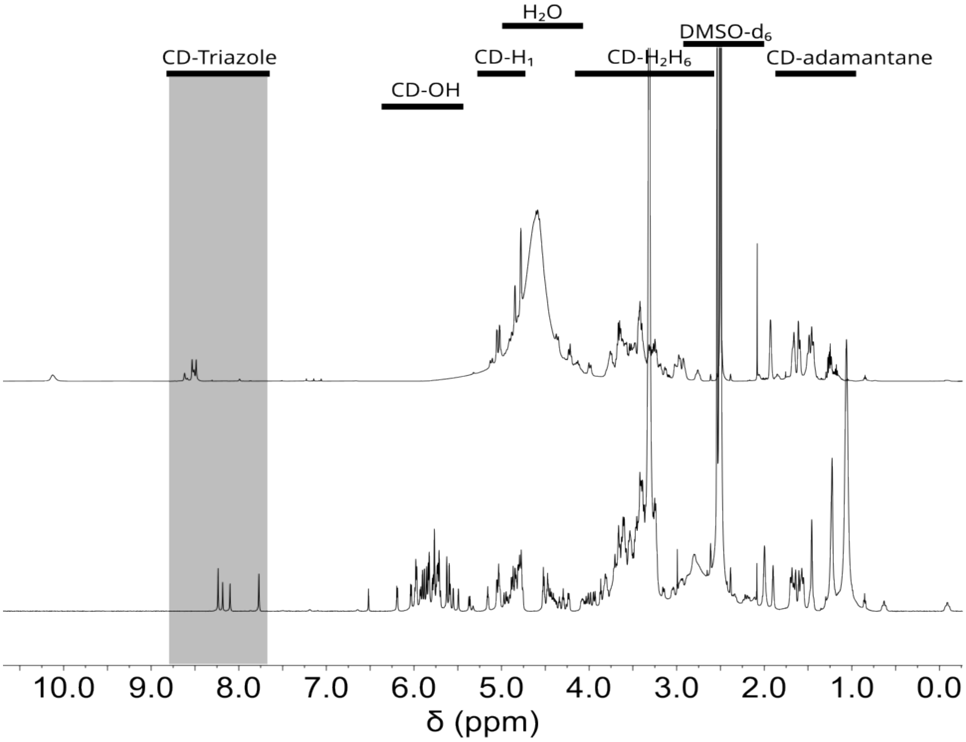

Figure S 5. ^{1}H-NMR (DMSO-d_6, 600 MHz, 300 K) spectra of compound **3** in an acidic solution (top spectrum, similar to pH < 2) affording a 7:93 ratio of **3-in•H+** / **3-out•H+** respectively, and a basic solution (bottom spectrum, similar to pH > 8) affording a 60:40 ratio of **3-in** / **3-out** respectively.

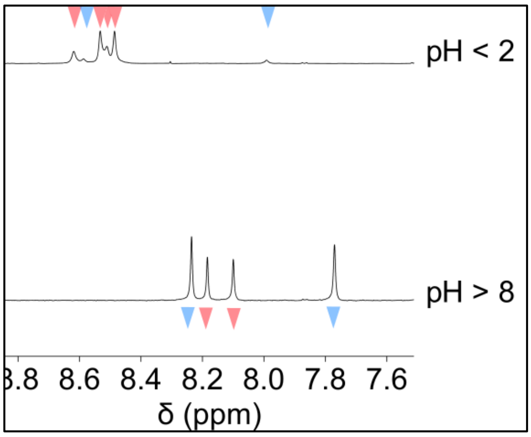

Figure S 6. Zoom of the triazole protons region showing the equilibrated species **3-out/3-out•H+** (red arrow) and species **3-in/3-in•H+** (blue arrow). In the case of the acidic conditions, **3-out•H+** is adopting two conformations in slow equilibrium on the NMR time scale (4 red triangles, see kinetic study SI.5.3.3.1).

SI.5.2 **3-in/3-out** equilibration in D_2O/DMSO-*d*$_6$ mixtures at pH > 8

SI.5.2.1 Sample preparation

3-in•H⁺ was dissolved in DMSO-*d*$_6$ (4.1 mM) with Et_3N (5.4 mM) and a second solution of **3-in•H⁺** was dissolved in D_2O (4.1 mM) and adjusted to pH > 8 with NaOD. Then the two solutions were mixed to get the desired ratio of solvents.

SI.5.2.2 ^{1}H NMR spectra of the equilibrated samples

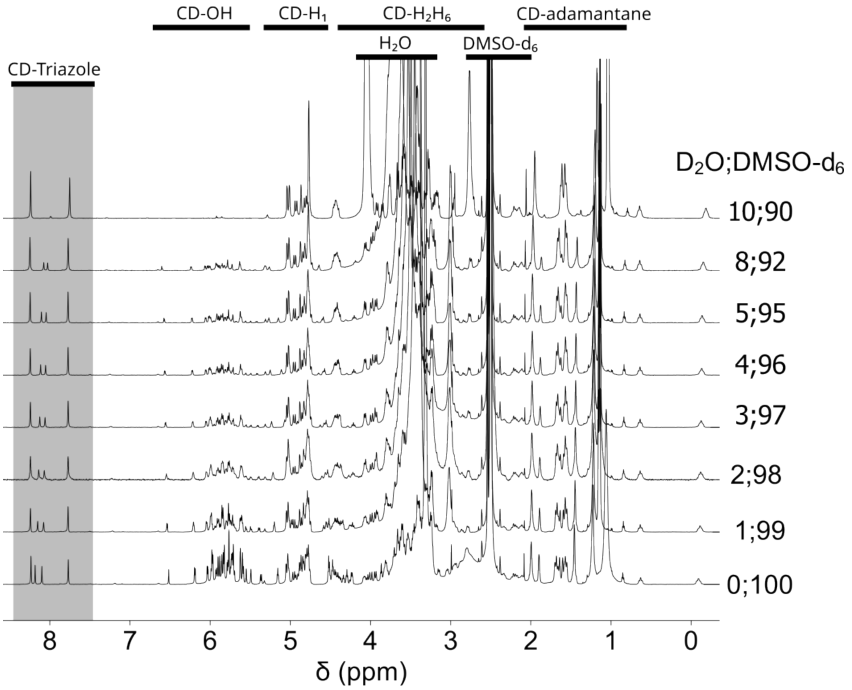

Figure S 7. ^{1}H-NMR (DMSO-*d*$_6$, 400 MHz, 300 K) stack of spectra of compound **3** in a basic solution (similar to pH > 8) at different ratio of a D_2O/DMSO-*d*$_6$ mixture ranging from D_2O:DMSO-*d*$_6$, 10:90 (top spectrum) to pure DMSO-*d*$_6$ (bottom spectrum).

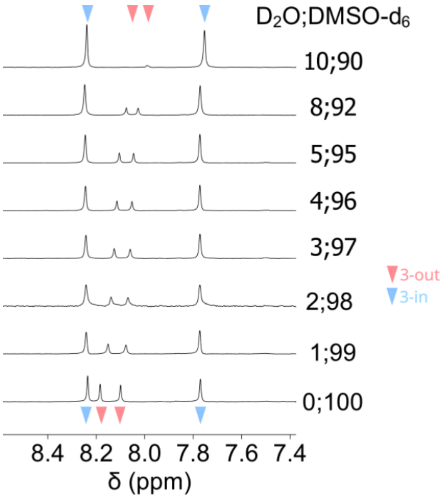

Figure S 8. Zoom of the triazole protons region showing the equilibrated species. Quasi total disappearance of **3-out** (red arrow) is observed at 10% D_2O in DMSO-*d*$_6$.

SI.5.2.3 Summary of **3-in**/**3-out** equilibration in D_2O/DMSO-d_6 mixtures

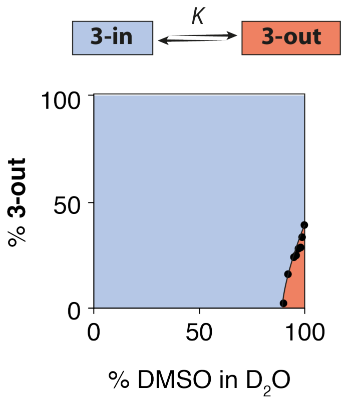

% DMSO in D_2O	K
100	0.64
99	0.49
98	0.40
97	0.39
96	0.33
95	0.31
92	0.19
90	0.02

Equilibration of **3-in** and **3-out** in pure DMSO-d_6 or aqueous DMSO-d_6 occurs rapidly (less than 2 min.)

SI.5.3 **3-in•H⁺/3-out•H⁺** equilibration in D_2O/DMSO-d_6 mixtures at pH < 2

SI.5.3.1 Sample preparation

3-in•H⁺ (acidified using either TFA-*d* or DCl) was dissolved in both DMSO-d_6 and D_2O (4.1 mM for both) and mixed to get the desired ratio of solvents. In acidic conditions, the **3-in•H⁺** and **3-out•H⁺** isomers equilibrate to reach a plateau at a rate dependent on the solvent ratio. Therefore, the evolution of each solvent ratio has been monitored as described below (SI.5.3.3).

SI.5.3.2 ^{1}H NMR spectra of the equilibrated samples

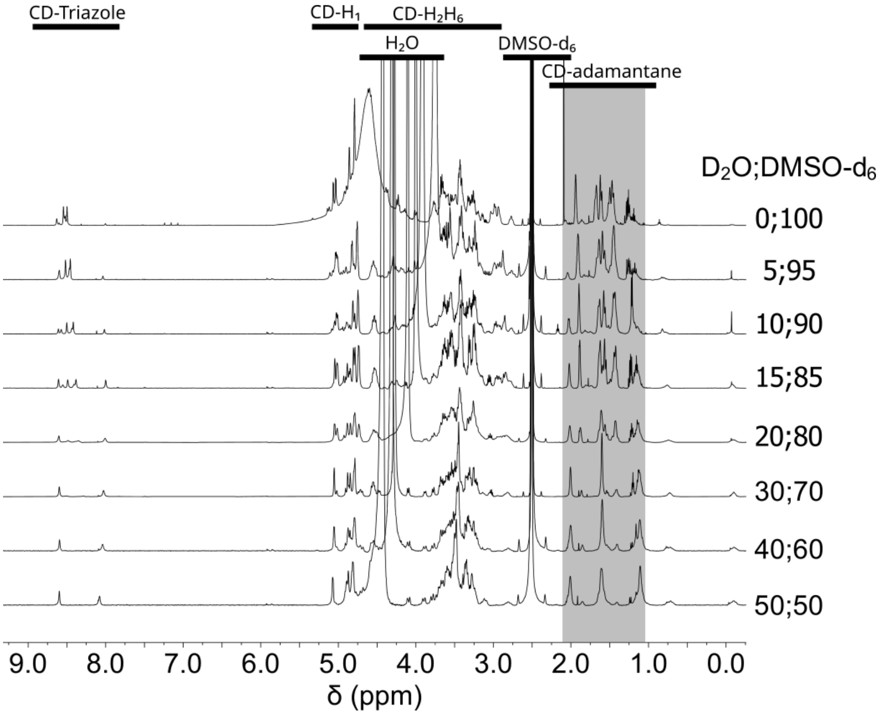

Figure S 9. ^{1}H-NMR (DMSO-d_6, 400 MHz, 300 K) stack of spectrum of compound **3** in an acidic solution (similar to pH < 2) at different ratio of a D_2O:DMSO-d_6 mixture ranging from 50:50 (bottom spectrum) to pure DMSO-d_6 (top spectrum).

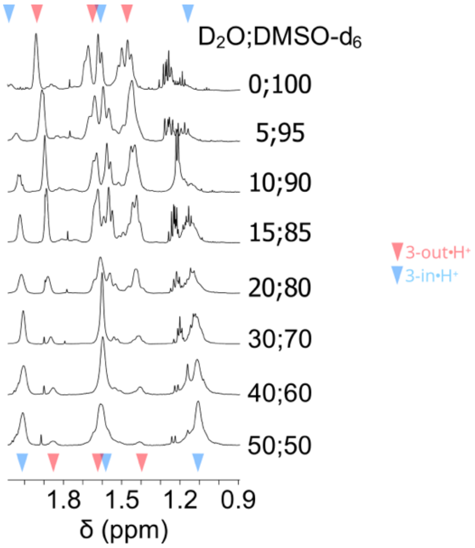

Figure S 10. Zoom of the adamantyl protons region showing the equilibrated species.

SI.5.3.3 ^{1}H NMR monitoring of the equilibration

SI.5.3.3.1 100 % DMSO-d_6

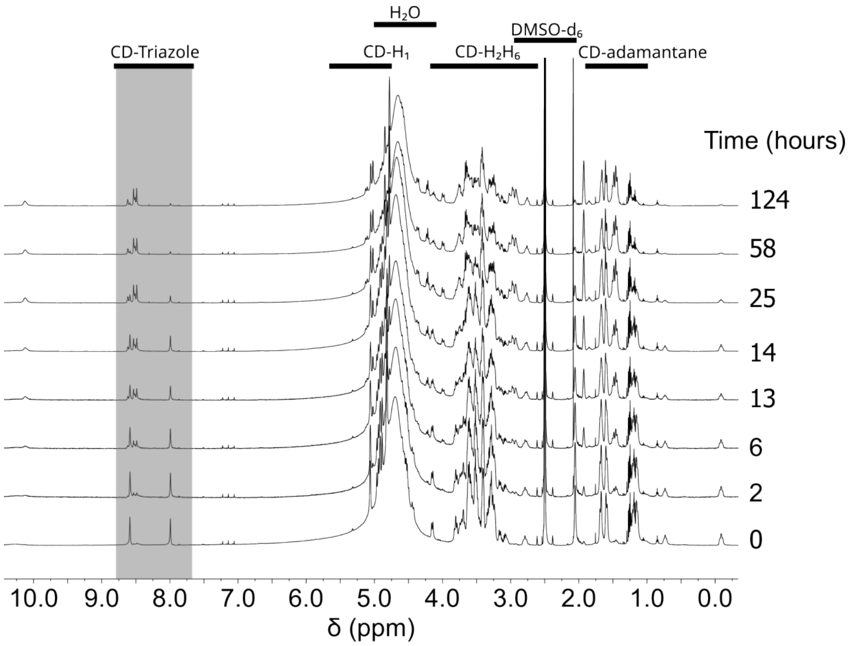

Figure S 11. ^{1}H-NMR (DMSO-d_6, 400 MHz, 300 K) stack of spectra of the evolution of **3-in•H⁺** in an acidic solution (similar to pH < 2) at different equilibrating times.

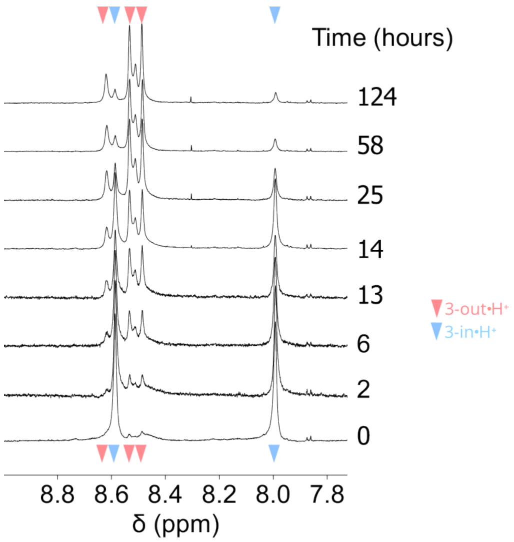

Figure S 12. Zoom of the triazole protons region. The equilibration of **3-in•H⁺** (blue arrow) : **3-out•H⁺** (red arrow) at 124 h leads to a ratio of 7:93.

SI.5.3.3.2 95% DMSO-d_6, 5% D_2O

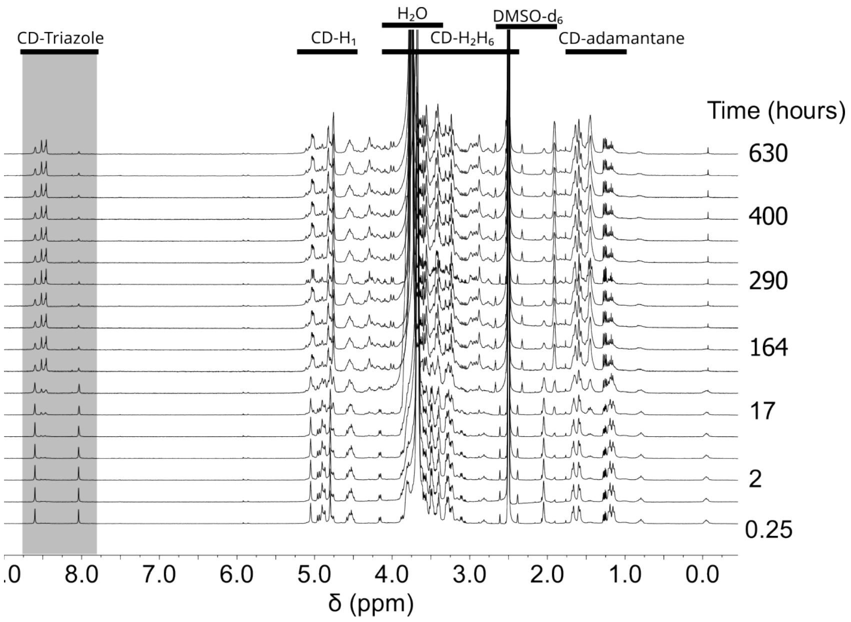

Figure S 13. ^{1}H-NMR (D_2O:DMSO-d_6 – 5:95, 400 MHz, 300 K) stack of spectra of the evolution of **3-in•H$^+$** in an acidic solution (similar to pH < 2) at different equilibrating times.

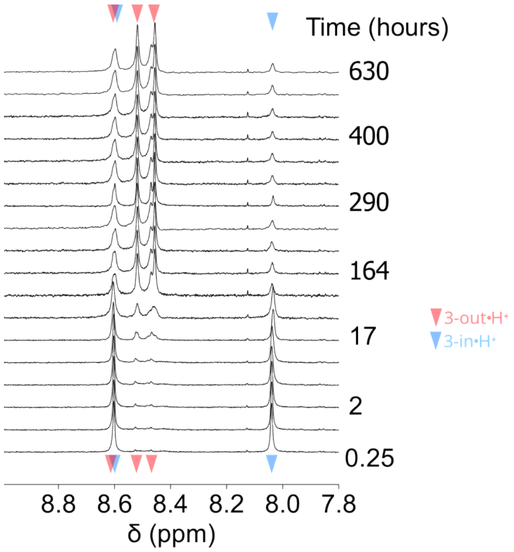

Figure S 14. Zoom of the triazole protons region. The equilibration of **3-in•H$^+$** (blue arrow) : **3-out•H$^+$** (red arrow) at 630 h leads to a ratio of 11:89.

SI.5.3.3.3 Equilibration at 90% DMSO-d$_6$, 10% D$_2$O

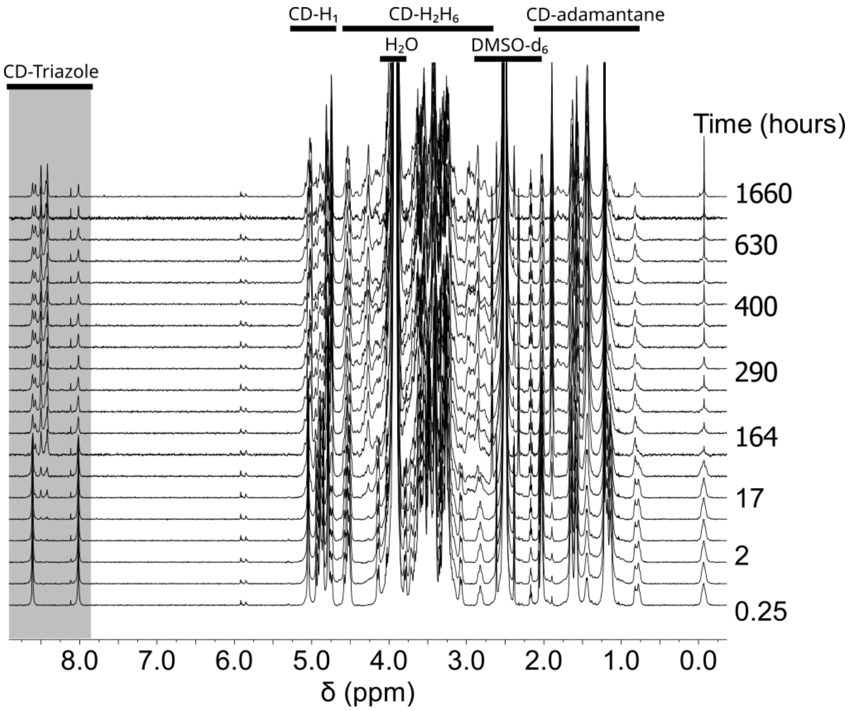

Figure S 15. ^{1}H-NMR (D$_2$O:DMSO-*d*$_6$ – 10:90, 400 MHz, 300 K) stack of spectra of the evolution of **3-in•H$^+$** in an acidic solution (similar to pH < 2) at different equilibrating times.

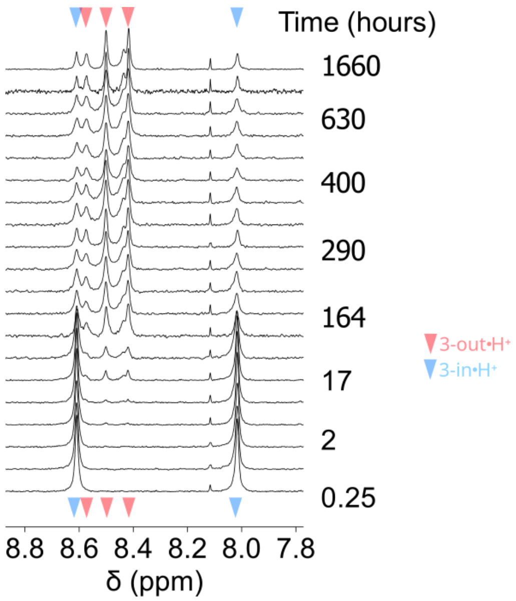

Figure S 16. Zoom of the triazole protons region. The equilibration of **3-in•H$^+$** (blue arrow) : **3-out•H$^+$** (red arrow) at 1660 h leads to a ratio of 21:79.

SI.5.3.3.4 Equilibration at 85% DMSO-d_6, 15% D_2O

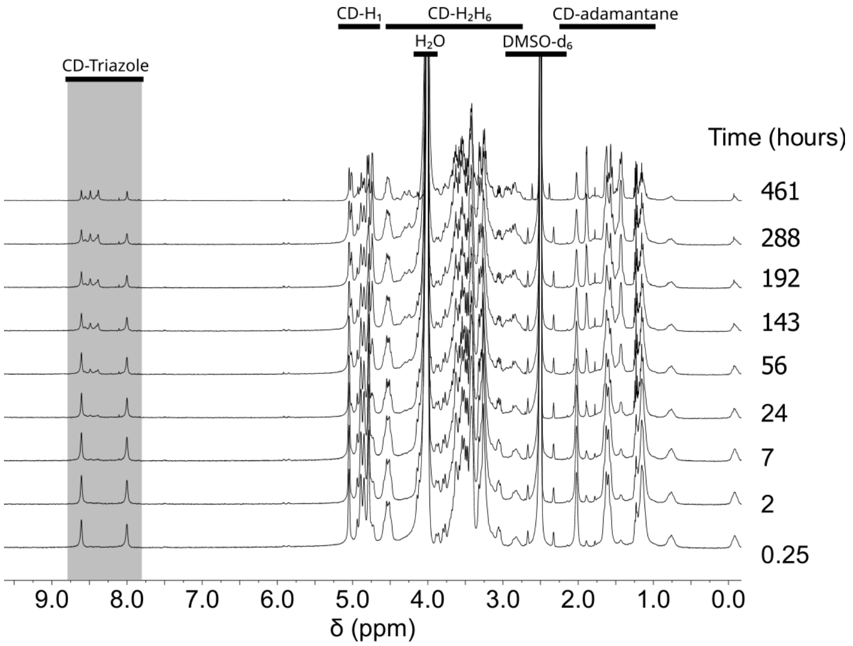

Figure S 17. ^1H-NMR (D_2O:DMSO-d_6 – 15:85, 400 MHz, 300 K) stack of spectra of the evolution of **3-in•H+** in an acidic solution (similar to pH < 2) at different equilibrating times.

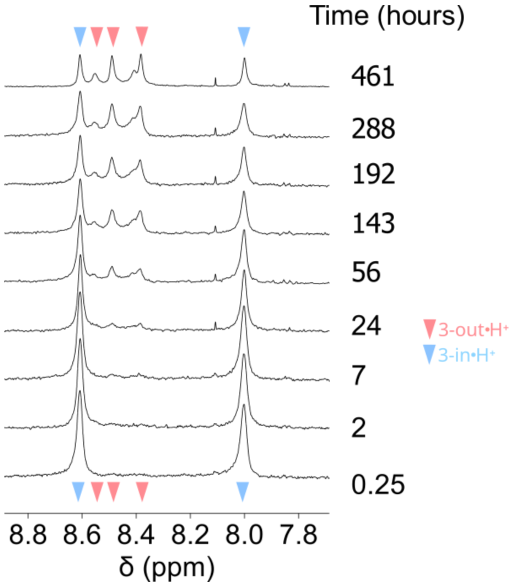

Figure S 18. Zoom of the triazole protons region. The equilibration of **3-in•H+** (blue arrow) : **3-out•H+** (red arrow) at 461 h leads to a ratio of 37:63.

SI.5.3.3.5 Equilibration at 80% DMSO-d_6, 20% D_2O

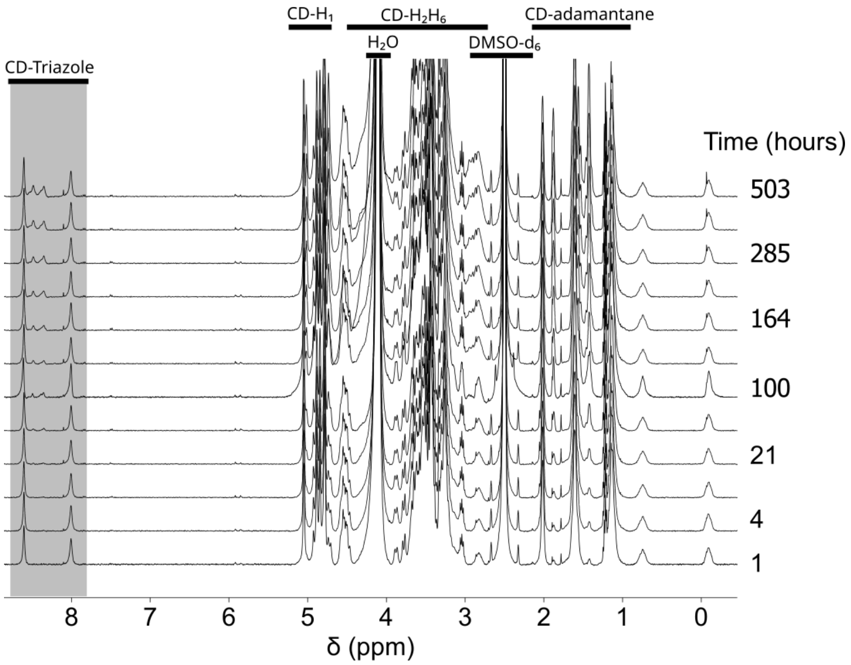

Figure S 19. ^{1}H-NMR (D_2O:DMSO-d_6 – 20:80, 400 MHz, 300 K) stack of spectra of the evolution of **3-in•H+** in an acidic solution (similar to pH < 2) at different equilibrating times.

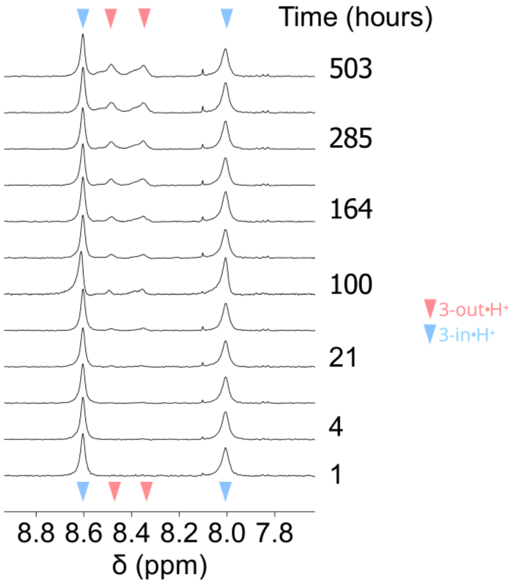

Figure S 20. Zoom of the triazole protons region. The equilibration of **3-in•H+** (blue arrow) : **3-out•H+** (red arrow) at 503 h leads to a ratio of 69:31.

SI.5.3.3.6 Equilibration at 70% DMSO-d6, 30% D2O

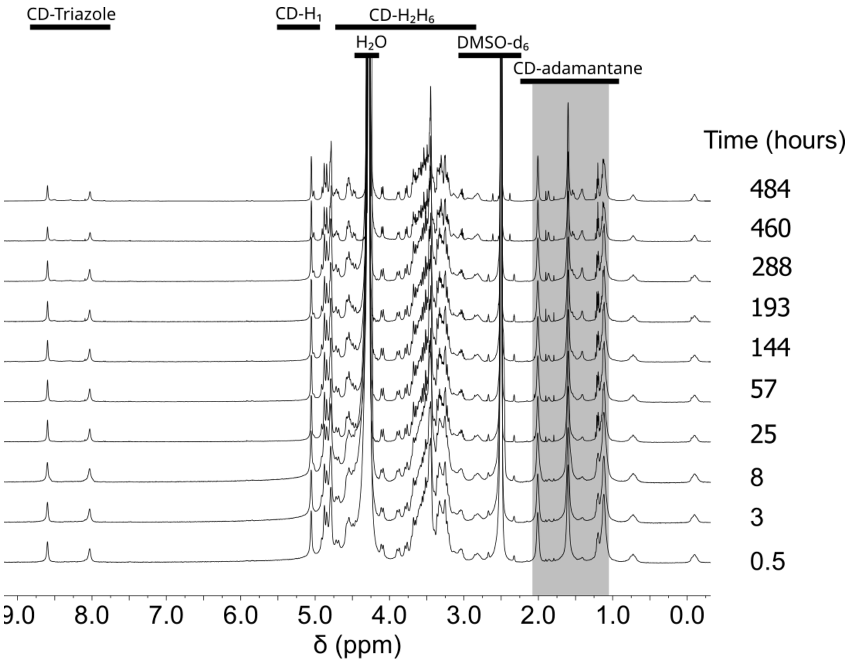

Figure S 21. ¹H-NMR (D_2O:DMSO-*d*6 – 30:70, 400 MHz, 300 K) stack of spectra of the evolution of **3-in•H+** in an acidic solution (similar to pH < 2) at different equilibrating times.

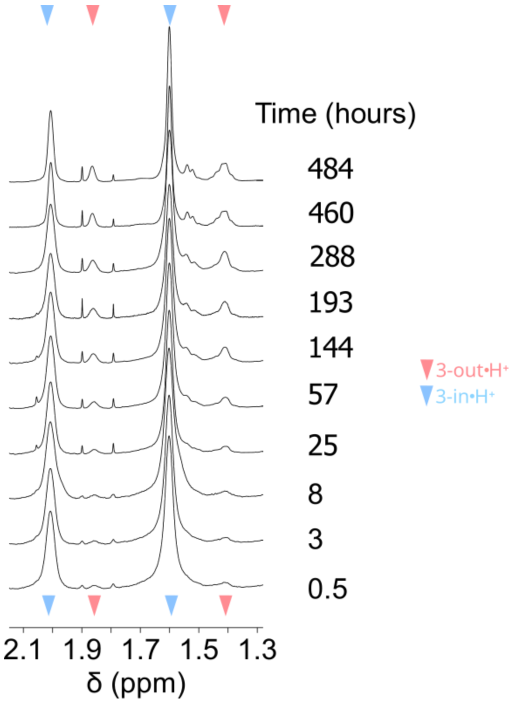

Figure S 22. Zoom of the adamantyl Hb and Hc+Hc' protons region. The equilibration of **3-in•H+** (blue arrow) : **3-out•H+** (red arrow) at 484 h leads to a ratio of 85:15. Triazole protons of *in* and *out* isomers broaden and overlap during the monitoring.

SI.5.3.3.7 Equilibration at 60% DMSO-d$_6$, 40% D_2O

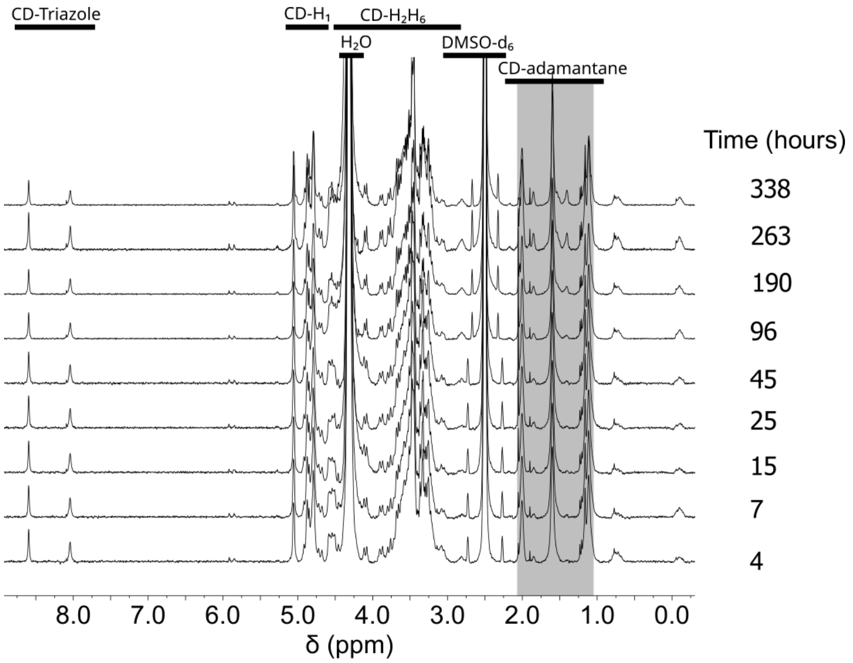

Figure S 23. ^{1}H-NMR (D_2O:DMSO-*d*$_6$ – 40:60, 400 MHz, 300 K) stack of spectra of the evolution of **3-in•H$^+$** in an acidic solution (similar to pH < 2) at different equilibrating times.

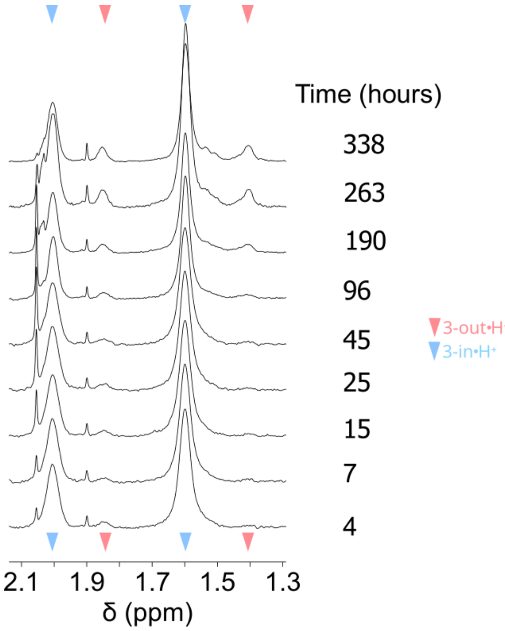

Figure S 24. Zoom of the adamantyl Hb and Hc+Hc' protons region. The equilibration of **3-in•H$^+$** (blue arrow) : **3-out•H$^+$** (red arrow) at 338 h leads to a ratio of 89:11. Triazole protons of *in* and *out* isomers broaden and overlap during the monitoring.

SI.5.3.3.8 Equilibration at 50% DMSO-d_6, 50% D_2O

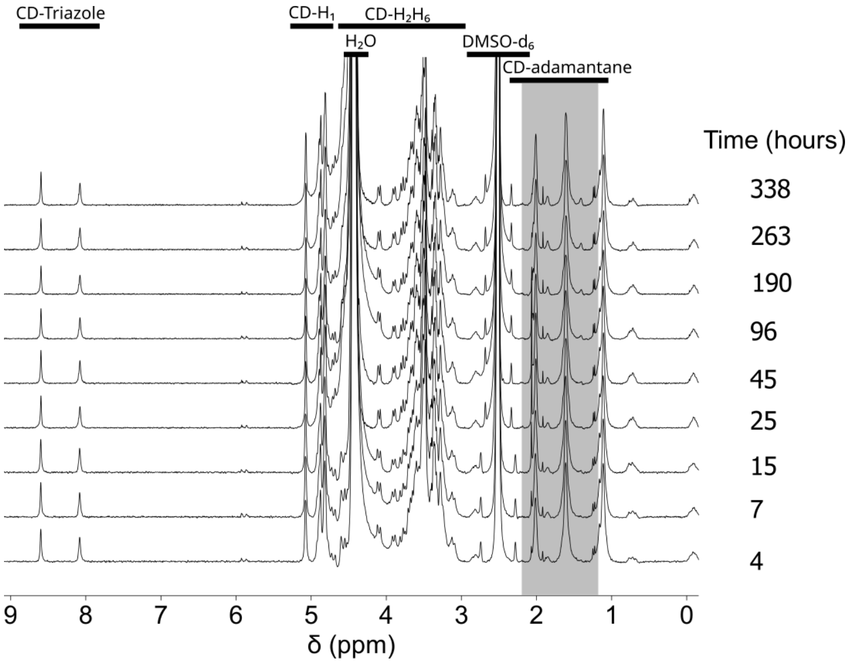

Figure S 25. ^{1}H-NMR (D_2O:DMSO-d_6 – 50:50, 400 MHz, 300 K) stack of spectra of the evolution of **3-in•H+** in an acidic solution (similar to pH < 2) at different equilibrating times.

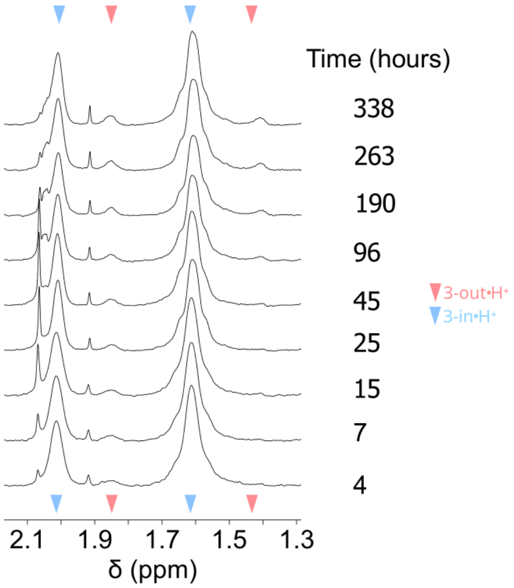

Figure S 26. Zoom of the adamantyl Hb and Hc+Hc' protons region. The equilibration of **3-in•H+** (blue arrow) : **3-out•H+** (red arrow) at 338 h leads to a ratio of 93:7. Triazole protons of *in* and *out* isomers broaden and overlap during the monitoring.

SI.5.3.4 Summary of the results

The slow equilibration of **3-in** and **3-out** in 50% to 100% DMSO in D_2O was monitored up to 500 h (see graphs below). The interconversion of **3-in** and **3-out** follows a first order kinetic rate according the following equation (1) :

$$\frac{d[\mathbf{3\text{-}out{\bullet}H^+}]}{dt} = k_1 \times [\mathbf{3\text{-}in{\bullet}H^+}] - k_{-1} \times [\mathbf{3\text{-}out{\bullet}H^+}] \qquad (1)$$

with $$C_{tot} = [\mathbf{3\text{-}in{\bullet}H^+}] + [\mathbf{3\text{-}out{\bullet}H^+}] \qquad (2)$$

thus $$[\mathbf{3\text{-}in{\bullet}H^+}] = C_{tot} - [\mathbf{3\text{-}out{\bullet}H^+}]$$

hence $$\frac{d[\mathbf{3\text{-}out{\bullet}H^+}]}{dt} = -(k_1 + k_{-1}) \times [\mathbf{3\text{-}out{\bullet}H^+}] + k_1 \times C_{tot} \qquad (3)$$

affording the following solution :

$$[\mathbf{3\text{-}out{\bullet}H^+}] = C_{tot} \times \frac{k_1}{k_1 + k_{-1}} \times \left[1 - e^{-(k_1 + k_{-1})t}\right] \qquad (4)$$

The collected kinetic data were fitted to equation 4 using LabPlot, yielding the following kinetic constants (see table). The corresponding equilibrium constants ($K_{in/out}$) are measured with the ^{1}H NMR ratio of the equilibrated species.

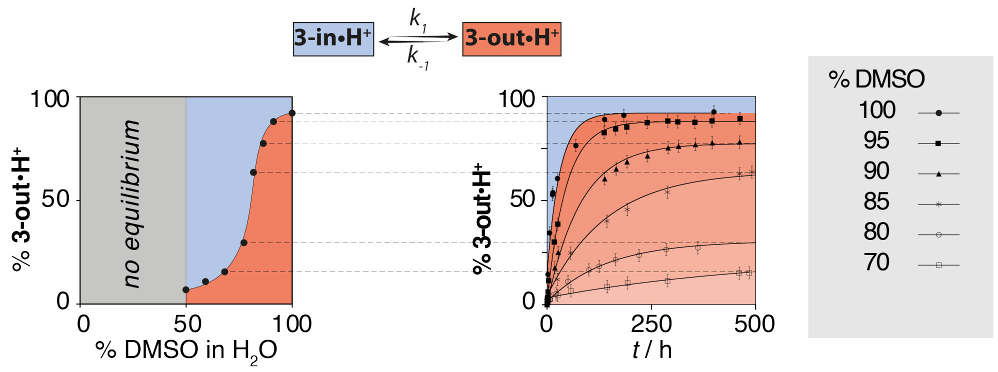

% DMSO in water	$K_{out/in}$	k_1 (s^{-1})	k_{-1} (s^{-1})
100	15.7	8.3 (±0.3) x 10^{-6}	0.5 (±0.5) x 10^{-6}
95	8.1	5.0 (±0.3) x 10^{-6}	0.6 (±0.6) x 10^{-6}
90	3.7	2.7 (±0.5) x 10^{-6}	0.7 (±0.5) x 10^{-6}
85	1.7	1.3 (±0.3) x 10^{-6}	0.6 (±0.4) x 10^{-6}
80	0.4	0.7 (±0.6) x 10^{-6}	1.7 (±0.6) x 10^{-6}
70	0.2	*nd*	*nd*
60	0.1	*nd*	*nd*
50	0.06	*nd*	*nd*

nd: not determined

SI.6 Theoretical study

SI.6.1 Procedure

The calculations were performed with the xTB software in its version 6.1,[3] employing the GFN2-xTB parametrization.[4] Practically, we started by simulating the non-protonated CD **3-in** conformation, and the conformational space was explored using the CREST software[5] in order to localize lower conformers. The lowest energy conformer (44% weight at 298 K) was used as a starting point for understanding the extrusion of the adamantyl moiety from the CD **3-in** cavity. We placed a methane at 30 Å distant from the adamantyl attachment point to serve as an anchor for displacing the adamantyl outside of the cavity and performed the calculation in implicit water. We then studied the protonated form by introducing the proton on various nitrogens using the same protocol as the one previously described.

For each set of conformations for each protonation state (adamantyl inside and outside the cavity and transition state), a DFT single point calculation was then performed. Following previous studies done in our group, we chose to use the B3LYP functional complemented by the D3 dispersion scheme and the basis set used was def2-SV(P). Implicit solvation was incorporated through the COSMO implementation available in Turbomole V6.4.

SI.6.2 Energies

DFTB

**3-in**[a]	**3-TS**[a]	**3-out**[a]	Barrier for extrusion[b]	Enthalpy[b]	Barrier for insertion[b]
-335.057178	-335.0297787	-335.045674	17.19	7.22	**9.97**

**3-in•H**$^{+a}$	**3-TS•H**$^{+a}$	**3-out•H**$^{+a}$	Barrier for extrusion[b]	Enthalpy[b]	Barrier for insertion[b]
-335.243054	-335.201041	-335.224744	26.36	11.49	**14.87**

[a] Energies are in Hartree units; [b] Energies are in kcal.mol^{-1}

DFT

**3-in**[a]	**3-TS**[a]	**3-out**[a]	Barrier for extrusion[b]	Enthalpy[b]	Barrier for insertion[b]
-5241.729325	-5241,6904	-5241.713635	24.43	9.85	**14.58**

**3-in•H**$^{+a}$	**3-TS•H**$^{+a}$	**3-out•H**$^{+a}$	Barrier for extrusion[b]	Enthalpy[b]	Barrier for insertion[b]
-5242.191115	-5242.136407	-5242.184819	34.33	3.95	**30.38**

[a] Energies are in Hartree units; [b] Energies are in kcal.mol^{-1}

---

[3] C. Bannwarth, E. Caldeweyher, S. Ehlert, A. Hansen, P. Pracht, J. Seibert, S. Spicher, S. Grimme, *WIREs Comput. Mol. Sci.* **2021**, *11*, e1493.
[4] C. Bannwarth, S. Ehlert, S. Grimme, *J. Chem. Theory Comput.* **2019**, *15*, 1652–1671.
[5] P. Pracht, F. Bohle, S. Grimme, *Phys. Chem. Chem. Phys.* **2020**, *22*, 7169–7192.

SI.6.3 DFT calculated conformations of the self-inclusion mechanism

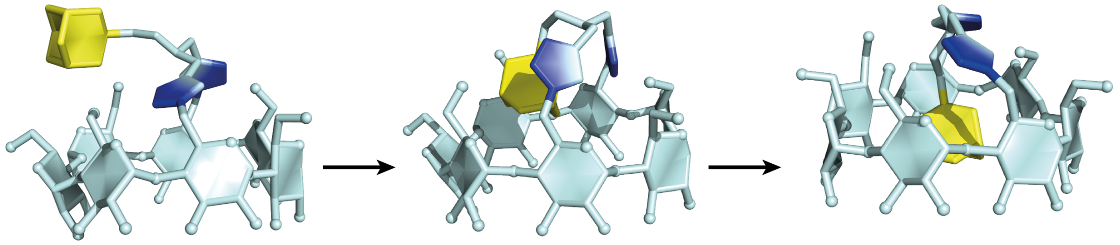

3-out **3-TS** **3-in**

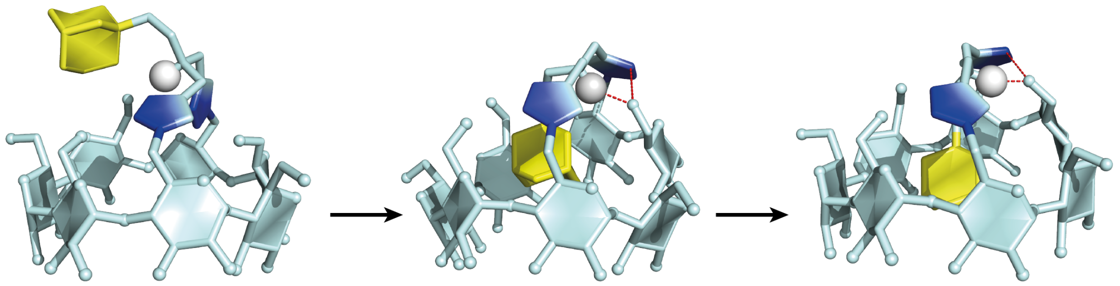

3-out·H⁺ **3-TS·H⁺** **3-in·H⁺**

Scheme S 1. Calculated 3D models of CDs **3-out**, **3-in** and **3-TS** (transition-state of the self-inclusion reaction) in both protonated and unprotonated forms.

SI.7 NMR DOSY - degree of polymerization (DP)

The diffusion coefficient provides information about the size of the molecule and thus the degree of polymerization. It is assumed that the formed supramolecular polymer is linear and rigid, therefore a cylinder-like structure is used as model. The dimensions of this structure can be estimated by the Tirado-Garcia de la Torre relationship:[6]

$$D = \frac{k_B T}{3\pi\eta L}\left[ln\left(\frac{L}{d}\right) + \nu\right]$$

with:

$$\nu = 0.312 + 0.565\frac{d}{L} - 0.100\left(\frac{d}{L}\right)^2$$

where:

- D Translational diffusion coefficient ($m^2.s^{-1}$)
- k_B Boltzmann constant ($J.K^{-1}$)
- T Temperature (K)
- η Viscosity of the liquid (Pa.s)
- L Length of cylinder (m)
- d Diameter of cylinder (m)

According to Garcia de la Torre *et al.*, introducing the terms and a finite aspect ratio (*p*), the translational diffusion coefficient D can be expressed as:

$$\frac{3D\pi\eta d}{k_B T} = \frac{1}{p}\left[\ln(p) + 0.312 + \frac{0.565}{p} - \frac{0.100}{p^2}\right] \qquad \text{with} \qquad p = \frac{L}{d}$$

The left-hand side of the equation can be evaluated from the measured diffusion coefficient and assuming that the cylinder diameter of CD **3-out•H⁺** is the same as for β-cyclodextrin (diameter of the β-CD d = 1.54 nm).[7] This equation has no analytical solution, but the value of p can be determined graphically by plotting the curve y = f(p). Finally, the degree of polymerization (DP) at a given concentration was obtained from p by considering that the dimension of the repeat unit in the self-assembled polymer **3-out•H⁺** is 0.9 nm. Figure S2 shows an example of calculation of the degree of polymerization for compound **3-out•H⁺** at 21 mM (Figure S2). The formed polymer is composed of 22 monomer units.

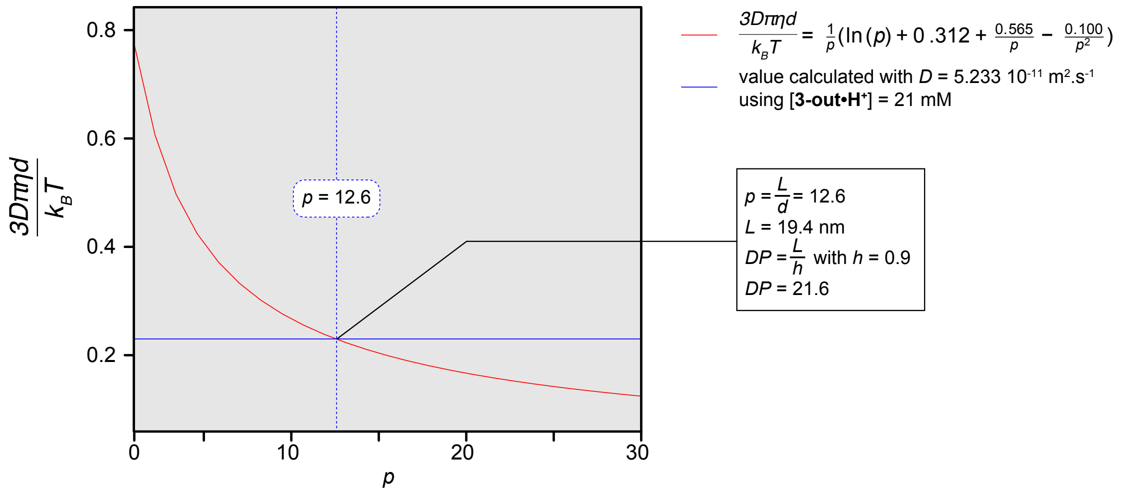

Figure S 27. Example of the calculation of p for a solution of **CD 3-out·H⁺** at 21 mM.

---

[6] J.G. De La Torre, M.C.L. Martinez, M.M. Tirado, *Biopolymers*, **1984**, *23*, 611-615.
[7] G.M. Pavlov, E.V. Korneeva, N.A. Smolina, U.S. Schubert, *Eur. Biophys. J.* **2010**, *39*, 371-379.

SI.8 ITC experiments – host/guest association

ITC experiments were performed at 20°C with the MicroCal iTC200 microcalorimeter from Malvern Panalytical. Injections of 2µL of a concentrated CD solution were realized stepwise by a 40µL microsyringe at an interval of 180s into a sample cell of 202.7µL filled with distilled water while stirring at 750rpm. The amount of heat produced per injection was calculated by integration of the area under each peak by the instrument software "Origin for ITC". The data obtained were fitted according to reference.[8] The dilution of **3-out•H⁺** produces a strongly concentration dependent endothermic signal caused by the dissociation of inclusion complexes of the supramolecular polymer. The calculated apparent association constant of monomer **3-out•H⁺** was approximately K_a= 9 x 10^3 M^{-1}. This value implies a DP_n = 14 at 21 mM,[8] in good agreement with the DOSY data.

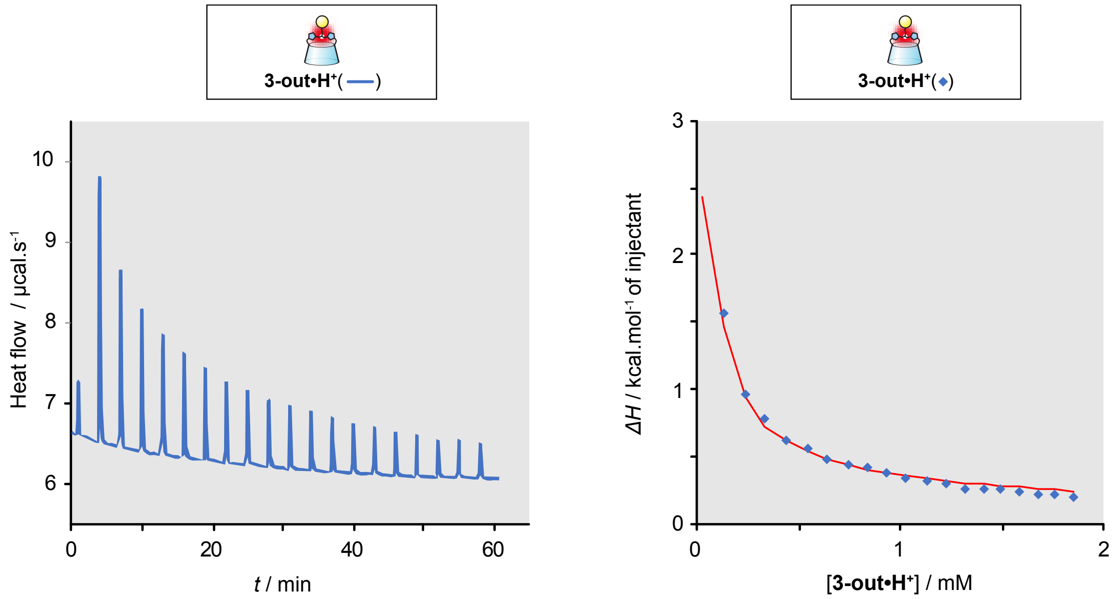

Figure S 28. a) Heat flow curves obtained by dilution of a 11 mM solution of **3-out•H⁺**. b) Corresponding ITC enthalpogram; the continuous curve is a fit according to an isodesmic model.[8]

Table S 1.Thermodynamic parameters of inclusion complexes formationfor CD **1-in** and **3-out•H⁺** derived from ITC dilution experiments.

	ΔG / Kcal.mol⁻¹	K_a / L.mol⁻¹	ΔH / Kcal.mol⁻¹	-TΔS / Kcal.mol⁻¹
**1-in** [(a),9]	-7.8	11 x 10^3	-2.9	-4.9
**3out•H⁺** [(b)]	-7.6	9.0 x 10^3	-3.1	-4.5

[(a)]global isodesmic fit for 7 solutions of concentrations ranging from 0.5 to 13mM

[(b)]global isodesmic fit for 2 solutions of concentrations of 5 and 10.7mM

[8] Considering $Dp = \sqrt{KC}$ according to A. Arnaud, L. Bouteiller, *Langmuir* **2004**, *20*, 6858–6863.

[9] P. Evenou, J. Rossignol, G. Pembouong, A. Gothland, D. Colesnic, R. Barbeyron, S. Rudiuk, A.-G. Marcelin, M. Ménand, D. Baigl, V. Calvez, L. Bouteiller, M. Sollogoub, *Angew. Chem., Int. Ed.* **2018**, *57*, 7753–7758.

SI.9 Viscometry study of the assembly

Viscometry measurements were performed at 25°C on an automatic Anton-Paar AMVn viscometer using a capillary with an internal diameter of 1.6 mm and a steel ball diameter of 1.5 mm. For each sample, 6 flow time measurements were realized at a tilt angle of 20° and the average value was then normalized by the corresponding flow time of water to obtain the relative viscosity. Measurements were performed on solutions of CD **3-out•H$^+$** and compared to **1-in**, **2-out** and blank solutions of **β-CD**.

The evolution of relative viscosity with concentration of compound **1-in** follows the same trend as the one of **β-CD**. This confirms the non-associative behavior of compound **1-in**.

On the contrary, compounds **2-out** and **3-out•H$^+$** show a non-linear increase of relative viscosity with concentration. These results indicate the presence of anisotropic structures in these samples. Indeed, the transition from a monomeric species equivalent to a spherical particle, to a supramolecular polymer, equivalent to a cylindrical particle, induces a non-linear increase of the relative viscosity.[10]

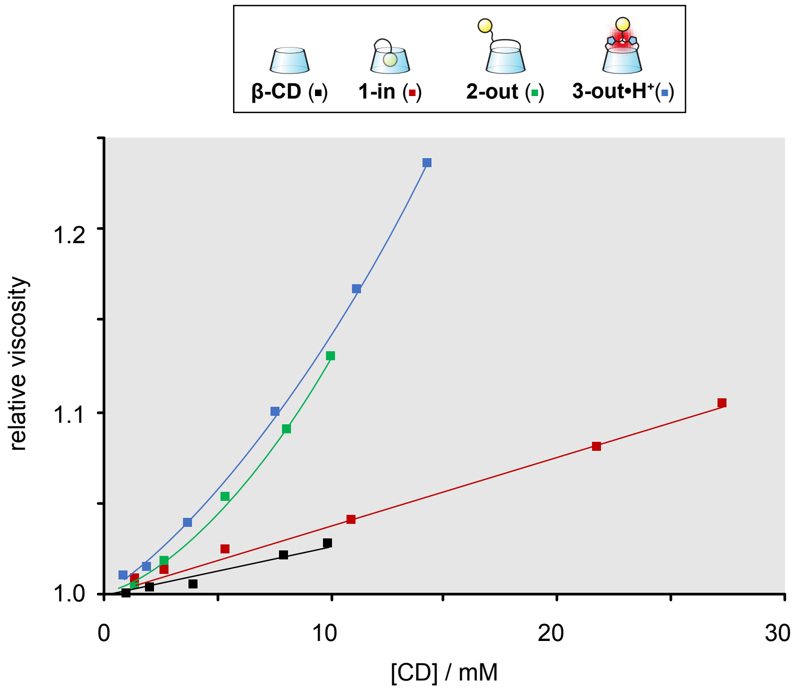

Figure S 29. Relative viscosity of native β-CD, CD **1-in**, **2-out**, and **3-out·H+** in aqueous solutions versus concentration. The lines are guides for the eyes.

[10] E. Obert, M. Bellot, L. Bouteiller, F. Andrioletti, C. Lehen-Ferrenbach, F. Boue *J. Am. Chem. Soc.* **2007**, *129*, 15601-15605

SI.10 SANS study of the assembly

Small-angle neutron scattering (SANS) measurements were made at the LLB (Saclay, France) on the PACE and PA20 instruments and at the ILL (Grenoble, France) on the D11 instrument. Two distance-wavelength combinations were used to cover the 6.9 × 10^{-3} to 0.37 $Å^{-1}$ q-range, where the wave vector q is defined as usual, assuming elastic scattering $q=(4\pi/\lambda)\sin(\theta/2)$), where θ is the angle between incident and scattered beam). Measurements were realized in D_2O at 25°C. Data were corrected for the empty cell signal and the solute and solvent incoherent background. A light water standard was used to normalize the scattered intensities to cm^{-1} units and the scattered intensities of each solution were normalized by concentration.

Results show that the intensity scattered by the bridged compounds, *i.e*. **2-out** and **3-out•H⁺**, as a function of wave vector (q) is higher than those of **1-in**, indicating the formation of larger objects. The intensity scattered by compounds **2-out** and **3-out•H⁺** in the intermediate wave vector region has a q^{-1} slope suggesting a rod shape for these objects.

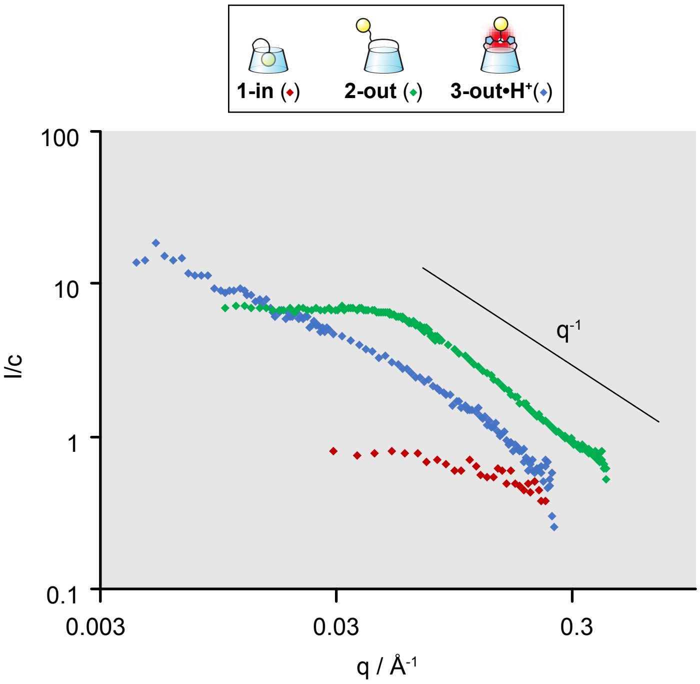

Figure S 30. SANS intensity normalized by concentration (I/c) versus wave vector (q) for solutions of CD **1-in**, **2-out** and **3-out•H⁺** in D_2O at 25°C.

SI.11 pKa determination by ^{1}H NMR

SI.11.1 pKa of **3-in** in D_2O

A sample of **3-in** (3.61 mg, 2.5 µmol) being fully self-included was dissolved in D_2O (500 µL, 5 mM), then the pH of the solution was adjusted to 10.4 using NaOD (2 M in D_2O). Then, pH titration of the solution was performed using DCl (37% w/w in D_2O, 0.5 to 2 µL, *ca.* 0.2 pH units) and monitored by ^{1}H NMR (D_2O, 400 MHz, 300 K, Figure S 31-a).

The signals of the protons of both triazole units (Figure S 31-a, red and black spots) were used to follow the protonation state and draw the corresponding titration curves δ_{Triaz} = *f*(pH) (Figure S 31-b).

a)

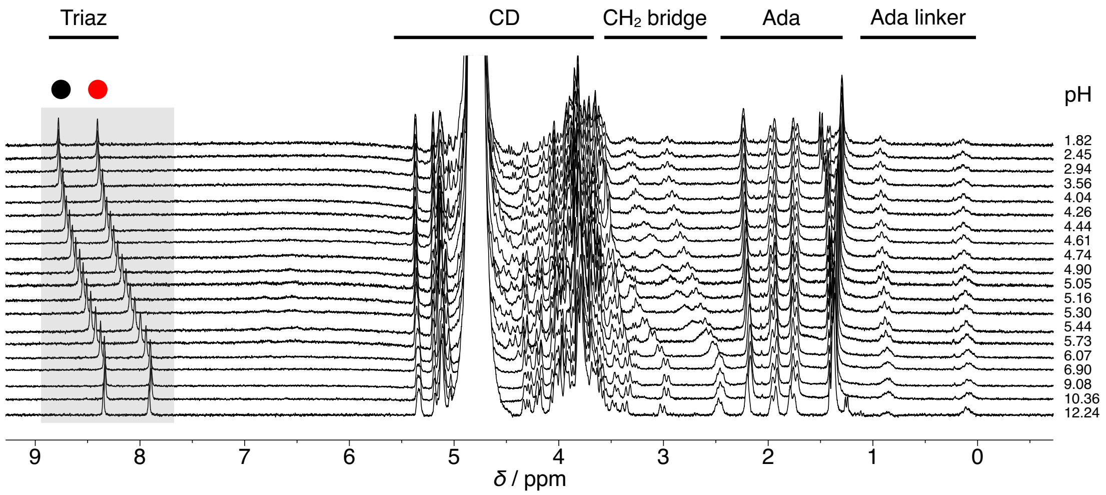

b)

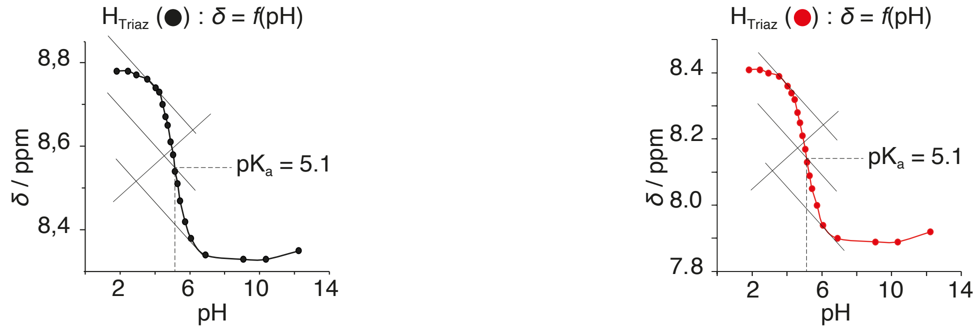

Figure S 31. (a) ^{1}H NMR spectra (D_2O, 400 MHz, 300 K) of the pH titration of **3-in**; (b) Titration curves obtained by monitoring the chemical shifts of the two triazole protons.

SI.11.2 pKa of **3-out** in DMSO-d_6

A sample of **3-out•H⁺** (3.59 mg, 2.5 µmol) being fully protonated was dissolved in DMSO-d_6 (500 µL, 5 mM); then a NOESY spectrum was recorded to assess the outer location of the adamantane unit. We then used a modified protocol described by Iggo *et al.* to determine the pK_a of **3-out** in DMSO-d_6.[11]

Internal references were added to the starting solution to extrapolate the pH values over a wide range (3 to 10 pH units) using 2,6-lutidine (0.29 µL, 2.5 µmol, 5 mM) and triethylamine (0.35 µL, 2.5 µmol, 5 mM) and the pH values were determined using the modified Henderson-Hasselbalch equation :

$$pH = pK_a + log_{10}\left[\frac{\delta_{obs} - \delta_H}{\delta_L - \delta_{obs}}\right]$$

^{1}H NMR (DMSO-d_6, 400 MHz, 300 K) titration of the solution was then performed using DCl (37% w/w in D_2O, 1 µL) affording the corresponding stack of spectra (Figure S 32-a).

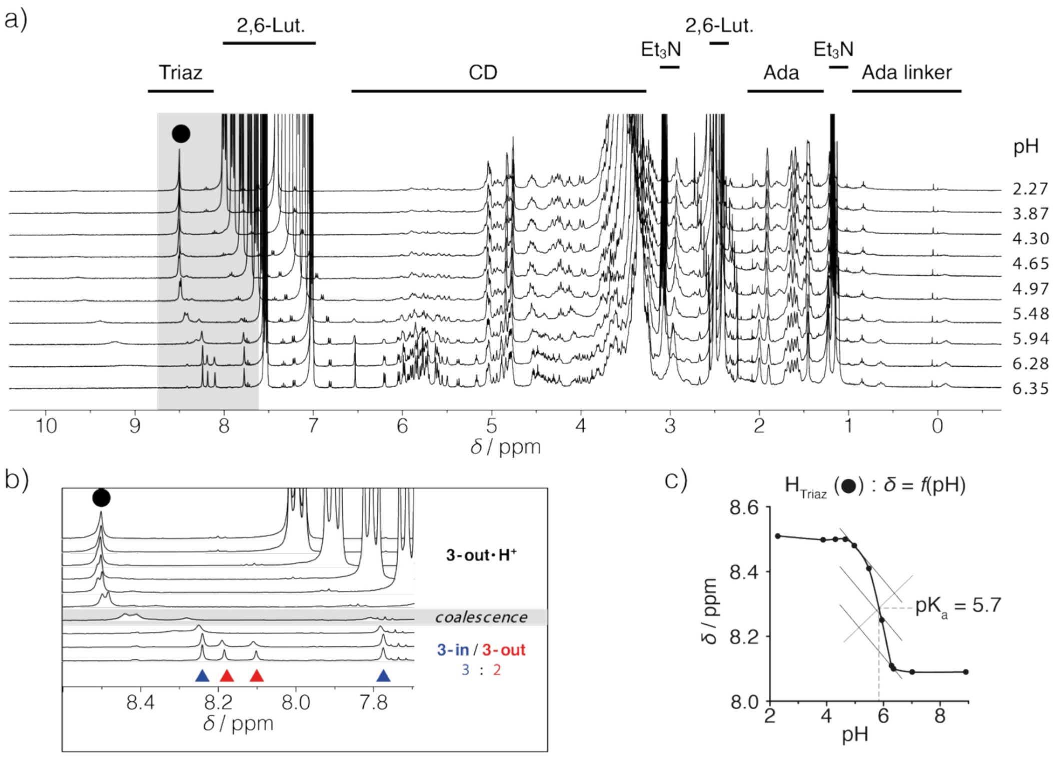

Figure S 32. (a) ^{1}H NMR spectra (DMSO-d_6, 400 MHz, 300 K) of the pH titration of **3-out**; (b) inset : zoom-in of the triazole protons region showing the modification in exchange regime; (c) Titration curve obtained by monitoring the chemical shifts of the triazole proton of the **3-out/3-out•H⁺** couple.

The chemical shift of one of the protons of the triazole units was used to follow the protonation state and draw the corresponding titration curve $\delta_{Triaz} = f(pH)$ (Figure S 32-c).
It has to be noted that during the course of the titration, a second regime of exchange appeared at the inflexion point. In this region, the followed signal undergoes a coalescence and splits into two different sets of signals (Figure S 32-b) attributed to the competitive self-inclusion of the adamantane (see SI.5.1).

[11] G. Schenck, K. Baj, J. A. Iggo, M. Wallace, *Anal. Chem.* **2022**, *94*, 8115–8119.

SI.12 pH- and time-dependent of **3-out•H⁺** into self-included monomer **3-in•H⁺**

SI.12.1 Preparation of the samples

3-in (3.3 mg, 2.6 µmol) was dissolved in DMSO-d_6 (500 µL), the solution was acidified with TFA (1 µL, 13 µmol, 5 equiv.) and maintained at room temperature for one week (see equilibration study in 100% DMSO-d_6 SI.5.3.3.1). The equilibrated solution was evaporated under reduced pressure and the resulting white solid was dissolved in pre-acidified D_2O (using DCl 37%, pH = 2) to obtain the **3-out•H$^+$** D_2O solution (**3-out•H$^+$**:**3-in•H$^+$**, 93:7 mixture, 4.8 mM, pH 1.56).

The **3-out•H$^+$** acidic solution was adjusted with NaOD to the desired pH. Then the evolution of the **3-in(•H$^+$)** and **3-out(•H$^+$)** isomers was monitored by ^{1}H NMR during 190 h.

*Remark : The protonation equilibria of both **3-in/3-in•H$^+$** and **3-out/3-out•H$^+$** are rapid on the NMR timescale. As a result, the species observed during kinetic monitoring are mixtures of these protonation states. For simplicity, these mixtures are referred to as **3-in** and **3-out**, respectively throughout the following kinetic experiment descriptions.*

SI.12.2 Quantification method

The proportions of **3-in** and **3-out** were measured by integration of the adamantyl signals (Ha, Hc and Hc'). The intensity of the isolated Ha signal from **3-in** ($i^{Ha}_{\textbf{3-in}}$, 6H, δ = 1.28 ppm) was compared with the intensity of the Ha signal from **3-out** ($i^{Ha}_{\textbf{3-out}}$, 6H, δ = 1.61-1.76 ppm). See Figure S 35.

However, the latter is overlapped with the Hc' signals of both **3-in** and **3-out** ($i^{Hc'}_{\textbf{3-in}}$, 3H, and $i^{Hc'}_{\textbf{3-out}}$, 3H, δ = 1.61-1.76 ppm,). Consequently, $i^{Ha}_{\textbf{3-out}}$ was determined using the following equation :

$$i^{Ha}_{\textbf{3-out}} = \left[\left(i^{Ha}_{\textbf{3-out}} + i^{Hc'}_{\textbf{3-out}} + i^{Hc'}_{\textbf{3-in}}\right) - \frac{i^{Ha}_{\textbf{3-in}}}{2}\right] \times \frac{6}{9} \qquad \text{(Normalization ratio } \frac{6}{9}\text{, } \textit{i.e.}\ \frac{6\ \text{Ha}}{6\ \text{Ha} + 3\ \text{Hc'}}\text{)}$$

Finally, the proportion of **3-in** was determined as follows:

$$\%\ \textbf{3-in} = \frac{i^{Ha}_{\textbf{3-in}}}{i^{Ha}_{\textbf{3-in}} + i^{Ha}_{\textbf{3-out}}} \times 100$$

SI.12.3 Time-dependent ^{1}H NMR spectra at variable pH

SI.12.3.1 Kinetics at pH 8.0

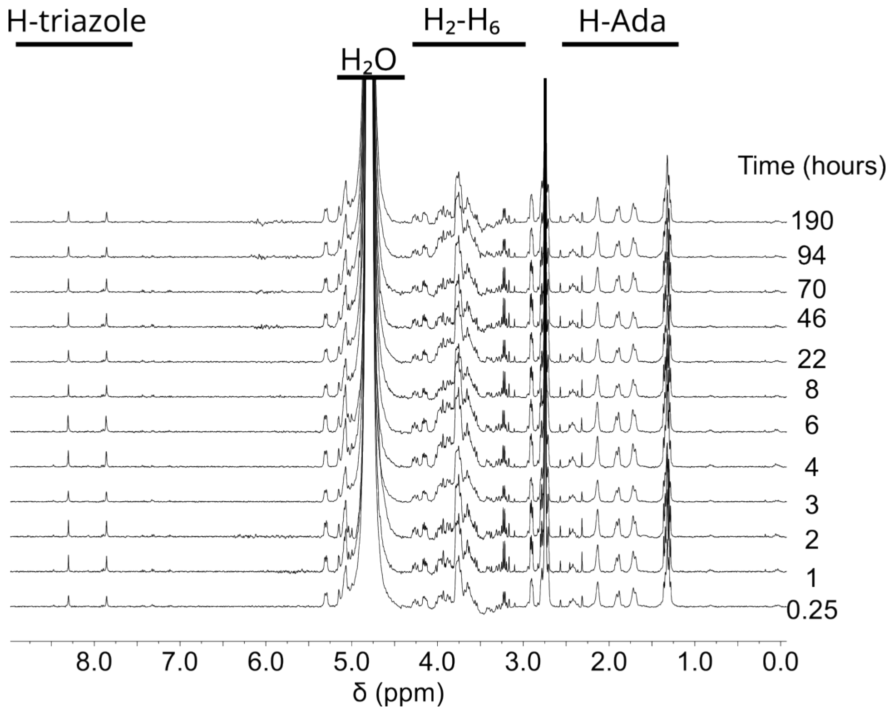

Figure S 33. Stack view of the ^{1}H NMR spectra (400 MHz, 300 K, D_2O) monitoring the time-dependent conversion of **3-out** into self-included monomer **3-in** at pH 8.0. The conversion is Instantaneous in these conditions.

SI.12.3.2 Kinetics at pH 5.6

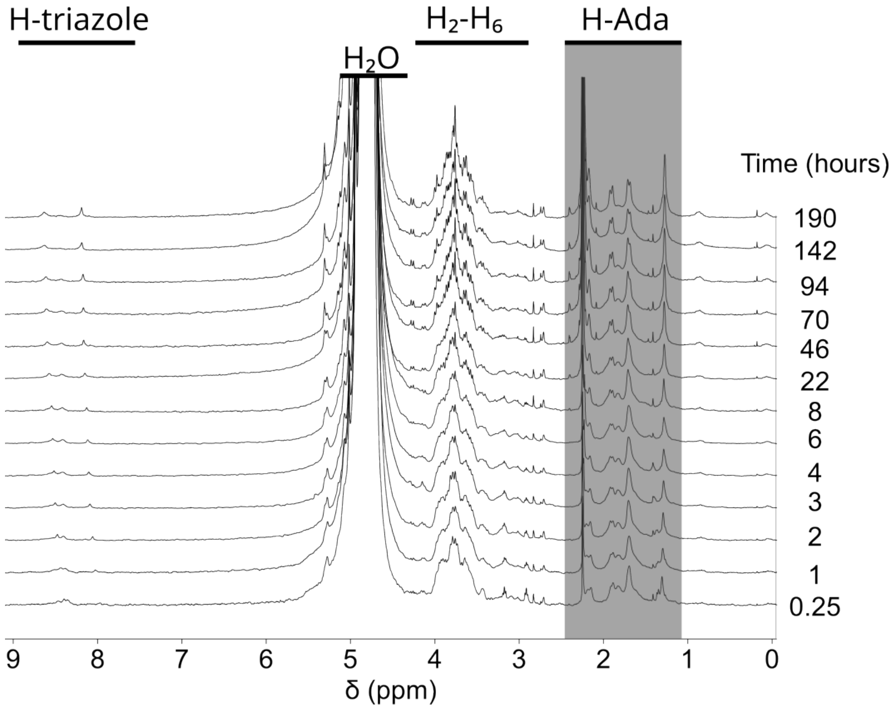

Figure S 34. Stack view of the ^1H NMR spectra (400 MHz, 300 K, D_2O) monitoring the time-dependent conversion of **3-out** into self-included monomer **3-in** at pH 5.6

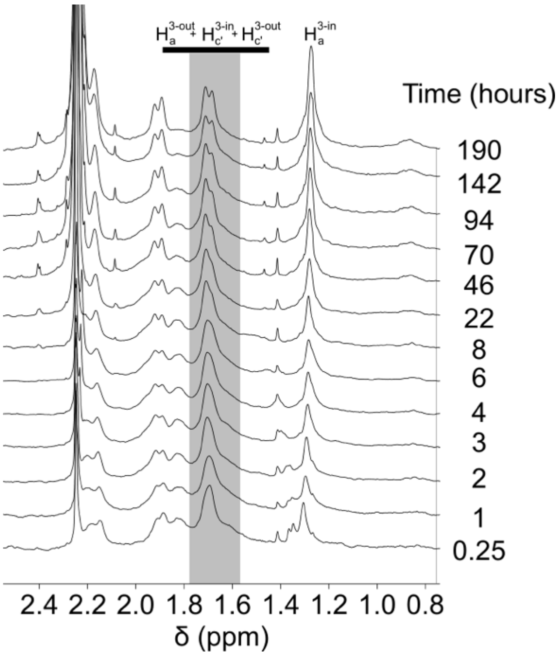

pH 5.6		
Time	% **3-in**	$i^{Ha}_{\textbf{3-out}} + i^{Hc'}_{\textbf{3-out}} + i^{Hc'}_{\textbf{3-in}}$
0.00	7.55[a]	–
0.25	20.00	20.00
1.00	21.05	19.00
2.00	25.48	15.70
3.00	33.11	12.08
4.00	35.37	11.31
6.00	36.13	11.07
8.00	38.80	10.31
22.00	49.26	8.12
46.00	59.00	6.78
70.00	70.30	5.69
94.00	76.92	5.20
142.00	90.09	4.44
190.00	97.56	4.10

[a] estimated at pH 2.0

Figure S 35. Zoom of the adamantane signals region

SI.12.3.3 Kinetics at pH 4.8

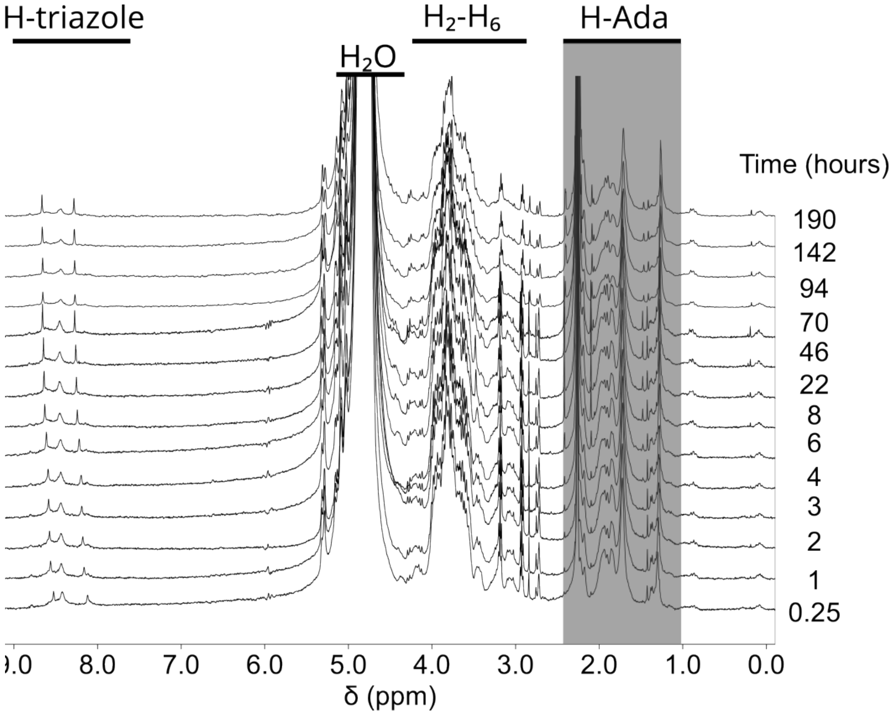

Figure S 36. Stack view of the ¹H NMR spectra (400 MHz, 300 K, D_2O) monitoring the time-dependent conversion of **3-out** into self-included monomer **3-in** at pH 4.8. Total conversion was not observed in the time frame of the experiment.

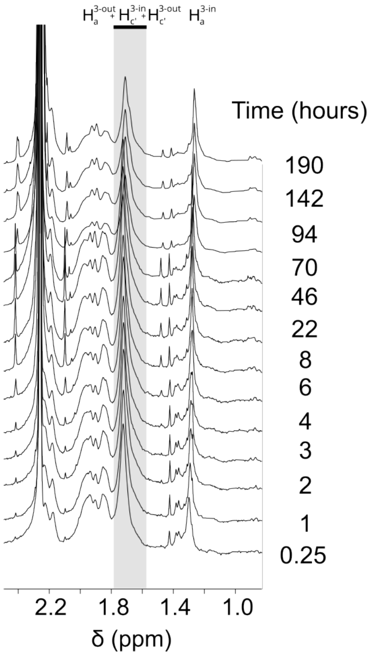

pH 4.8		
Time	% **3-in**	$i^{Ha}_{\textbf{3-out}} + i^{Hc'}_{\textbf{3-out}} + i^{Hc'}_{\textbf{3-in}}$
0.00	7.55[a]	–
0.25	13.25	30.19
1.00	14.47	27.65
2.00	16.01	24.98
3.00	17.32	23.09
4.00	17.73	22.56
6.00	18.27	21.89
8.00	19.34	20.68
22.00	23.77	16.83
46.00	26.56	15.06
70.00	31.08	12.87
94.00	34.75	11.51
142.00	37.88	10.56
190.00	38.06	10.51

[a] estimated at pH 2.0

Figure S 37.Zoom of the adamantane signals region

SI.12.3.4 Kinetics at pH 2.9

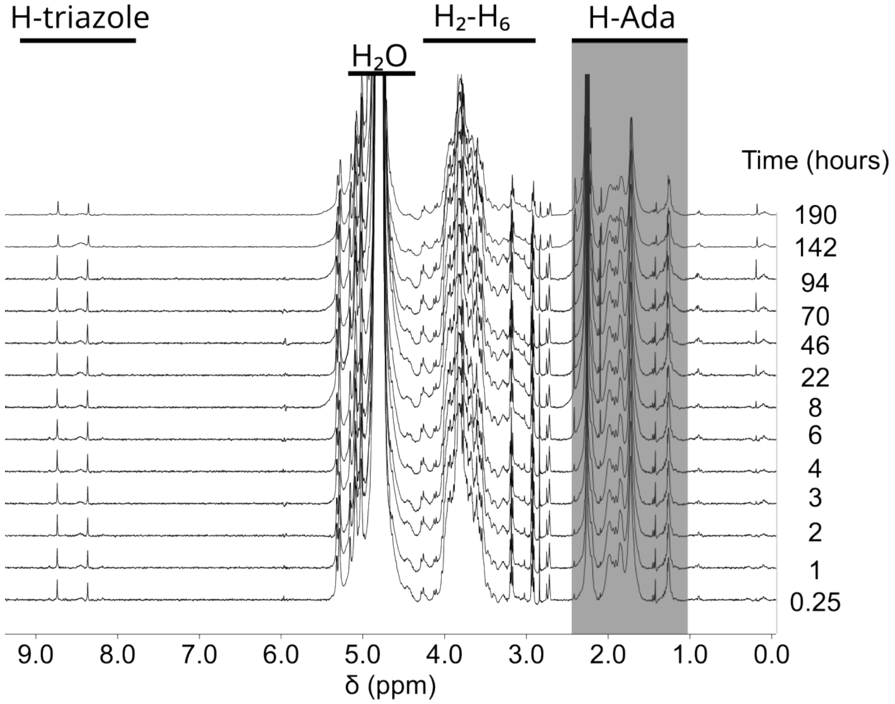

Figure S 38. Stack view of the ^1H NMR spectra (400 MHz, 300 K, D_2O) monitoring the time-dependent conversion of **3-out** into self-included monomer **3-in** at pH 2.9. Total conversion was not observed in the time frame of the experiment.

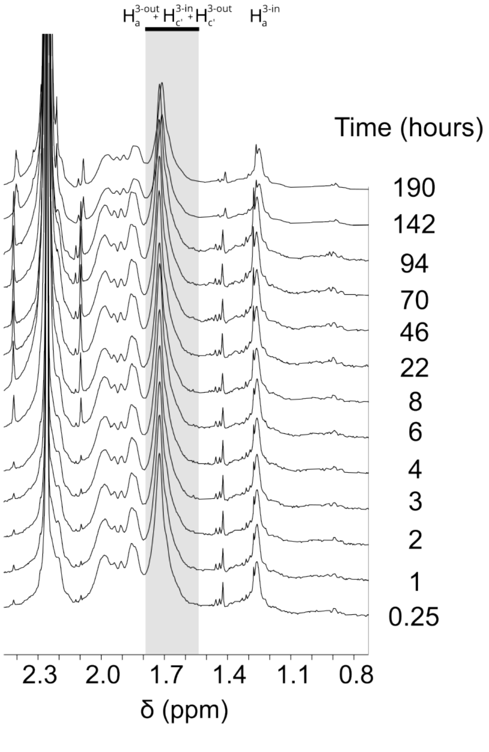

	pH 2.9	
Time	% **3-in**	$i^{Ha}_{\mathbf{3\text{-}out}} + i^{Hc'}_{\mathbf{3\text{-}out}} + i^{Hc'}_{\mathbf{3\text{-}in}}$
0.00	7.55[a]	–
2.00	11.36	35.21
3.00	11.64	34.36
4.00	13.14	30.44
6.00	13.84	28.90
8.00	14.10	28.36
22.00	14.46	27.67
46.00	15.35	26.06
70.00	14.92	26.81
94.00	15.27	26.20
142.00	16.39	24.41
190.00	16.91	23.66

[a] estimated at pH 2.0

Figure S 39.Zoom of the adamantane signals region

SI.12.3.5 Kinetics at pH 2.1

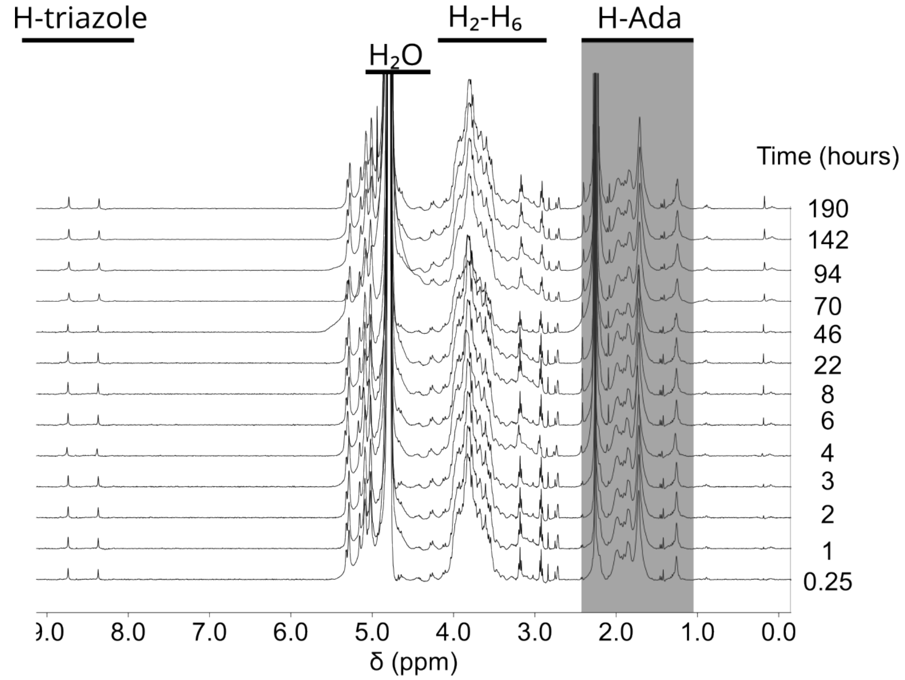

Figure S 40. Stack view of the ^{1}H NMR spectra (400 MHz, 300 K, D_2O) monitoring the time-dependent conversion of **3-out** into self-included monomer **3-in** at pH 2.1. Total conversion was not observed in the time frame of the experiment. No significant conversion occurs during the time frame of the experiment

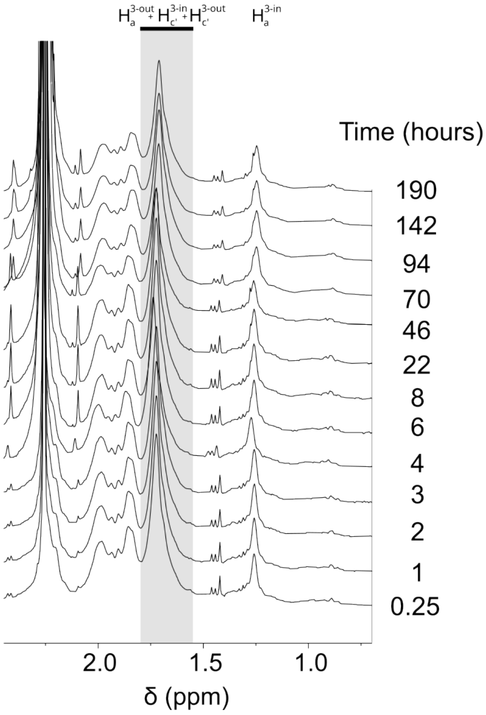

pH 2.1		
Time	% **3-in**	$i^{Ha}_{\mathbf{3\text{-}out}} + i^{Hc'}_{\mathbf{3\text{-}out}} + i^{Hc'}_{\mathbf{3\text{-}in}}$
0.00	7.55[a]	54.64
0.25	7.32	54.64
1.00	6.70	59.67
2.00	7.16	55.86
3.00	7.12	56.15
4.00	7.31	54.69
6.00	7.59	52.70
8.00	7.52	53.19
22.00	8.96	44.65
46.00	8.00	50.00
70.00	9.01	44,39
94.00	10.21	39.18
142.00	9.09	44.02
190.00	8.54	46.84

[a] estimated at pH 2.0

Figure S 41. Zoom of the adamantane signals region

SI.13 XYZ Coordinates (DFTB)

SI.13.1 **3-in**

C	5.10563612	1.04307604	3.73964500
H	6.19449806	1.16777599	3.79570389
C	3.15881801	2.35934091	4.09586382
H	2.78979993	2.13894391	3.08382702
O	4.57751179	2.28723788	4.10498285
C	4.63256598	-0.03929100	4.72167110
H	4.97210789	0.21558900	5.73329687
O	5.14796686	-1.30427599	4.42505121
C	3.09586811	-0.06967500	4.70325184
H	2.75814009	-0.33971101	3.69343090
O	2.63748002	-1.03172505	5.62654209
C	2.53226089	1.31749499	5.05190802
H	2.80332589	1.55747199	6.08875704
O	1.12506497	1.29210997	4.89764118
C	2.83334899	3.81450701	4.47477484
H	2.41495609	3.88032389	5.48090076
H	3.77868700	4.36612701	4.44140720
C	6.12192583	0.46188501	-1.40375805
H	6.83540487	0.50096601	-2.23963499
C	5.47035313	1.79777002	0.43952301
H	4.42504215	1.82823396	0.11073700
O	6.32777119	1.64647496	-0.69056797
C	6.42000103	-0.76493502	-0.52511901
H	7.46091318	-0.71584600	-0.17861199
O	6.26769495	-1.97067297	-1.21860194
C	5.48728895	-0.70938200	0.69162399
H	4.45047092	-0.78770500	0.33879501
O	5.76358414	-1.75811303	1.59490097
C	5.66331196	0.62634301	1.41803396
H	6.67621613	0.67382097	1.83976805
O	4.69934416	0.66169602	2.45659089
C	5.85456800	3.13597608	1.08162296
H	5.58202982	3.94142509	0.38773000
H	5.31612778	3.26292300	2.02200508
O	7.22103310	3.18040490	1.39655304
C	2.70700502	0.96729797	-5.38463116
H	2.53600597	1.29114294	-6.42010212
C	3.56460404	1.84799004	-3.35412693
H	2.64279103	1.59327197	-2.81542206
O	3.26935911	2.05948305	-4.73224878
C	3.68534088	-0.22714300	-5.37023592
H	4.62035608	0.08699400	-5.84977007
O	3.23862505	-1.34057605	-6.08443308
C	3.96261597	-0.56616199	-3.90241909
H	3.01564002	-0.84070998	-3.41212392

O	4.88076305	-1.62603605	-3.78931308
C	4.54888010	0.67218500	-3.21799397
H	5.48570204	0.93737102	-3.72663212
O	4.80843687	0.34210500	-1.86676204
C	4.12855816	3.16875792	-2.78184891
H	5.14550018	3.00269890	-2.41576505
H	4.15104580	3.88900805	-3.60501099
O	3.39316392	3.65289092	-1.69545996
C	-2.53758597	0.12149500	-5.41959810
H	-3.51161289	0.09594000	-5.92575216
C	-0.66873097	1.56534302	-5.23142719
H	-0.76934397	1.59096599	-4.13692188
O	-1.94827497	1.33147097	-5.80899000
C	-1.66650903	-1.05951095	-5.87552118
H	-1.53138196	-1.00552499	-6.96309519
O	-2.23624706	-2.30417395	-5.60057592
C	-0.29963899	-0.91943002	-5.19094419
H	-0.44089100	-0.95524901	-4.10157013
O	0.55059701	-1.96940506	-5.59091902
C	0.33155200	0.43309101	-5.55364990
H	0.57589400	0.43605599	-6.62424707
O	1.49432898	0.58778501	-4.76780319
C	-0.20849700	2.94798803	-5.73811388
H	0.71387100	2.87259793	-6.31533003
H	-1.00755000	3.35050988	-6.36666012
C	-5.64343309	0.20905800	-1.11170900
H	-6.66620111	0.33442199	-0.73307598
C	-4.19131184	1.34678698	-2.63096404
H	-3.36901808	1.42374802	-1.90488505
O	-5.43733978	1.31532705	-1.94671297
C	-5.53321600	-1.09919596	-1.91857898
H	-6.28893900	-1.08524406	-2.71553802
O	-5.75618982	-2.25412798	-1.16692400
C	-4.14119816	-1.14645398	-2.56341910
H	-3.37767196	-1.10652006	-1.77179396
O	-3.98267388	-2.32495689	-3.31744003
C	-3.98139405	0.07414100	-3.46951008
H	-4.73836088	0.02963100	-4.26406193
O	-2.68217707	0.04630200	-4.02834511
C	-4.21128607	2.59422493	-3.52155995
H	-3.41468000	2.51904297	-4.26480484
H	-5.18029499	2.63053799	-4.03655577
O	-3.97980189	3.77426004	-2.80259299
C	-4.52091789	0.73350400	4.03386879
H	-4.85302496	0.97409099	5.05440617
C	-4.60254002	1.66208303	1.86677396
H	-3.51114988	1.62530196	1.79751205
O	-4.98671007	1.78136504	3.23418307
C	-5.13188696	-0.60389298	3.58302212
H	-6.22460794	-0.55486602	3.67126894
O	-4.69957495	-1.67385197	4.37452793

C	-4.76222992	-0.82859099	2.10955501
H	-3.67077589	-0.93404901	2.03125906
O	-5.38975000	-1.99317396	1.61830604
C	-5.19189882	0.37390700	1.26333201
H	-6.28728819	0.44603300	1.26727104
O	-4.71885204	0.16795599	-0.05927900
C	-5.11235905	2.91830611	1.15446496
H	-4.55601978	3.77941489	1.54531598
H	-4.93211317	2.81808710	0.08302900
O	-6.49572420	3.08806205	1.31551301
C	0.30637100	1.72421896	5.96695280
H	0.91668302	2.19081497	6.75239515
C	-1.54006004	2.33390093	4.57344818
H	-1.02658904	2.07930207	3.63626909
O	-0.58279598	2.71386099	5.55718899
C	-0.46992600	0.52922702	6.56178904
H	-1.08668900	0.90694702	7.38734388
O	0.33820900	-0.46883300	7.10775709
C	-1.39097095	-0.02101500	5.46669912
H	-0.78333902	-0.34038001	4.60742903
O	-2.15262198	-1.11001396	5.93765020
C	-2.33959603	1.09680700	5.02545309
H	-2.97462702	1.36797404	5.88045597
O	-3.13364506	0.58001798	3.97442102
C	-2.46495008	3.55298901	4.33812714
H	-3.50758600	3.24673295	4.45050907
H	-2.21931100	4.29324293	5.10665989
O	-2.36314201	4.09357882	3.05429101
N	1.87587798	4.43370199	3.60959196
N	0.06176100	3.83441710	-4.64822578
H	5.20765591	-1.41677403	3.45793104
H	3.27927494	-1.75698304	5.61209917
H	5.63303518	-1.84788704	-1.94631696
H	5.96270609	-2.54015493	1.06213701
H	7.70420504	2.97900510	0.58551103
H	2.32470798	-1.55848098	-5.82108212
H	4.70246220	-2.23504591	-4.52036524
H	2.61847591	4.13995218	-2.04053998
H	-2.70141792	-2.27624798	-4.74350500
H	-0.00877500	-2.75128889	-5.70956421
H	-5.47929907	-2.11437511	-0.24135099
H	-4.47264481	-3.01839304	-2.85054302
H	-4.67626286	3.86547494	-2.14101100
H	-3.79797292	-1.49313998	4.69394398
H	-5.34913397	-2.65399504	2.32347488
H	-6.66858482	3.10102606	2.26474810
H	1.06877005	-0.68067598	6.49661303
H	-1.59824598	-1.58310902	6.57489395
H	-1.57375300	4.67243195	3.03063607
C	-0.80884397	4.34370279	-3.74445891
C	-0.00847900	4.95907116	-2.80742192

N	1.27499497	4.79835987	-3.20484400
N	1.30318701	4.12727404	-4.29407310
N	0.74059898	4.89462423	4.10384798
N	0.05971300	5.41351604	3.15196490
C	1.93792701	4.67429018	2.27670288
C	0.74744999	5.31165123	1.99109900
C	-0.38101500	5.65548992	-1.54868996
C	0.19426800	5.89815521	0.74105501
N	0.13152701	4.96226406	-0.37269101
C	-0.63541299	3.76749492	-0.03960500
C	-0.36041501	2.60987997	-1.01059103
C	-0.06385700	1.24139798	-0.37053299
C	1.29417002	1.28575802	0.33770099
C	-0.00547500	0.18881600	-1.48658097
C	0.33819500	-1.18269098	-0.90695697
C	1.63568795	-0.08522000	0.91476297
C	1.69861197	-1.11327600	-0.21423800
C	-0.73189700	-1.59540796	0.10372200
C	-1.14314795	0.81062698	0.62626600
C	0.56111097	-0.48754001	1.92349899
C	-0.79293197	-0.55434698	1.21972406
H	-1.87000597	4.24769497	-3.81524801
H	2.76257801	4.39340115	1.65740800
H	-1.47858596	5.76432514	-1.51481295
H	0.06784400	6.65252399	-1.57257497
H	-0.79943299	6.31036901	0.99163401
H	0.84394300	6.72534990	0.43501601
H	-1.71596706	3.98603296	-0.00307900
H	-0.33383599	3.47798109	0.96614599
H	0.51995999	2.87182093	-1.59726202
H	-1.20679605	2.50595403	-1.69401205
H	1.26685095	2.02396202	1.14220405
H	2.06676102	1.60096300	-0.36724001
H	0.74502802	0.47297600	-2.22920799
H	-0.97386700	0.14101200	-1.99440801
H	0.37811100	-1.92077696	-1.71760201
H	2.60836697	-0.02864900	1.41558599
H	2.46704507	-0.82155502	-0.93484199
H	1.96272695	-2.09543490	0.18662401
H	-0.49425501	-2.57792211	0.51886100
H	-1.70439005	-1.66835201	-0.38945499
H	-2.11184692	0.75186098	0.12224800
H	-1.23132896	1.54370999	1.42991102
H	0.52573103	0.24215400	2.73607302
H	0.79841900	-1.46324301	2.35669708
H	-1.56130695	-0.83471000	1.94954300
C	-2.07649302	36.13583755	-2.05108905
H	-1.09602499	36.05981827	-2.50391102
H	-2.30552602	35.21416855	-1.53280199
H	-2.08512306	36.95883179	-1.34878802
H	-2.81507993	36.31058502	-2.82243109

SI.13.2 **3-TS**

C	4.89230585	1.40693796	3.76134610
H	5.94757414	1.67880797	3.90176296
C	2.76993108	2.45561099	3.99680495
H	2.42868996	2.10631990	3.01349592
O	4.18345308	2.58783698	4.01299810
C	4.51498890	0.29452100	4.77126884
H	4.78871584	0.64039898	5.77780581
O	5.22573090	-0.88530099	4.54879904
C	2.99233198	0.04518900	4.74049902
H	2.69426608	-0.24164300	3.72276998
O	2.63748002	-1.03172505	5.62654209
C	2.33033800	1.40791702	5.07280397
H	2.69795990	1.75084496	6.05081606
O	0.92720503	1.28752601	5.09615993
C	2.20235491	3.84721994	4.30812502
H	1.19313896	3.74546695	4.71732712
H	2.84613204	4.29455423	5.07497978
C	6.39163494	0.67816800	-1.27003396
H	7.15415001	0.73420501	-2.06246305
C	5.58921623	2.07303691	0.49692500
H	4.55554581	2.09375191	0.13299200
O	6.48047209	1.90046299	-0.60215300
C	6.71296120	-0.47972101	-0.30927399
H	7.72832680	-0.35704899	0.08719500
O	6.66545296	-1.72026896	-0.95508498
C	5.70893192	-0.42605400	0.84969997
H	4.69829512	-0.58706403	0.44621900
O	6.00373983	-1.41312897	1.81485701
C	5.73967886	0.94283903	1.53246903
H	6.69651890	1.06351304	2.05806899
O	4.66195011	0.94382399	2.46155310
C	5.96555614	3.43691111	1.09812403
H	5.67561388	4.21259689	0.37878299
H	5.44494915	3.58802295	2.04527211
O	7.33598900	3.50638390	1.39224696
C	2.83141303	1.16476500	-5.15540504
H	2.55981207	1.48502195	-6.17159081
C	4.02042580	2.00493193	-3.27522898
H	3.14782810	1.87921500	-2.62225199
O	3.56279397	2.21671104	-4.61251211
C	3.71872997	-0.09185400	-5.22336102
H	4.62159920	0.14856200	-5.79754591
O	3.12366009	-1.17747402	-5.87124014
C	4.10698986	-0.45196101	-3.78581405
H	3.19000006	-0.61285400	-3.19805288
O	4.88076305	-1.62603605	-3.78931308
C	4.87598705	0.72501397	-3.16577196
H	5.82461023	0.85462099	-3.70587301

O	5.13176012	0.40223500	-1.80844903
C	4.77344418	3.29073501	-2.86819410
H	5.83749580	3.08891797	-2.72871399
H	4.65735579	3.99751306	-3.70242906
O	4.32391310	3.84658194	-1.67005706
C	-2.42079091	0.40337199	-5.06475115
H	-3.31575704	0.33287299	-5.69807196
C	-0.54262000	1.84866297	-4.79862213
H	-0.70990199	1.91354299	-3.71705508
O	-1.77948105	1.58377004	-5.46030712
C	-1.49687696	-0.80383497	-5.33503485
H	-1.25672805	-0.80962700	-6.40669823
O	-2.05803990	-2.05152798	-5.06859398
C	-0.19644199	-0.59575999	-4.54474783
H	-0.42466000	-0.48191601	-3.47498012
O	0.65319502	-1.70538795	-4.72341585
C	0.45940799	0.69710201	-5.05582809
H	0.62385398	0.60094398	-6.13917208
O	1.68031394	0.89271700	-4.38705683
C	-0.07238500	3.21567202	-5.32473516
H	0.59240597	3.07767606	-6.18407393
H	-0.96170002	3.75854397	-5.65473509
C	-5.87871504	0.19824800	-0.98103499
H	-6.89136124	0.13895100	-0.55767202
C	-4.75425911	1.47023702	-2.71307993
H	-3.98624492	1.97385001	-2.12571001
O	-5.89799023	1.27279496	-1.86764002
C	-5.56586123	-1.05305898	-1.81408405
H	-6.25177813	-1.06583202	-2.67057896
O	-5.75618982	-2.25412798	-1.16692400
C	-4.12258720	-0.92579901	-2.30318904
H	-3.47002912	-0.65968901	-1.45986497
O	-3.68655705	-2.14186192	-2.86379910
C	-4.11494303	0.20246100	-3.32906604
H	-4.69102383	-0.12536600	-4.20587587
O	-2.77519798	0.47365400	-3.70785594
C	-5.23729181	2.40877104	-3.82539201
H	-4.40592384	2.63504791	-4.49646091
H	-6.03423309	1.89988995	-4.38984489
O	-5.69484520	3.62582588	-3.31352496
C	-4.21846294	-0.09082300	4.12319088
H	-4.43305779	-0.38285100	5.16053200
C	-5.32174683	1.05220497	2.34246302
H	-4.48450804	1.74254894	2.19877791
O	-5.35433006	0.60936701	3.69882703
C	-4.05734921	-1.35629404	3.26087904
H	-4.96305513	-1.96971297	3.41349196
O	-2.91614699	-2.10800505	3.57696295
C	-3.96683407	-0.97669202	1.77549696
H	-3.04451108	-0.39527601	1.63527000
O	-3.91910005	-2.14661098	0.98556799

C	-5.16494417	-0.11809600	1.34605002
H	-6.07457781	-0.73194402	1.35996401
O	-4.92684984	0.40276101	0.04387900
C	-6.65380001	1.77457404	2.11140609
H	-6.71352100	2.63186312	2.79374290
H	-6.70135784	2.12906909	1.08099902
O	-7.74193096	0.91012299	2.29993606
C	0.23083900	1.66055202	6.26352215
H	0.91696799	2.06605601	7.02021980
C	-1.69446003	2.36458206	5.04957724
H	-1.22805703	2.27865410	4.06558800
O	-0.68768901	2.67851806	6.01277399
C	-0.50830698	0.43046999	6.81193590
H	-1.10498202	0.73675299	7.67980194
O	0.34002200	-0.58734798	7.25229502
C	-1.43507099	-0.06636900	5.69502592
H	-0.81843501	-0.30994800	4.81687212
O	-2.13105512	-1.22175705	6.12471914
C	-2.42944789	1.03412795	5.29946995
H	-3.17269492	1.14646900	6.10126781
O	-3.06670094	0.71095997	4.07143402
C	-2.67723608	3.54529691	5.04932594
H	-3.59232092	3.24034405	4.53745604
H	-2.91140509	3.79510903	6.09383488
O	-2.19903708	4.65919304	4.35711384
N	2.09159207	4.79520321	3.21572804
N	0.60114902	3.98699498	-4.31445694
H	5.34047318	-1.03007400	3.59044409
H	3.43585396	-1.57222700	5.70945406
H	5.96686888	-1.68851995	-1.62941504
H	6.30710411	-2.19571090	1.33487201
H	7.80670977	3.32089996	0.57042599
H	2.27608800	-1.38521600	-5.42890882
H	4.56899786	-2.15897298	-4.53544521
H	3.36741900	4.04855585	-1.78188205
H	-2.52940392	-2.04156494	-4.21244001
H	0.07649200	-2.47471499	-4.85552216
H	-5.18166494	-2.28684402	-0.37525901
H	-4.23588419	-2.84143090	-2.47217488
H	-6.31897211	3.41865706	-2.60675597
H	-2.66047907	-1.93336403	4.50037479
H	-3.30682206	-2.75026989	1.42851603
H	-7.66187382	0.54293799	3.18924809
H	1.05859303	-0.72928500	6.60603905
H	-1.52395499	-1.69488394	6.71572208
H	-1.37721205	4.96510315	4.75982904
C	0.59946001	5.33352995	-4.12506199
C	1.39754105	5.53720379	-3.01615191
N	1.82751095	4.31644106	-2.62759304
N	1.34721804	3.41222000	-3.39046001
N	1.26733100	5.82857800	3.40754795

N	1.33404195	6.61240387	2.40195394
C	2.71375203	4.94002819	2.02208591
C	2.20254111	6.11764002	1.49082303
C	1.76581097	6.74939823	-2.20704007
C	2.45845795	6.79869080	0.17276300
N	1.40641999	6.56653023	-0.81163102
C	0.10029400	7.09290504	-0.46549001
C	-1.04657197	6.24799490	-1.03473198
C	-1.17035604	4.81738615	-0.47239900
C	0.17824499	4.27253199	0.00072400
C	-1.71325397	3.87674093	-1.55736494
C	-1.78780603	2.44487500	-1.03005099
C	0.09158400	2.84697604	0.51897299
C	-0.41461200	1.94311404	-0.59331298
C	-2.74317908	2.41490793	0.16030900
C	-2.12351108	4.76876402	0.72605997
C	-0.85824603	2.82830501	1.70665896
C	-2.22370410	3.33805990	1.25649905
H	0.05381800	5.99977779	-4.75900984
H	3.41299391	4.23653603	1.62917101
H	1.26991296	7.63087177	-2.64241910
H	2.84720802	6.89186811	-2.29327989
H	2.58919501	7.87676477	0.37534899
H	3.38801003	6.39497709	-0.23346899
H	-0.01939600	8.12491131	-0.84568697
H	0.02220400	7.13361883	0.62097800
H	-0.92914099	6.19580317	-2.11818194
H	-1.98083997	6.78058195	-0.84225202
H	0.51188099	4.90576792	0.80816197
H	0.91298300	4.31207800	-0.79446799
H	-1.06171405	3.90849710	-2.42958498
H	-2.70669508	4.20439386	-1.87699604
H	-2.14354300	1.77975202	-1.82275498
H	1.09179604	2.52196693	0.83442700
H	0.28428900	1.96422994	-1.43356895
H	-0.49419099	0.90976697	-0.24426900
H	-2.81945801	1.39611995	0.53755200
H	-3.74397302	2.72951388	-0.14120400
H	-3.11795902	5.11088991	0.42778599
H	-1.76499295	5.42655516	1.51946998
H	-0.46670800	3.48027492	2.48985291
H	-0.95381498	1.81141305	2.09785008
H	-2.90879202	3.33009911	2.11006308
C	-2.07649302	36.13583755	-2.05108905
H	-1.48918998	35.54866409	-2.74611092
H	-2.04105806	35.69462585	-1.06733501
H	-1.71031201	37.15197372	-2.03170109
H	-3.10500002	36.14296341	-2.40195799

SI.13.3 **3-out**

C	4.61830091	1.42609596	3.56446195
H	5.63620281	1.83083606	3.62831092
C	2.39191508	2.20716596	3.79502106
H	2.11591506	1.71941900	2.84953499
O	3.77644610	2.52625203	3.78141904
C	4.41891813	0.32273400	4.63022184
H	4.70476198	0.71165299	5.61512899
O	5.22544003	-0.79109299	4.39149094
C	2.93016005	-0.05636100	4.65170383
H	2.64768505	-0.43939301	3.65921593
O	2.63748002	-1.03172505	5.62654209
C	2.12139010	1.21286201	4.94083786
H	2.46176100	1.65399599	5.88786077
O	0.76761502	0.84470803	5.02707291
C	1.60552001	3.51918507	3.94306588
H	0.74034202	3.36495709	4.59252977
H	2.27085590	4.27042723	4.37466478
C	6.13514900	0.71337599	-1.41908002
H	6.90987206	0.80257601	-2.19411898
C	5.20758390	2.01601100	0.32473800
H	4.20004082	1.93152905	-0.10135200
O	6.18160200	1.92138898	-0.71217901
C	6.44178104	-0.47688001	-0.49431199
H	7.44380379	-0.36000699	-0.06287100
O	6.42895794	-1.69257605	-1.18456900
C	5.40569496	-0.47171900	0.63964498
H	4.40718794	-0.63381702	0.20729500
O	5.68831110	-1.47956002	1.58415306
C	5.41629505	0.88558602	1.34737206
H	6.38751411	1.02444696	1.84121597
O	4.37736607	0.85095000	2.30868697
C	5.38972187	3.40243101	0.95377398
H	5.14073181	4.15890789	0.19787499
H	4.72133017	3.50524092	1.80978894
O	6.69325018	3.58189106	1.43934906
C	2.78048801	0.98110503	-5.48554087
H	2.56881094	1.24480104	-6.53077602
C	3.76551294	1.96217704	-3.56596088
H	2.85965705	1.82404101	-2.96087790
O	3.41391110	2.08889198	-4.93886805
C	3.74007702	-0.23305400	-5.43644905
H	4.66871881	0.06060700	-5.94138098
O	3.28380489	-1.36887395	-6.10617685
C	4.02831984	-0.51310998	-3.95783091
H	3.07608795	-0.70292097	-3.43944693
O	4.88076305	-1.62603605	-3.78931308
C	4.67883396	0.73663300	-3.35200810
H	5.64366293	0.90962499	-3.84834599

O	4.87979507	0.47224399	-1.97649205
C	4.45404482	3.27954888	-3.15882993
H	5.18793297	3.07741809	-2.37606311
H	4.96876287	3.66856289	-4.04598284
O	3.57471704	4.22344017	-2.62411094
C	-2.38735199	-0.25305399	-5.41009521
H	-3.32495308	-0.45309499	-5.94515705
C	-0.66939998	1.37518096	-5.37310410
H	-0.83173400	1.54930997	-4.29978895
O	-1.88559401	0.93283898	-5.96577501
C	-1.38515902	-1.39518404	-5.62678003
H	-1.17954099	-1.49479401	-6.70080423
O	-1.84319901	-2.63692904	-5.18010807
C	-0.08605600	-1.00523400	-4.90389681
H	-0.30209199	-0.84105200	-3.83842707
O	0.85432899	-2.04707289	-5.03213787
C	0.44383600	0.30859599	-5.50074005
H	0.68657601	0.15049900	-6.56009388
O	1.58299601	0.72428101	-4.77954912
C	-0.32959399	2.70660305	-6.06315279
H	0.46479201	2.57796693	-6.80036211
H	-1.23977399	3.04176903	-6.57063007
C	-5.58170986	0.21305500	-1.19360995
H	-6.59583092	0.35879800	-0.79686397
C	-4.11972380	1.25867605	-2.75908494
H	-3.30010891	1.35966599	-2.03518200
O	-5.37002993	1.27263796	-2.07624888
C	-5.50927877	-1.13147902	-1.95524299
H	-6.28367615	-1.12438202	-2.73221493
O	-5.75618982	-2.25412798	-1.16692400
C	-4.12885618	-1.23896396	-2.60311198
H	-3.36382294	-1.20904601	-1.81185496
O	-4.00920820	-2.44030499	-3.33481908
C	-3.91712594	-0.05177300	-3.53893805
H	-4.64006519	-0.10954400	-4.36340284
O	-2.59378409	-0.12135500	-4.03543997
C	-4.12605810	2.45966196	-3.71233606
H	-3.39242697	2.30078506	-4.50552893
H	-5.12788200	2.53098392	-4.15855217
O	-3.77409101	3.65825200	-3.08091497
C	-4.38903904	-0.44260201	3.88396811
H	-4.64293909	-0.71326602	4.91723919
C	-5.23246002	0.87427598	2.11212397
H	-4.35467005	1.53147697	2.10722709
O	-5.44064188	0.35747001	3.42908907
C	-4.28189182	-1.69568300	3.00404310
H	-5.26331711	-2.19564199	2.97666192
O	-3.31718707	-2.61589098	3.44383788
C	-3.92346311	-1.21163595	1.59321499
H	-2.97736001	-0.65606499	1.63843405
O	-3.77585912	-2.31682897	0.72339100

C	-5.02119923	-0.26638201	1.09377503
H	-5.96007681	-0.82587200	0.99248803
O	-4.62705421	0.27025801	-0.15864500
C	-6.49132586	1.68081498	1.77815795
H	-6.61281109	2.46116495	2.53897500
H	-6.37802076	2.14882708	0.80040997
O	-7.63405085	0.86650503	1.71451104
C	0.00699700	1.27797699	6.12245321
H	0.64089102	1.71147895	6.90818977
C	-1.73343503	1.89871097	4.67166424
H	-1.11349797	1.65219200	3.79847002
O	-0.88893402	2.28628111	5.75157499
C	-0.74156398	0.04529000	6.65618420
H	-1.37232602	0.33694100	7.50368786
O	0.12618101	-0.94997501	7.11523294
C	-1.61802304	-0.47248301	5.51113987
H	-0.96004403	-0.76748198	4.67951918
O	-2.38103199	-1.58874404	5.92756414
C	-2.55014300	0.64129502	5.01155996
H	-3.29730606	0.86432701	5.78485107
O	-3.16457796	0.23158599	3.81178594
C	-2.61311793	3.10481191	4.29935217
H	-3.55515409	3.07364988	4.85145092
H	-2.06404996	4.00904179	4.59431887
O	-2.94443107	3.12635112	2.94683909
N	1.10518396	3.98361206	2.68155503
N	0.11458800	3.71655989	-5.15123177
H	5.23466778	-0.99455398	3.43725491
H	3.39919400	-1.62759602	5.66389608
H	5.77062607	-1.64941096	-1.90077198
H	5.99638796	-2.25064397	1.08822298
H	7.29596376	3.43794703	0.69927901
H	2.42630911	-1.64292395	-5.72464085
H	4.70179701	-2.22746491	-4.52638721
H	2.98583794	4.55339622	-3.33357692
H	-2.46613002	-2.51684093	-4.43882608
H	0.34389800	-2.87113404	-5.06517792
H	-5.06177521	-2.31925893	-0.47936001
H	-4.50870419	-3.11296105	-2.84829593
H	-4.34306097	3.76933694	-2.30785608
H	-2.95314908	-2.31809497	4.29548979
H	-3.37242198	-3.02083302	1.25102496
H	-7.71094894	0.41982499	2.56640792
H	0.89046502	-1.01354098	6.51293612
H	-1.81620300	-2.10124397	6.52477407
H	-2.13315606	3.26278305	2.42900705
C	-0.59067899	4.37778282	-4.20310020
C	0.31467399	5.26816893	-3.66264296
N	1.49124503	5.08331203	-4.30367708
N	1.35909700	4.16509581	-5.18796206
N	-0.13884000	3.72397709	2.29412794

N	-0.32227701	4.21005201	1.12081504
C	1.74601495	4.66436577	1.70639503
C	0.80962199	4.80817509	0.69691098
C	0.10727000	6.34005880	-2.64777303
C	1.00355506	5.49865818	-0.61136299
N	-0.21681300	5.80279684	-1.33294106
C	-1.13459098	6.65651417	-0.59471399
C	-2.51781893	6.66320992	-1.26016700
C	-3.71776199	6.57442284	-0.30236000
C	-3.73261404	5.22446108	0.42002800
C	-5.00484896	6.70081186	-1.12971902
C	-6.23428297	6.58356810	-0.23037700
C	-4.95833778	5.11684704	1.32287097
C	-6.22750807	5.23178911	0.48181501
C	-6.20845985	7.70659018	0.80494702
C	-3.70723104	7.69954395	0.73669797
C	-4.92537689	6.24020386	2.35727906
C	-4.93555212	7.58954477	1.64140904
H	-1.62005305	4.18959379	-3.98412704
H	2.76171303	4.98332691	1.79498196
H	-0.72890300	6.95488024	-2.98494101
H	1.01096797	6.97084904	-2.62425494
H	1.59972799	6.41377211	-0.41501099
H	1.61845505	4.85517120	-1.25136900
H	-0.74393803	7.68551302	-0.49813801
H	-1.21439898	6.23174191	0.40314299
H	-2.56353402	5.79964876	-1.92589498
H	-2.63348699	7.56496382	-1.86720204
H	-2.83375907	5.10277081	1.02146196
H	-3.73760605	4.41419983	-0.31073001
H	-5.02729082	5.92448997	-1.89773202
H	-5.01970291	7.66778898	-1.64000201
H	-7.14075089	6.67185783	-0.84128201
H	-4.93142986	4.15249681	1.83679104
H	-6.26486921	4.42382383	-0.25314999
H	-7.11100483	5.14241982	1.11788702
H	-7.08616877	7.63794804	1.45199096
H	-6.23811388	8.67689037	0.30278099
H	-3.70578599	8.66875076	0.23104601
H	-2.80363703	7.63911295	1.34546804
H	-4.02649784	6.14529896	2.96934295
H	-5.79131699	6.16481018	3.01919103
H	-4.91216087	8.39847565	2.38171506
C	-2.07649302	36.13583755	-2.05108905
H	-2.08381200	35.93064880	-3.11372495
H	-3.02955794	36.56044388	-1.76193798
H	-1.91402698	35.21470261	-1.50692904
H	-1.28514397	36.83718872	-1.82292902

SI.13.4 **3-in•H⁺**

C	5.03854704	0.73678136	3.83540344
H	6.12986612	0.73795021	3.94129157
C	3.23639083	2.25900173	4.09030390
H	2.88984990	2.03787398	3.06918550
O	4.63511038	2.04151154	4.16257715
C	4.39718151	-0.28411585	4.78818798
H	4.72995710	-0.08847131	5.81428385
O	4.76491261	-1.59534693	4.48025703
C	2.86824107	-0.12861270	4.71110058
H	2.54913807	-0.35397357	3.68421531
O	2.24761581	-1.02847302	5.59686375
C	2.45773792	1.31743264	5.03932714
H	2.71355247	1.53923142	6.08240843
O	1.06977057	1.46974993	4.81128454
C	3.02828526	3.75272703	4.40269470
H	2.46940780	3.88935447	5.32940292
H	4.01997566	4.20204258	4.50639200
C	5.99151707	0.37217131	-1.33866513
H	6.67832613	0.39989313	-2.19443941
C	5.62915659	1.59382558	0.67317808
H	4.57982588	1.83286548	0.44331038
O	6.37096882	1.45139241	-0.53223014
C	6.14764977	-0.96011078	-0.59121674
H	7.19714785	-1.07021117	-0.28302124
O	5.80831194	-2.06474233	-1.37995553
C	5.26421165	-0.90807831	0.66408467
H	4.21924067	-0.81174773	0.33807048
O	5.41129446	-2.05724597	1.46012235
C	5.63089180	0.30954507	1.51717770
H	6.62478256	0.15575536	1.95543730
O	4.64684057	0.39020401	2.53576660
C	6.29401922	2.75768447	1.42591262
H	6.24011850	3.65666842	0.79432911
H	5.77377272	2.93727136	2.36872888
O	7.61725283	2.46224213	1.75882375
C	2.50880289	1.58533716	-5.14379883
H	2.31154633	2.07769704	-6.10445595
C	3.41900325	2.14828968	-3.02488375
H	2.52447462	1.79480839	-2.49483156
O	3.05235672	2.57617855	-4.32852840
C	3.54429865	0.45097911	-5.33488131
H	4.44694853	0.91266638	-5.75489044
O	3.16634345	-0.53678912	-6.23911285
C	3.87333465	-0.13147223	-3.95312667
H	2.94809508	-0.52623570	-3.50262499
O	4.83378506	-1.15540242	-4.03430128
C	4.42943573	0.99168110	-3.06931138
H	5.36933613	1.34920323	-3.50785851

O	4.65698910	0.49444491	-1.76210201
C	4.00767231	3.37023449	-2.31318998
H	4.38868713	3.07436633	-1.33573520
H	4.83244801	3.76064014	-2.91764021
O	3.05065513	4.38147688	-2.09594727
C	-2.52108145	-0.18249479	-5.33217430
H	-3.45185518	-0.43007091	-5.85664082
C	-0.96582210	1.60418618	-5.23144865
H	-1.12038445	1.71664608	-4.14707994
O	-2.14693356	1.08244097	-5.81696558
C	-1.42584085	-1.20397758	-5.65855742
H	-1.25407374	-1.21135199	-6.74304438
O	-1.74981904	-2.50604367	-5.27246952
C	-0.14169320	-0.74875396	-4.94472933
H	-0.34152460	-0.72610313	-3.86616468
O	0.89801323	-1.66434944	-5.20099926
C	0.25669706	0.66847688	-5.39562798
H	0.58062983	0.64057308	-6.44218111
O	1.29899204	1.14091480	-4.55344725
C	-0.78313947	2.99832964	-5.86584759
H	-0.10113186	2.96091485	-6.71624947
H	-1.76780391	3.32495642	-6.21115923
C	-5.70068645	-0.16559677	-1.07815969
H	-6.72561550	-0.05745931	-0.70288914
C	-4.21445322	1.03551495	-2.49192119
H	-3.41642880	1.01617932	-1.73412728
O	-5.47836876	0.98377627	-1.85230196
C	-5.58047676	-1.44533253	-1.92932248
H	-6.35083485	-1.43079877	-2.71043849
O	-5.77898169	-2.60592270	-1.18120337
C	-4.19716167	-1.45919466	-2.60055327
H	-3.42477417	-1.49313128	-1.81649864
O	-4.04846191	-2.56713390	-3.45564055
C	-4.01370716	-0.17657588	-3.41617656
H	-4.75319862	-0.15274429	-4.22536087
O	-2.69685102	-0.16385356	-3.94399428
C	-4.14829540	2.35974193	-3.26279402
H	-3.48685455	2.24972677	-4.12318945
H	-5.15617895	2.60272717	-3.61507821
O	-3.61677146	3.40753412	-2.48371911
C	-4.50866270	0.46302640	4.03256464
H	-4.83477497	0.69102842	5.05639410
C	-4.83928299	1.30813575	1.83831775
H	-3.76397729	1.44201958	1.66873240
O	-5.12306643	1.43104231	3.22935462
C	-4.96941853	-0.95131069	3.64174771
H	-6.05776405	-1.01899087	3.77435088
O	-4.38444233	-1.94016409	4.43886471
C	-4.64320993	-1.18647444	2.15763330
H	-3.55189681	-1.17476106	2.03001523
O	-5.15599966	-2.41874003	1.70951426

C	-5.25228882	-0.07248309	1.30237985
H	-6.34581804	-0.15968959	1.33220661
O	-4.78001118	-0.25817525	-0.02389834
C	-5.62609959	2.43112779	1.14825082
H	-5.22822475	3.39711261	1.48935652
H	-5.50740623	2.34642124	0.06660026
O	-6.99769735	2.33356595	1.40121174
C	0.24176262	1.99393690	5.83760834
H	0.83811152	2.57060003	6.55720997
C	-1.56636786	2.33636665	4.33536291
H	-1.00054407	1.93841434	3.48510909
O	-0.66054428	2.89605832	5.28206396
C	-0.52226251	0.87652236	6.58457565
H	-1.15502572	1.36140370	7.33839464
O	0.29523021	-0.00900847	7.28296328
C	-1.41563690	0.15315680	5.56909704
H	-0.78138304	-0.27850965	4.77895737
O	-2.17344022	-0.86575019	6.17513990
C	-2.37294888	1.17111945	4.93810129
H	-3.04776049	1.55140495	5.71503592
O	-3.11214089	0.50525576	3.92715931
C	-2.48232436	3.46872377	3.84190869
H	-3.43741703	3.04444695	3.52854347
H	-2.65071297	4.15848017	4.67529440
O	-1.96396148	4.13666725	2.72071290
N	2.28040123	4.43764162	3.38904023
N	-0.25443313	3.93868136	-4.92508030
H	4.82711220	-1.70387983	3.51494360
H	2.80925679	-1.81686938	5.64196110
H	5.33807135	-1.77643609	-2.17934632
H	5.50633860	-2.81067705	0.86146057
H	8.09585190	2.26565218	0.94375855
H	2.37065887	-0.99950016	-5.90843391
H	4.68959188	-1.62746978	-4.86752176
H	2.73193073	4.67649603	-2.97988725
H	-2.41368341	-2.49449396	-4.55942154
H	0.48222807	-2.53395939	-5.30757666
H	-5.43151140	-2.49126363	-0.27776068
H	-4.50904846	-3.31149626	-3.04183769
H	-4.23668718	3.59163451	-1.76800978
H	-3.56090522	-1.60633910	4.83260679
H	-5.04271221	-3.05639911	2.42763853
H	-7.12176847	2.33251572	2.35861397
H	0.94623542	-0.41143978	6.67685795
H	-1.64134455	-1.23558187	6.89440155
H	-1.22349989	4.69283915	3.00948334
C	-0.84658188	4.41332388	-3.80723405
C	0.14559275	5.14521456	-3.18760228
N	1.24625945	5.08218193	-3.97687316
N	0.99357063	4.36268759	-4.99881840
N	1.07394910	4.90072918	3.65522647

N	0.60011363	5.45964813	2.61334705
C	2.60830164	4.71061850	2.10490680
C	1.50448370	5.38031149	1.60772622
C	0.17793573	5.89039516	-1.89939916
C	1.19489491	6.02539444	0.30028090
N	0.75326526	5.08970547	-0.77726156
C	-0.18917981	4.05374908	-0.28288284
C	-0.22539960	2.80869365	-1.17155313
C	-0.03721535	1.47405970	-0.42385823
C	1.32974112	1.39955580	0.26609087
C	-0.12974255	0.34896889	-1.46241009
C	0.04104683	-1.01296103	-0.79228795
C	1.51104569	0.03046889	0.92256808
C	1.41790593	-1.06993198	-0.13275760
C	-1.05248451	-1.20773029	0.25844181
C	-1.13982189	1.25772643	0.61391580
C	0.42036575	-0.16980341	1.97098565
C	-0.94885296	-0.09587657	1.30042231
H	-1.86538887	4.22168970	-3.54354906
H	3.54679918	4.43713522	1.67157245
H	-0.82790607	6.20669317	-1.61663532
H	0.81062788	6.76950836	-2.04164624
H	0.38040048	6.72868109	0.49346995
H	2.06928515	6.56537390	-0.06865894
H	-1.17850697	4.50184870	-0.18415765
H	0.12672846	3.77428365	0.72014344
H	0.56387126	2.87120438	-1.92331374
H	-1.17942595	2.76709580	-1.69791794
H	1.41354001	2.17104220	1.03759563
H	2.13018870	1.56138599	-0.45949081
H	0.64263237	0.48789287	-2.22298741
H	-1.10206556	0.39246643	-1.95988774
H	-0.03464442	-1.80159271	-1.54883146
H	2.49442244	-0.00187448	1.40381229
H	2.19633937	-0.93057609	-0.88763648
H	1.57115448	-2.04463887	0.33372417
H	-0.93850505	-2.18252873	0.73613399
H	-2.03762102	-1.17806399	-0.21207134
H	-2.11235476	1.27381504	0.11587883
H	-1.13974214	2.06047058	1.35259771
H	0.50659716	0.59126240	2.74801111
H	0.53735238	-1.14617002	2.44603252
H	-1.72366977	-0.21555427	2.06279302
H	1.61459017	4.61733675	-1.15650368
C	-0.99712169	21.11770248	-2.44163132
H	-0.99725062	22.19973946	-2.44174623
H	-0.99718887	20.75755119	-1.42108226
H	-0.11333226	20.75751495	-2.95192504
H	-1.88086843	20.75740433	-2.95188904

SI.13.5 **3-TS•H+**

C	6.02445889	2.35476089	2.65975499
H	7.11947584	2.41927791	2.65574789
C	4.16893816	3.83225608	2.99463010
H	3.68245602	3.53088188	2.06044412
O	5.57656813	3.66598010	2.86507893
C	5.55295897	1.44370496	3.80985808
H	5.97028208	1.82658005	4.74902391
O	5.98747683	0.12299600	3.70183492
C	4.02123690	1.50027502	3.86953306
H	3.61556602	1.17377305	2.89883089
O	3.55088401	0.64684403	4.88322783
C	3.56951904	2.94696593	4.12010908
H	3.93885589	3.27950001	5.09877014
O	2.15860605	3.00687909	4.06452894
C	3.92370200	5.32616186	3.27165103
H	3.09913301	5.44290876	3.97855711
H	4.84227705	5.73997784	3.69664001
C	6.64381599	0.22026700	-2.10368609
H	7.15646982	-0.23508599	-2.95929694
C	6.99380302	1.91049004	-0.49219701
H	6.15677786	2.50443912	-0.86995202
O	7.54653597	1.15396202	-1.55962205
C	6.38761187	-0.83700502	-1.00222099
H	7.37634420	-1.10609603	-0.60770500
O	5.80831194	-2.06474209	-1.37995601
C	5.59604406	-0.12909900	0.11245400
H	4.65642691	0.26235399	-0.30391401
O	5.30720806	-1.01195300	1.17332494
C	6.41851187	1.05420005	0.65220302
H	7.23367119	0.66706800	1.27466297
O	5.54336786	1.87452602	1.42190802
C	8.09758663	2.85659790	0.00450800
H	8.52080154	3.37145209	-0.86729699
H	7.66340208	3.59713006	0.67963803
O	9.08916664	2.19008303	0.73048902
C	3.99303389	2.68871903	-5.96950293
H	3.96927094	3.28573394	-6.89012718
C	4.72908306	2.87023592	-3.73543692
H	3.70368409	2.64140105	-3.42687988
O	4.67583513	3.46076894	-5.03152514
C	4.82242823	1.40480399	-6.19028711
H	5.81983805	1.74512303	-6.49866819
O	4.37060308	0.57433403	-7.21330500
C	4.94886923	0.64565098	-4.86010218
H	3.94325995	0.32192701	-4.54249620
O	5.78263378	-0.47536901	-5.01492310
C	5.53337383	1.55019605	-3.75944805
H	6.58227587	1.77019894	-3.98951197

O	5.45286512	0.85507202	-2.51005912
C	5.29290199	3.98281789	-2.84098411
H	5.18273687	3.71489096	-1.79059100
H	6.35128880	4.13957787	-3.07727599
O	4.57345915	5.18323278	-3.00729895
C	-1.26490605	1.64848602	-6.22063494
H	-2.23785710	1.54393101	-6.71770096
C	0.51295698	3.27563906	-6.17565107
H	0.45473301	3.47423005	-5.09823513
O	-0.78287601	2.90279698	-6.63014889
C	-0.27941701	0.58112800	-6.72401094
H	-0.12236100	0.74520701	-7.79794121
O	-0.69729501	-0.73867202	-6.59103298
C	1.04020202	0.81597197	-5.97520304
H	0.84095800	0.81960797	-4.89307022
O	1.94025004	-0.22317900	-6.27570486
C	1.61501598	2.19160295	-6.35714912
H	1.96756196	2.16559911	-7.39474201
O	2.67936206	2.49749994	-5.47014713
C	0.82116801	4.59711790	-6.92541695
H	1.36409903	4.39179993	-7.84932280
H	-0.13959301	5.05706215	-7.16611290
C	-4.23894310	0.67305100	-1.86807299
H	-5.24279785	0.45624799	-1.48305595
C	-3.20596004	2.23949790	-3.33822799
H	-2.44102097	2.48677111	-2.59118104
O	-4.38714123	1.81335199	-2.67033005
C	-3.72611594	-0.50625098	-2.71827006
H	-4.46611023	-0.72427499	-3.49866009
O	-3.55601692	-1.68471897	-1.99118900
C	-2.40155196	-0.10429100	-3.39319205
H	-1.66460299	0.13575201	-2.61374998
O	-1.92094898	-1.17972398	-4.16690207
C	-2.63126397	1.14248395	-4.25798893
H	-3.34990191	0.90613502	-5.05257702
O	-1.40338898	1.59067297	-4.82054520
C	-3.55485511	3.51147103	-4.11920404
H	-2.74526501	3.73583698	-4.81702900
H	-4.48131180	3.33805299	-4.67971611
O	-3.66993809	4.62556219	-3.27587891
C	-3.04469609	1.35749900	3.28416491
H	-3.34349990	1.34891701	4.34056902
C	-3.93227005	2.11993289	1.20131302
H	-3.05385208	2.73457003	0.96712703
O	-4.08533192	2.01363707	2.61409092
C	-2.93343496	-0.08994300	2.76660705
H	-3.90057993	-0.58443803	2.95916796
O	-1.89571595	-0.82843900	3.35149789
C	-2.67431808	-0.05601800	1.25655901
H	-1.70818901	0.44088799	1.10620296
O	-2.63192105	-1.35635698	0.71514499

C	-3.75661802	0.74475801	0.52983499
H	-4.70353699	0.19228600	0.56986701
O	-3.33302593	0.90632999	-0.81769001
C	-5.20644712	2.80279207	0.68386501
H	-5.29169893	3.79284906	1.14955604
H	-5.14038277	2.91500211	-0.40010199
O	-6.34390211	2.02782297	0.93608302
C	1.41533899	3.47790790	5.17233419
H	2.06291199	4.01459122	5.87949181
C	-0.45052400	3.86607504	3.77574897
H	0.11685900	3.54886508	2.90011811
O	0.46626499	4.40037918	4.73121881
C	0.67712098	2.33984303	5.90723181
H	0.05790300	2.80628490	6.68402386
O	1.50520599	1.43343997	6.56611776
C	-0.23809500	1.62657797	4.90261602
H	0.38277701	1.19242799	4.10339499
O	-0.96206802	0.60316199	5.55427122
C	-1.21799302	2.62451196	4.26845598
H	-1.97695303	2.90370798	5.01057816
O	-1.82804406	2.04699111	3.12207699
C	-1.39005101	5.00756407	3.37180209
H	-2.24106407	4.59294605	2.82825089
H	-1.74606895	5.50877810	4.28042889
O	-0.76905203	5.91281891	2.50098491
N	3.53047204	6.09262609	2.11830497
N	1.60718596	5.50545311	-6.13863611
H	5.70164394	-0.25703701	2.84826398
H	4.20139790	-0.06704200	4.97786093
H	5.10792017	-1.89722705	-2.02287412
H	5.33861780	-1.91253197	0.81795299
H	9.54031277	1.57778704	0.13707200
H	3.49873805	0.20895299	-6.96377897
H	5.65189123	-0.80800402	-5.91510105
H	4.54729223	5.36647511	-3.96883512
H	-1.08134401	-0.88962001	-5.70415115
H	1.39708400	-0.98735797	-6.53552723
H	-3.16830897	-1.49250400	-1.11613095
H	-2.23083091	-1.99266696	-3.73748207
H	-4.41039896	4.46856117	-2.67808700
H	-1.63619101	-0.42675701	4.20009708
H	-2.17809892	-1.91581905	1.35967004
H	-6.40231419	1.89253497	1.88998199
H	2.19286609	1.10640597	5.95510101
H	-0.37808999	0.23831600	6.23751497
H	0.00640600	6.28859520	2.93852401
C	1.18492603	6.39929581	-5.21565819
C	2.34869003	6.85313082	-4.61542416
N	3.37812495	6.23526478	-5.24197912
N	2.92824292	5.44840002	-6.13561392
N	2.34259892	6.68104982	2.11495590

N	2.19355106	7.31981182	1.02429104
C	4.17861509	6.36429501	0.96369302
C	3.29511309	7.16012478	0.24986300
C	2.63498497	7.76889277	-3.46716690
C	3.36779094	7.78977823	-1.10615802
N	2.51506305	7.10258389	-2.13029909
C	1.09040403	6.99269915	-1.68970203
C	0.40223899	5.74199486	-2.21634889
C	0.68515497	4.42577124	-1.48005497
C	2.12324595	4.27923584	-0.97088802
C	0.49269599	3.27743912	-2.48793507
C	0.67440200	1.91085505	-1.83087397
C	2.34074712	2.91623902	-0.30377299
C	2.08917308	1.77265000	-1.28007901
C	-0.29019201	1.75084305	-0.66474801
C	-0.29343599	4.22907305	-0.31851399
C	1.37369394	2.79858303	0.86901402
C	-0.05815900	2.87129092	0.34354600
H	0.15288700	6.65565205	-5.09736490
H	5.15703106	5.99327087	0.74718499
H	1.97619402	8.63956070	-3.48224401
H	3.66869497	8.09857368	-3.59016705
H	3.01943588	8.82308102	-1.02749300
H	4.39981985	7.76950693	-1.45907295
H	0.56812400	7.88149214	-2.04803491
H	1.04618394	7.01353216	-0.59802699
H	0.66774201	5.60064602	-3.25962090
H	-0.67455202	5.91666222	-2.18020606
H	2.33589911	5.04384422	-0.22982199
H	2.82358193	4.36562109	-1.80303299
H	1.24254298	3.37355995	-3.27949405
H	-0.49798900	3.33632803	-2.94022393
H	0.49011600	1.13545597	-2.58192301
H	3.37772202	2.85629702	0.05223800
H	2.82709694	1.77443004	-2.08025694
H	2.18855190	0.82046199	-0.75448602
H	-0.11178700	0.78249902	-0.19292600
H	-1.32410097	1.77174306	-1.00091600
H	-1.31624496	4.27220821	-0.69977999
H	-0.18246201	5.02576590	0.41955101
H	1.55214202	3.61717391	1.56828499
H	1.53304601	1.86081004	1.40360606
H	-0.75894397	2.75027204	1.17238998
H	2.91782808	6.15298510	-2.24337792
C	-0.99712199	21.11770248	-2.44163108
H	-0.72796798	20.06960487	-2.42786288
H	-0.80027902	21.55491638	-1.47171795
H	-2.05124998	21.21391678	-2.66992998
H	-0.41794699	21.63228798	-3.19631004

SI.13.6 **3-out•H⁺**

C	4.87661219	2.12803102	2.71107006
H	5.84522295	2.63291001	2.60825896
C	2.61361289	2.65341806	3.10979295
H	2.32594204	2.11534500	2.19550610
O	3.94142890	3.13885689	2.97396898
C	4.92710686	1.12202895	3.88485503
H	5.22750998	1.66306400	4.79006481
O	5.86781693	0.10871700	3.70336699
C	3.52378511	0.53407902	4.07983017
H	3.24985600	-0.02501500	3.17042899
O	3.46984506	-0.31685999	5.19772816
C	2.50103402	1.65837896	4.27965403
H	2.70533800	2.16685200	5.23074007
O	1.22670102	1.05375504	4.29545784
C	1.70585799	3.87197208	3.30312109
H	0.78019297	3.58800006	3.81127310
H	2.24741912	4.61505604	3.89483500
C	6.12255383	0.27006099	-2.05101204
H	6.86096716	0.02254800	-2.82450891
C	5.60809183	2.02771997	-0.56851500
H	4.60319185	2.13643289	-0.99524897
O	6.49854517	1.52596498	-1.56143999
C	6.16173077	-0.78315502	-0.93520200
H	7.18176699	-0.81395203	-0.52039802
O	5.80831194	-2.06474209	-1.37995601
C	5.19223690	-0.34396401	0.17018300
H	4.17265987	-0.34027600	-0.24187100
O	5.25726414	-1.22136605	1.27189696
C	5.53325415	1.07344794	0.63789302
H	6.50001478	1.06117904	1.15521801
O	4.50039387	1.48907304	1.51479805
C	6.13685513	3.40785599	-0.16189700
H	6.21354723	4.02617121	-1.06576097
H	5.43306780	3.87298799	0.53026402
O	7.36437607	3.33698893	0.50621098
C	3.05444288	0.03753600	-6.30557585
H	2.94659710	0.19712000	-7.38749695
C	3.53166890	1.27854395	-4.35317421
H	2.61645007	0.87805498	-3.89115191
O	3.36114597	1.30170703	-5.76715183
C	4.16943979	-0.98958898	-6.03927517
H	5.09254122	-0.66825002	-6.53594923
O	3.84307003	-2.24410009	-6.55598116
C	4.41199398	-1.05660796	-4.52456379
H	3.50382495	-1.44295502	-4.03665590
O	5.50732279	-1.89267802	-4.22674704
C	4.69840908	0.35122100	-3.98270512
H	5.62069893	0.73230702	-4.44052696

O	4.82222319	0.29886901	-2.57875991
C	3.72761297	2.72764206	-3.88493395
H	4.78918982	2.96547699	-3.79739499
H	3.28834796	3.37890410	-4.65600920
O	3.16137505	2.99847293	-2.62894797
C	-2.19105792	-0.51691800	-6.31698179
H	-3.15657806	-0.41752401	-6.82947588
C	-0.21207000	0.73314399	-6.00599384
H	-0.31460300	0.65180600	-4.91479397
O	-1.49310601	0.66775602	-6.61548519
C	-1.43290901	-1.74804199	-6.84764814
H	-1.31916797	-1.66219997	-7.93579578
O	-2.11343002	-2.93883109	-6.59814882
C	-0.03700800	-1.76295900	-6.20151711
H	-0.15425000	-1.87854898	-5.11310196
O	0.76070899	-2.80061007	-6.71303892
C	0.67769498	-0.43442199	-6.47310781
H	0.87944198	-0.35117400	-7.54959106
O	1.88680995	-0.48544300	-5.74323082
C	0.37229300	2.10251904	-6.36470079
H	1.42949903	2.00511909	-6.63063288
H	-0.18388601	2.50361609	-7.21471882
C	-5.19283295	-0.13950500	-1.97113502
H	-6.16638184	0.12868300	-1.54222095
C	-3.58733106	0.81010300	-3.45212197
H	-2.76567292	0.69519198	-2.73192596
O	-4.81924486	0.95907402	-2.75311708
C	-5.33156204	-1.41361403	-2.83391905
H	-6.11044121	-1.24818003	-3.58777189
O	-5.72684622	-2.53254890	-2.10100198
C	-3.98977590	-1.66132605	-3.53392696
H	-3.21476603	-1.81994700	-2.76781106
O	-4.05260706	-2.77951097	-4.38762283
C	-3.61887097	-0.42907399	-4.36233711
H	-4.37266397	-0.28889099	-5.14814997
O	-2.34904909	-0.67575097	-4.94025087
C	-3.39720392	2.10105300	-4.25435305
H	-2.56577396	1.98874903	-4.95055485
H	-4.31870413	2.29079795	-4.82139111
O	-3.09434199	3.19724703	-3.42970896
C	-4.09383297	-0.55836201	3.15305090
H	-4.44022083	-0.62573099	4.19384909
C	-4.54740906	0.70131898	1.20025396
H	-3.51346898	1.05593503	1.11510205
O	-4.86372089	0.45187601	2.56961203
C	-4.30293989	-1.90046299	2.43605399
H	-5.36437511	-2.17779493	2.48065209
O	-3.57266498	-2.93526196	3.03017497
C	-3.89975309	-1.71834803	0.96402597
H	-2.83217001	-1.45920205	0.91585398
O	-4.13421106	-2.90157795	0.23378500

C	-4.71233177	-0.57327598	0.35339999
H	-5.77143097	-0.86092901	0.33361599
O	-4.23413897	-0.35486701	-0.96253902
C	-5.50085878	1.80797696	0.73819798
H	-5.32331610	2.69284892	1.36128104
H	-5.29257679	2.05448794	-0.30377200
O	-6.84260798	1.40688097	0.79525101
C	0.31330499	1.39200401	5.30708694
H	0.80214202	1.95768905	6.11174917
C	-1.47058201	1.66504800	3.76242995
H	-0.81726903	1.44986606	2.90421295
O	-0.69312900	2.23332191	4.81594419
C	-0.25293499	0.07132400	5.85807180
H	-0.94765198	0.28577200	6.67884111
O	0.74725902	-0.75208998	6.37766695
C	-1.01608205	-0.61693698	4.71729898
H	-0.31098300	-0.82918400	3.89833903
O	-1.62699795	-1.81046200	5.14139509
C	-2.10244298	0.33261001	4.19768810
H	-2.83056593	0.51108199	4.99896622
O	-2.72186208	-0.28889501	3.08978105
C	-2.52064204	2.71325588	3.34078097
H	-3.52607489	2.30904293	3.47284102
H	-2.40059590	3.58469510	3.99627995
O	-2.42540693	3.06807303	1.99021697
N	1.33503199	4.43316221	2.03515601
N	0.28527701	3.02904701	-5.26676989
H	5.65799904	-0.39026299	2.89012599
H	4.31234884	-0.79095900	5.24361277
H	5.65786695	-2.05210090	-2.34065509
H	5.35219479	-2.11472392	0.91366303
H	7.99690294	2.90488791	-0.08067400
H	2.89497995	-2.41898799	-6.42208576
H	5.44448996	-2.66669106	-4.80490398
H	2.28181291	2.59386396	-2.58803296
H	-2.60960388	-2.87405300	-5.76168489
H	0.18579499	-3.56906700	-6.83657503
H	-5.13242722	-2.65397096	-1.33545399
H	-4.63346004	-3.42847610	-3.96460390
H	-3.75891089	3.20652604	-2.72828007
H	-2.81618905	-2.56125998	3.51100707
H	-3.94655108	-3.64201999	0.82781899
H	-7.02671194	1.13535798	1.70283604
H	1.55258501	-0.68101001	5.83561516
H	-1.02565205	-2.23594093	5.76852322
H	-1.60205305	3.56366992	1.84599805
C	0.22094600	4.37359095	-5.34219885
C	0.31497100	4.79276180	-4.02992201
N	0.41792300	3.69140005	-3.25959897
N	0.40612000	2.65223908	-4.01084280
N	0.07201800	4.52490282	1.67084897

N	0.01304000	4.99604511	0.47907600
C	2.13807511	4.86004210	1.03883898
C	1.26517797	5.22221184	0.03063600
C	0.34548399	6.17017221	-3.45654297
C	1.50425196	5.71644592	-1.35688102
N	0.22540800	6.13742304	-1.98539805
C	-0.24249300	7.42956877	-1.41060603
C	-1.63240194	7.88520622	-1.85101104
C	-2.85360789	7.23959494	-1.16267300
C	-2.80221891	5.70838404	-1.16569197
C	-4.09459591	7.69565105	-1.94520497
C	-5.36571598	7.13415384	-1.31236994
C	-4.08577299	5.14798784	-0.54210597
C	-5.30516291	5.60737896	-1.33676100
C	-5.47468519	7.63410616	0.12609200
C	-2.97843504	7.72096777	0.28642899
C	-4.20592594	5.63677216	0.89965099
C	-4.25580597	7.16177082	0.91544598
H	0.12093700	4.90558481	-6.26313305
H	3.20274711	4.85874224	1.11933696
H	-0.48003101	6.74398422	-3.88080311
H	1.27986097	6.67708015	-3.72091007
H	2.20024204	6.55990887	-1.35715497
H	1.94080198	4.89919996	-1.94166303
H	0.49292001	8.18669415	-1.69593000
H	-0.23186800	7.32210684	-0.32613099
H	-1.75033402	7.77480507	-2.92990994
H	-1.67875898	8.95827389	-1.65094995
H	-1.97246301	5.35309505	-0.55143899
H	-2.70457697	5.32984304	-2.18672800
H	-4.02359581	7.35082197	-2.98010612
H	-4.14109802	8.78686714	-1.95799804
H	-6.23288488	7.47694111	-1.88568294
H	-4.02383089	4.05596113	-0.53790897
H	-5.24072790	5.25810623	-2.37000990
H	-6.21245289	5.18990088	-0.89834601
H	-6.38523579	7.24779892	0.58589101
H	-5.53127193	8.72467995	0.13714500
H	-3.00358605	8.81272411	0.31507599
H	-2.11924005	7.38484383	0.87122297
H	-3.35895109	5.27420712	1.48139799
H	-5.11560917	5.23513079	1.34734297
H	-4.32932711	7.51765680	1.94807899
H	-0.47288099	5.41551924	-1.73116100
C	-0.99712199	21.11770248	-2.44163108
H	-1.16927695	22.18406677	-2.50407410
H	-0.95769602	20.81950569	-1.40209401
H	-0.05861400	20.87568474	-2.92307401
H	-1.80351496	20.59333992	-2.93771291
